# *Giardia* and Vilém Dušan Lambl

**DOI:** 10.1371/journal.pntd.0002686

**Published:** 2014-05-08

**Authors:** Marie Lipoldová

**Affiliations:** Laboratory of Molecular and Cellular Immunology, Institute of Molecular Genetics, Academy of Sciences of the Czech Republic, Prague, Czech Republic; Georgetown University, United States of America

Giardiasis is a neglected disease that spreads worldwide from the Arctic [1] to the Tropics [2]. It affects nearly 2% of adults and 6% to 8% of children in developed countries worldwide. Nearly 33% of people in developing countries have had giardiasis [3]. The disease is caused by a binucleated flagellated protozoan parasite *Giardia lamblia* (synonyms: *G. intestinalis* and *G. duodenalis*) that inhabits the small intestine in humans and some other mammals. *Giardia* has two morphologically distinct developmental stages, the trophozoite and the cyst. The trophozoite resides and replicates in the upper small intestine and is responsible for disease manifestations; in the lower parts of the intestine, encystation occurs, and a thick protective cyst wall is formed in this process. The cyst is the infectious, environmentally resistant stage responsible for transmission [4]. People become infected with *Giardia* by swallowing *Giardia* cysts found in contaminated food or water. Cysts are instantly infectious once they leave the host through feces. An infected person might shed 1,000,000,000–10,000,000,000 cysts daily in their feces, and this might last for several months [3]. However, swallowing as few as ten cysts might cause someone to become ill. *Giardia* is mainly passed as anthroponosis, but zoonotic transmission also occurs. Clinical giardiasis is varied and ranges from asymptomatic passage of cysts to abdominal cramps, nausea, acute or chronic diarrhea, malabsorption, weight loss, and failure of children to thrive in both subclinical and symptomatic disease [4], [5]. Pathology in giardiasis is understood to arise in several ways. These include breakdown of the epithelial barrier, defects in the epithelial brush border, increased secretion of chloride ions, and hypermotility of the intestinal smooth muscles [5]. The factors determining the variability in clinical outcome in giardiasis are still poorly understood. However, host factors (such as genotype, type of microbiota in the gut, immune status, nutritional status, and age) as well as differences in virulence and pathogenicity of *Giardia* strains are recognized as important determinants for the severity of infection [5].

Although the actual host defense mechanisms responsible for controlling *Giardia* infections are poorly understood, many studies have demonstrated the development of adaptive immune responses as well as innate mechanisms in humans and other animals. T cells and mast cells are necessary to control the infection, whereas the role of B cells in defense is contradictory [5]. *G. lamblia* has the ability to undergo extensive variation of the surface coat antigens, called variant-specific surface proteins (VSPs), which are unique, cysteine-rich zinc finger proteins. The patterns of infection in humans and animals fail to show the expected cyclical waves of increasing and decreasing numbers of parasites expressing unique VSPs. However, selection by immune-mediated processes is suggested because switching occurs at the same time that humoral responses are first detected. This process likely delays the effectiveness of the antibody response [6].

The first description of *Giardia* had been attributed to Antonie van Leeuwenhoek, who in his letter to the Royal Society on November 4, 1681, described the presence of microorganisms in his stool [7]. However, the first microscopic drawing of morphological characteristics identifying the parasite *Giardia* (Figure 1A and 1B) was provided by Vilém Dušan Lambl in 1859 (Text S1) [8], analysing the stool of a child. The similarity with the modern photographs of *Giardia* is obvious (Figure 2). He called the observed microorganisms *Cercomonas intestinalis*. In 1888, the name was changed to *Lamblia intestinalis* by Raphael Anatole Émile Blanchard. In 1915, the species was renamed to *Giardia lamblia* by Charles Wardel Stiles to commemorate the work of Alfred Giard and Vilém Dušan Lambl.

**Figure 1:**
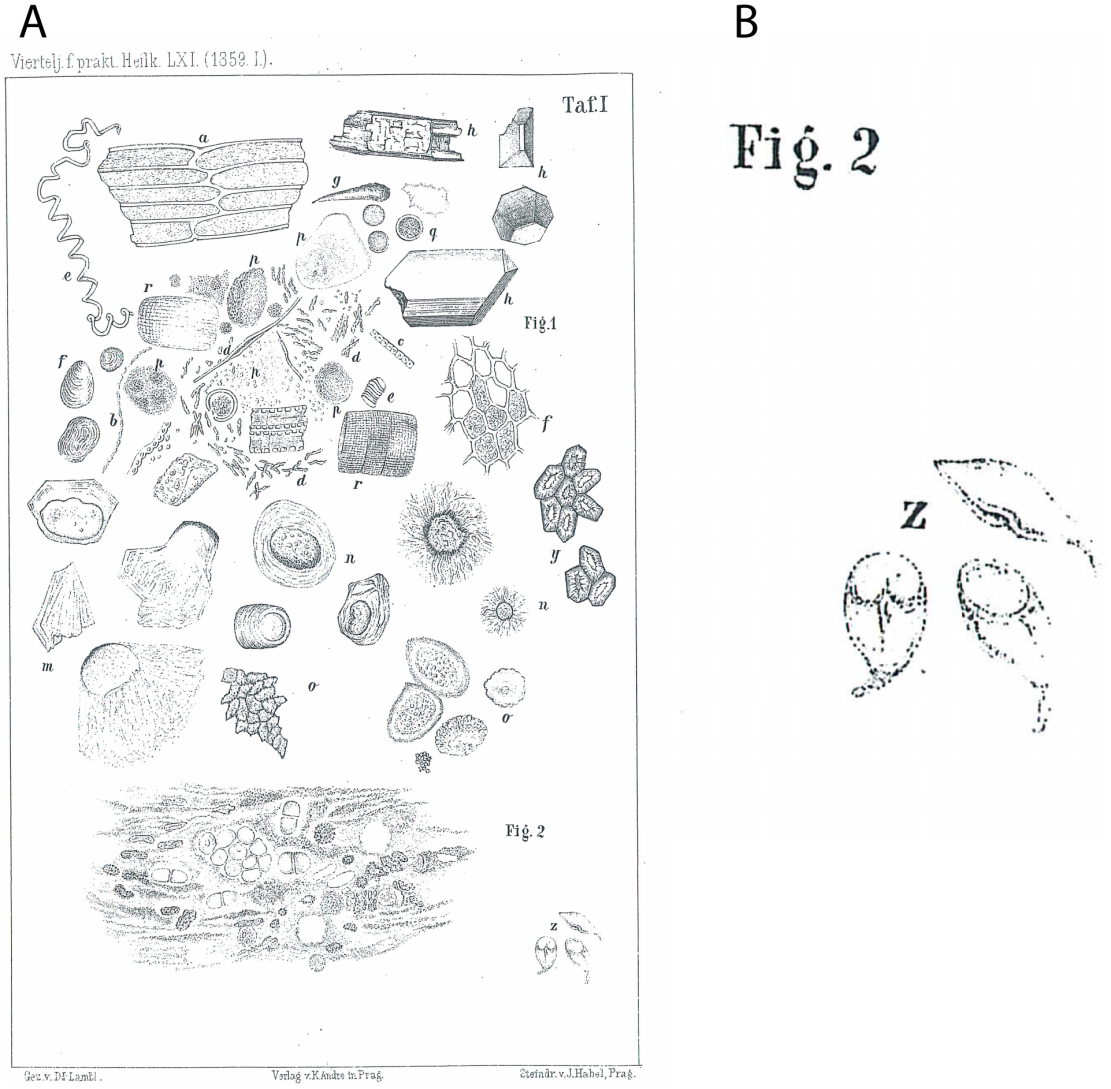
First microscopic drawing of morphological characteristics identifying the parasite *Giardia* (*Cercomonas intestinalis*). (A) Whole Table I. (B) Detail—*Giardia* (*Cercomonas intestinalis*). Reproduced from the following publication: Lambl V (1859) Mikroskopische Untersuchungen der Darm-Excrete. Beitrag zur Pathologie des Darms zur Diagnostik am Krankenbette. Vierteljahrschrift für die praktische Heilkunde. Herausgegeben von der medicinischen Facultät in Prag 61: 1–58 (Table I) [8].

**Figure 2:**
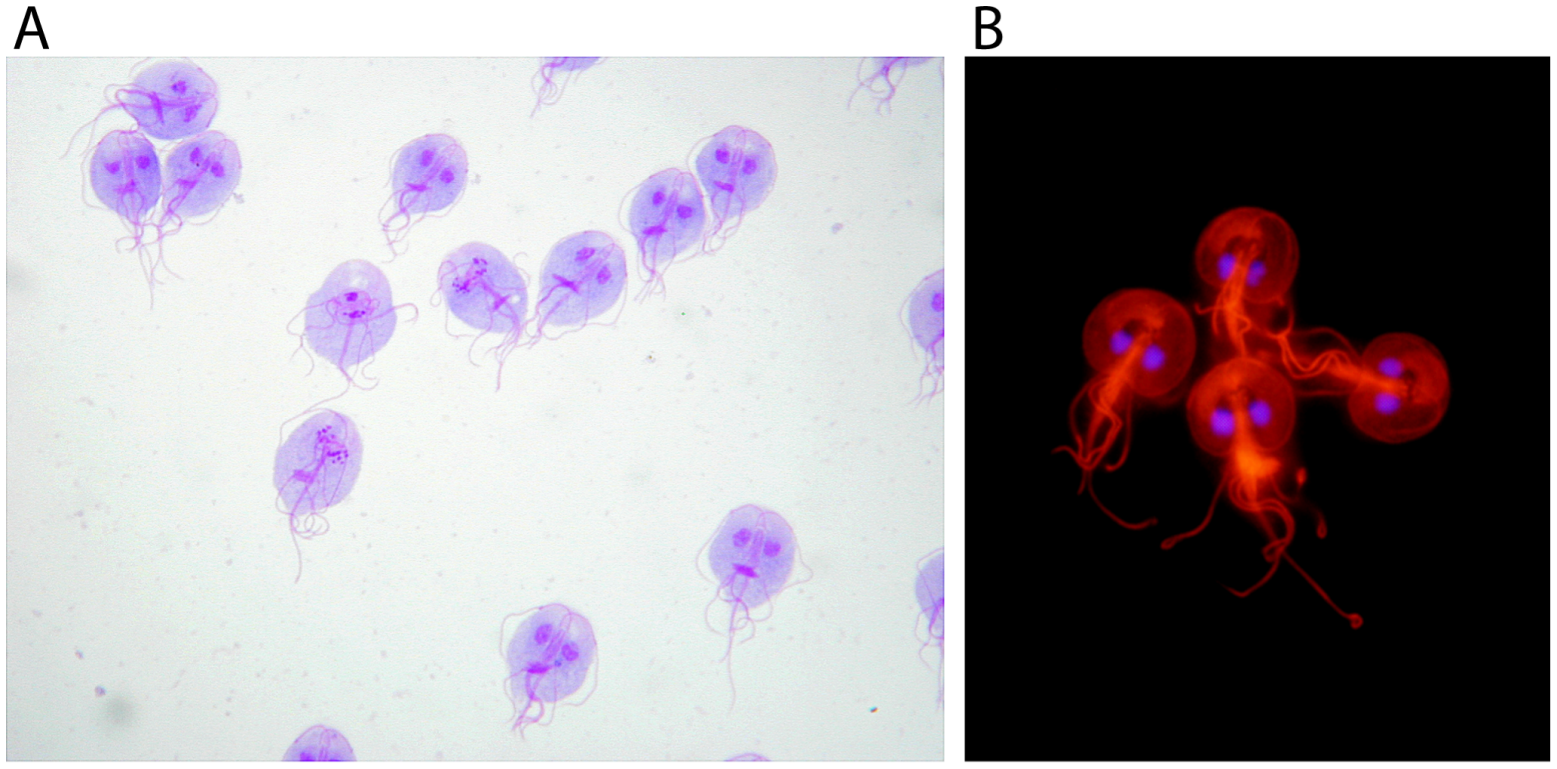
*G. lamblia* visualized using current techniques. (A) *G. lamblia* (synonyms: *G. intestinalis* and *G. duodenalis*) trophozoites in a Giemsa stained cell culture, 100× magnification. Photo: Eva Nohýnková, Department of Tropical Medicine, 1st Faculty of Medicine, Charles University in Prague and Hospital Bulovka, Czech Republic. (B) Indirect fluorescent antibody staining of *G. lamblia* (synonyms: *G. intestinalis* and *G. duodenalis*) trophozoites. Red: The microtubule skeleton detected by a monoclonal antibody against acetylated α-tubulin (clone 6-11B-1, Sigma); blue: DNA stained by 4′,6-diamidino-2-phenylindole (DAPI), 100× magnification. Photo: Eva Nohýnková, Department of Tropical Medicine, 1st Faculty of Medicine, Charles University in Prague and Hospital Bulovka, Czech Republic.

Because its association with disease was not constant [9], the question as to whether *Giardia* was a pathogen or a commensal was debated for many decades. In 1981, the World Health Organization (WHO) [10] added *Giardia* to the list of parasitic pathogens, but Koch's postulates were fulfilled only in the year 1987 when Nash and colleagues demonstrated the pathogenicity of *Giardia* infections in humans by the inoculation of volunteers with trophozoites [11]. *Giardia* may be the most common pathogenic parasitic infection in humans [4]; however, no vaccine to prevent the disease in humans is available [3].

In the next part, I would like to draw attention to Vilém Dušan Lambl, the physician and scientist who first scientifically documented this parasite by microscopy drawing. Vilém Dušan Lambl (Figure 3) was born on December 5, 1824, in Letiny, near Plzeň (now in the Czech Republic; in 1824, the Czech lands belonged to the Austrian Empire) [12]. He was one of eleven children; only eight reached adulthood. Around 1845, he enrolled at the University of Prague. He studied medicine but was also interested in zoology and linguistics, particularly Slavic languages and literature. He travelled extensively, especially in Bosnia, Croatia, Serbia, and Montenegro, conducting research on the southern Slavic languages, culture, fauna, and flora, and published many articles about his trips. He was a skilled draughtsman and accompanied his articles with multiple illustrations. He was also involved in the Czech patriotic and democratic movement, which later became an obstacle to his scientific career in the Austrian Empire.

**Figure 3:**
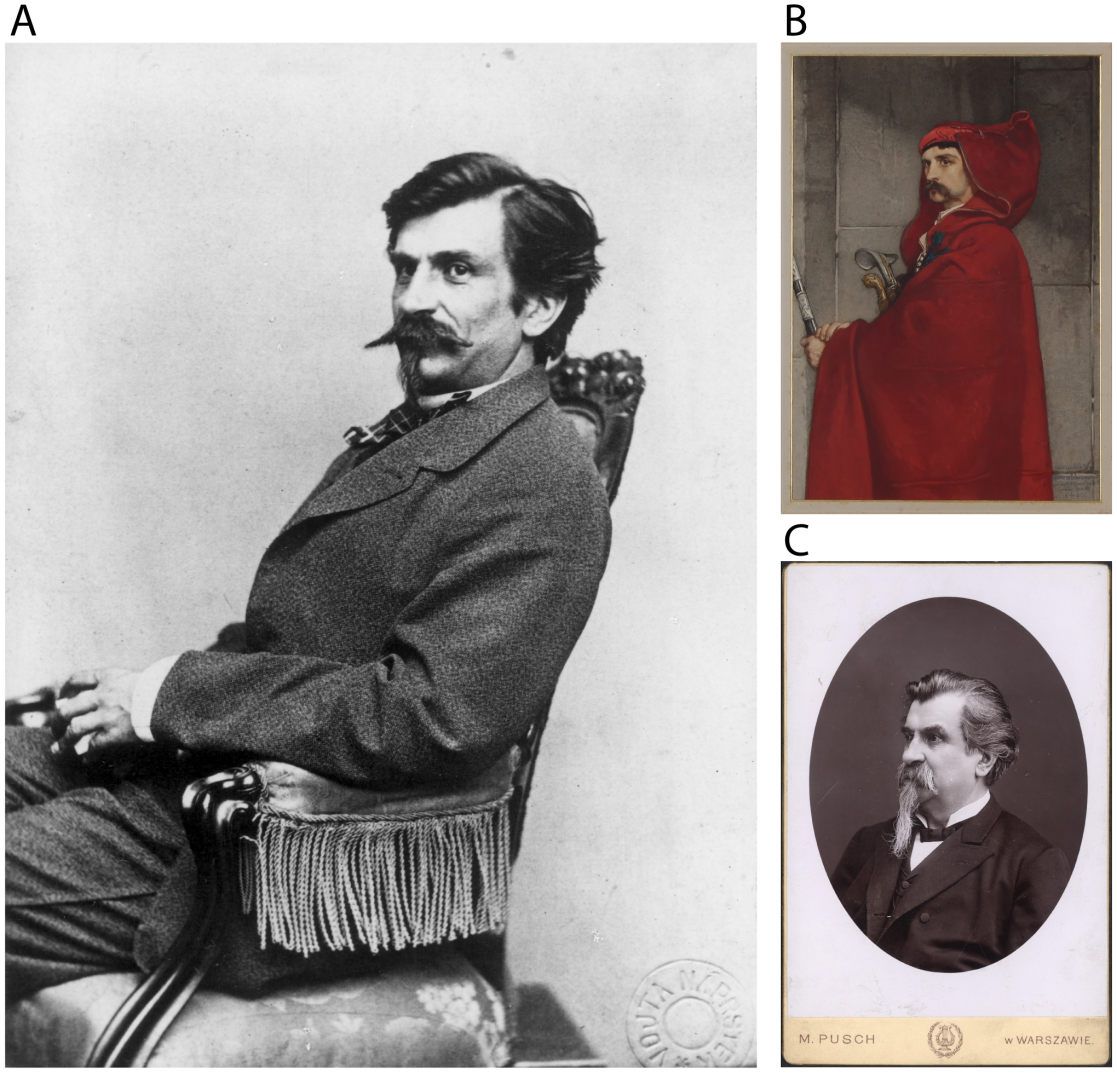
Vilém Dušan Lambl. (A) Photo of V. D. Lambl in his younger years. The original document is stored in the Literary Archive of the Museum of Czech Literature (PNP), Prague, Czech Republic. (B) Sketch of inhabitant of Herzegovina (Portrait of Dr. Dušan Lambl as Saracen) [*Hercegovec na črtách (Podobizna MUDr. Dušana Lambla jako Saracéna)*] by painter Jaroslav Čermák (1861). Watercolor on paper (29×20 cm). The original painting is in the City Gallery Prague, Czech Republic. (C) Photo of V. D. Lambl during his years in Warsaw. Original document is stored in the Literary Archive of the Museum of Czech Literature (PNP), Prague, Czech Republic.

He earned his degree in medicine on January 23, 1851, and became an assistant of Professor Václav Treitz (famous for the description of the ligament of Treitz, Treitz's hernia, and Treitz's uremic colitis) at the Institute for Pathological Anatomy at the University of Prague. In 1856, he became associated professor (Privatdozent in German) of pathological anatomy and histology at the Medical School (University of Prague), where he taught histopathology and pathology of cancer, and worked at Josef Löschner's children's hospital. In 1856, he described Lambl's excrescences (Text S2) [13], small fibrin deposits on the aortic valve. They originate as small thrombi on endocardial surfaces (where the valve margins contact) and have the potential to embolize to distant organs. He was also the first to introduce detection of bladder cancer using analysis of cells present in urine (Text S3) [14]. During his work at Josef Löschner's children's hospital, he described in the stool of a five-year-old girl *Giardia* (Figure 1), which he called *Cercomonas intestinalis* (Text S1) [8]. His fame attracted to his lectures young physicians arriving from the Unites States, Denmark, the Netherlands, Germany, and Russia. However, after the crushing of the Revolution (1848–1849) in the Austrian Empire, Felix Schwarzenberg, minister-president (1848–1852), Alexander Bach, head of internal affairs (1849–1859), and Johann Kempen, the general-gendarmerie-inspector (1849–1859), created a neoabsolutist police state. The country was infested with Secrete Police, and prisons were full of political prisoners. Lambl and two of his brothers were under Secrete Police surveillance, and when Lambl applied for a professor position, he was told by the authorities that he could not become a professor in any place within the Austrian Empire [12]. Therefore, he accepted in 1860 a position at Kharkiv University (Ukraine) and in 1861 became a full professor. Ukraine was at that time under Russian rule with a regime similar to that in Austria, but at least there were no obstacles to Lambl's scientific work. In 1871, he moved to Warsaw (in Poland, which was at that time under Russian rule) and worked there as a professor of therapy at Warsaw University and as a hospital director [12], [15]. In the years 1845–1875, he published more than 100 papers [15]. During his lifetime, Lambl published in various languages (German, Czech, Russian, Polish, French, and Italian) and used different first names; therefore, it is sometimes difficult to find all of his publications. He was baptized Wilhelm (this name is also on his work about *Cercomonas intestinalis* (Text S1) [8]), later changed Wilhelm to the Czech version, Vilém, and added a southern Slavic name, Dušan. Some papers are published under the name Vilém Dušan Fedorovič Lambl, some articles about culture are signed “Vilém Dušan”, papers about Lambl's excrescences (Text S2) [13] and detection of cancer cells in urine (Text S3) [14] are signed just “Dr. Lambl,” and in sources in Russian he is listed as ДушaH ФедopoBич ЛяMбль/Dushan Fedorovich Lyambl' [15].

He died in Warsaw on February 25, 1895. He left in his testament 20,000 Austro-Hungarian Guldens for the Fund for Support of Czech Students at the Medical Faculty of the University in Prague and at the Technical University in Prague.

## Supporting Information

Text S1 **Article with the first microscopic drawing of** ***Giardia*** **.** Full text of [8].
(PDF)

Text S2 **Article with the first description of Lambl's excrescences.** Full text of [13].
(PDF)

Text S3 **Article with the first description of detection of bladder cancer using the analysis of cells present in urine.** Full text of [14].
(PDF)

## References

[1] Hotez PJ (2010) Neglected infections of poverty among the indigenous peoples of the arctic. PLoS Negl Trop Dis 4: e606. 20126272 10.1371/journal.pntd.0000606 PMC2811175

[2] Kline K, McCarthy JS, Pearson M, Loukas A, Hotez PJ (2013) Neglected tropical diseases of Oceania: review of their prevalence, distribution, and opportunities for control. PLoS Negl Trop Dis 7: e1755. 23383349 10.1371/journal.pntd.0001755 PMC3561157

[3] CDC (Centers for Disease Control and Prevention) (2012) *Giardia*—Epidemiology & Risk Factors. Available: http://www.cdc.gov/parasites/giardia/epi.html. Accessed 22 August 2013.

[4] Hill DH, Nash T (2011) Intestinal flagellate and ciliate infections. In: Guerrant RL, Krogstad DJ, Maquire JH, Walker JH, Weller PF, editors. Tropical Infectious Diseases. Principles, Pathogens and Practice. 3nd edition. Philadelphia (Pennsylvania): Churchill Livingston. pp. 623–632.

[5] Solaymani-Mohammadi S, Singer SM (2010) *Giardia duodenalis*: the double-edged sword of immune responses in giardiasis. Exp Parasitol 126: 292–297. 20599999 10.1016/j.exppara.2010.06.014 PMC2933943

[6] Nash TE (1997) Antigenic variation in *Giardia lamblia* and the host's immune response. Philos Trans R Soc Lond B Biol Sci 352: 1369–1375. 9355129 10.1098/rstb.1997.0122 PMC1692022

[7] Dobell C (1920) The discovery of the intestinal *Protozoa* of man. Proc R Soc Med 13 (Sect Hist Med) 1–15. 10.1177/003591572001301601 PMC2151982 19981292

[8] Lambl V (1859) Mikroskopische Untersuchungen der Darm-Excrete. Beitrag zur Pathologie des Darms zur Diagnostik am Krankenbette. Vierteljahrschrift für die praktische Heilkunde. Herausgegeben von der medicinischen Facultät in Prag 61: 1–58 Available: Text S1.

[9] Kulda J, Nohýnková E (1995) *Giardia* in humans and animals. In: Kreier JP, editor. Parasitic Protozoa. Volume 10. San Diego (California): Academic Press. pp. 225–422.

[10] WHO Expert Committee (1981) Intestinal protozoan and helminthic infections. WHO Tech Rep Ser 58: 666–671. 6800138

[11] Nash TE, Herrington DA, Losonsky GA, Levine MM (1987) Experimental human infections with *Giardia lamblia* . J Infect Dis 156: 974–984. 3680997 10.1093/infdis/156.6.974

[12] Kilian J (2005) [110th anniversary of the death of the physician and patriot Vilém Dusan Lambl (1824–1895)]. [in Czech] Cas Lek Cesk 144: 847–748. 16389761

[13] Lambl V (1856) Papilläre Excrescenzen an der Semilunar-Klappe der Aorta. Wien Med Wochenschr 16: 244–247 Available: Text S2.

[14] Lambl V (1856) Über Harnbalsenkrebs. Ein Beitrag zur mikroskopischen Diagnostik am Krankenbette. Vierteljahrschrift für die praktische Heilkunde. Herausgegeben von der medicinischen Facultät in Prag 49: 1–32 Available: Text S3.

[15] Popov MA (1896) Professor Dushan Fedorovich Lyambl', his professional and writing activity: resources for the history of the Kharkov University. Kharkov: Printing House Adolf Darre. [In Russian] Available: http://escriptorium.univer.kharkov.ua/handle/1237075002/425. Accessed 8 April 2014.

