# Supplementary material for: Giardia and Vilém Dušan Lambl
Source: PLoS Negl Trop Dis. 2014 May 8;8(5):e2686. doi: 10.1371/journal.pntd.0002686 (PMC4014406; doi:10.1371/journal.pntd.0002686)
Supplement: Text S1 — Article with the first microscopic drawing of Giardia . Full text of [8]. (PDF) [file pntd.0002686.s001.pdf]

Original - Aufsätze.

Mikroskopische Untersuchungen der Darm-Excrete.

Beitrag zur Pathologie des Darms und zur Diagnostik am Krankenbette.

Mit lithographirten Abbildungen.

Von Dr. Wilhelm Lambl, Docenten an der Universität zu Prag.

Die mikroskopische und chemische Untersuchung der Fäcalmassen gehört zu den schwierigsten Aufgaben der wissenschaftlichen Praxis und es wird unzweifelhaft noch unendliche Mühe kosten, die vielfachen Fragen über physiologische und pathologische Vorgänge der ersten Wege zum befriedigenden Abschluss zu bringen. Ist doch das Capitel über die morphologischen und chemischen Veränderungen der Nahrungsmittel und über Resorption der Nahrungsstoffe nicht erledigt, und die letzten Untersuchungen der anerkanntesten Forscher auf diesem Gebiete, Brücke, Donders, Kölliker, Moleschott, u. A. haben gezeigt, dass zu der bisherigen Kenntniss über die einfachsten Gebilde (z. B. Epithelialzellen) noch immer neue anatomische Thatsachen beigebracht werden können. Um nur die Einleitung des Stoffwechsels gehörig zu begreifen, müssen wir vorerst Import, Transito und Ausfuhr der Rohstoffe vollständig in Evidenz halten; der Gegenstand würde einen Atlas füllen, der für die physiologische Morphologie und Chemie des Verdauungsactes ebenso wichtig und unerlässlich wäre, als auf Grundlage desselben erst die Pathologie und zwar nicht etwa nach einem oder dem anderen Befunde mitten im Krankheitsverlaufe, sondern nach einer vollständigen Reihe von Beobachtungen des anomalen Vorganges mit erschöpfender Detailforschung verzeichnet werden könnte. Die Aufnahme von Zufäl-

ligkeiten bei einer solchen Untersuchung wird selbstverständlich nicht ohne Werth und Belehrung sein, da man ja nur auf diesem Wege zur wahren Einsicht und zur Unterscheidung des Wesentlichen vom Accidentellen gelangt; je genauer und je mehr man von den zahllosen Bildern, die sich der Wahrnehmung kund geben, kennen gelernt und verzeichnet hat, desto sicherer und rascher wird man über ihren Werth entscheiden können und selbst auf die Gefahr hin, der müssigen Befriedigung einer gelehrten Neugier geziehen zu werden, wird man minutiöse Untersuchungen nicht aufgeben, sofern es sich um die Consequenz der Methode und nicht so sehr um die Constatirung eines gewünschten Resultates handelt. — Thomas fand auf seinen Linsenschliffen unerklärliche Figuren von Scheibenform, und Czermak erklärte sie — durch einen glücklichen Zufall belehrt — für die Abdrücke des Fliegenrüssels (Viertelj. f. p. H. Bd. 44. J. 1854, S. 176.) — In einem englischen Lehrbuch (R. Druitt, the Surgeon's Vade Mecum 1856 p. 569.) wird unter den Abbildungen der Harn-Sedimente eine nette Figur mitgetheilt und für Krystallisation von Harnsäure gehalten, die ein geübter Uroskopiker als ein zufällig in das Excret hineingerathenes Bart-Fragment eines Flaumfederchens erkennen muss. — Ich wurde einmal von einem zierlichen Gegenstande vexirt, der mir im Darmexcrete wiederholt zur Ansicht kam, und worüber mir Niemand Aufschluss zu geben vermochte; nach langem Herumsuchen fand ich im Süsswasser denselben Gegenstand wieder und Grund und Boden dazu, nämlich die Daphnia pulex, deren Fühlhörner das fragliche Object bildeten. — Ist auch die definitive Lösung der Aufgabe mit unabsehbaren Schwierigkeiten verbunden, so können diese manchen Missgriff entschuldigen und die Beiträge auf Nachsicht Anspruch machen, ohne jedoch die Unternehmung zurückzuweisen. Denn jetzt, wo der Wetteifer der Mikrographen an den delicatesten Probeobjecten organischer Wesen — z. B. an den Diatomeen seine Kräfte misst, und die Anzahl von Streifen und Punkten, die bei Pleurosigma lacustre auf den tausendsten Theil einer Linie fallen, bestimmt anzugeben bemüht ist, sollten da die Angelegenheiten der Auswurfstoffe, die sich vom physiologischen Interesse bis zur forensischen Bedeutung erstrecken, von der schärfsten Kraft, die wir besitzen, unangegriffen bleiben? Der Arzt gründet seine Diagnose zum guten Theile auf die physikalischen Merkmale der Ausleerungen: die feinsten Details hierüber und folglich

die genaueste Einsicht in die inneren Vorgänge liefert ihm das Mikroskop. Da es sich hier weniger darum handelt, die bekannten Thatsachen in Ordnung zu bringen, als vielmehr die aus eigenen Untersuchungen gewonnenen Resultate mitzutheilen, so mag hier die Erwähnung hinreichen, dass viele einschlägige Gegenstände in den physiologischen Lehrbüchern (von Donders, Funke etc.) mit mehr oder weniger Ausführlichkeit, die pathologischen Fragen am umfassendsten von Höfle (Chemie u. Mikroskop am Krankenbette 1848) von Lindsay (Associations-Journal) und von Virchow (Archiv Bd. V. S. 370) behandelt wurden, und dass auf sonstige Quellen im Texte verwiesen werden wird.

Ein Cumulativ-Bild des gewöhnlichen Befundes von Fäcalmassen Fig. 1. möge eine weitläufige Einleitung ersetzen und dem Erkennen von Pflanzentrümmern (*a-g*), Muskelresten (*r*), Fett und Pigment (*o-q*), unvollständig verdauten Käse- und Fettklümpchen bei Milchkost (*m*, *n*), Krystallen von Phosphaten (*h h*) zu Hilfe kommen.

Was zuvörderst die *geformten Elemente* betrifft, so ist eine genauere Betrachtung der vergänglichen Schutz- und Secretgewebe eben sowohl von physiologichem Interesse, als auch von einigem Belang bezüglich der seit Jahren besprochenen Frage über die Natur von reinem Schleim und von Eiter. Die *Schleim-* und *Eiterzellen der Darmexcrete variiren* sowohl in ihrer *Form*, *Grösse* und *Anzahl*, als auch (Fig. 2—5.) in der speciellen Eigenthümlichkeit oder in der *Physiognomie*, worunter wir die Beschaffenheit ihres Umrisses, Vorhandensein oder Mangel des Kerns, Klarheit, Opacität oder granuläre Beschaffenheit des Zelleninhaltes verstehen, woran sich die Einwirkungen des Wassers und anderer Reagentien als experimentelle Veränderungen des Ausdruckes dieser Elemente anschliessen. In vielen Schleimzellen ist der Kern ohne Zusatz eines Reagens sichtbar, in anderen vollkommen entwickelten ist zur Darstellung des Kerns der Zusatz von Essigsäure nothwendig, bei einigen reicht der Zusatz von Wasser hin, den Kern kenntlich zu machen. Destillirtes Wasser bewirkt im Allgemeinen eine Anschwellung der Zellenwand durch Imbibition und eine Abnahme der granulären Beschaffenheit des Zelleninhaltes von der Hülle gegen den Kern zu, zuweilen jedoch bleibt dieser Effect aus; dasselbe macht die Zellenmembran niemals durchsichtiger, und bewirkt auch im Inneren des Zellenraumes kein Verschwinden des opaken Aussehens oder ein Hellerwerden des granulären Inhaltes, der sich wohl zertheilt, aber nicht auflöst. — Bei Anwendung der

Essigsäure ist auf die Stärke derselben, auf die Dauer der Einwirkung und auf die Temperatur besondere Rücksicht zu nehmen, um den Eintritt des Effectes gehörig würdigen zu können; darnach wechseln die Erscheinungen in jedem gegebenen Falle. Im Allgemeinen bemerkt man bei langsamer Einwirkung einer verdünnten Flüssigkeit auf die Schleimzellen, dass die Zellhüllen leicht schwellen und immer heller und durchsichtiger werden, bis sie als zarte farblose Bläschen von dem scharfmarkirten Kern sich weit abgehoben haben. Der Kern erscheint hiebei in Folge des Hellerwerdens des ursprünglich matt-opaken, durch Essigsäure almälig aufgeklärten Zelleninhaltes deutlicher und schärfer begränzt; in vielen Zellen jedoch wird trotz des Hellerwerdens des Zelleninhaltes kein Zellenkern sichtbar, an einzelnen Zellen wird sogar anscheinend keine Veränderung hervorgerufen. Manchmal schwillt die Zellenmembran bedeutend, und der Zelleninhalt wird heller bis auf eine wolkige Trübung oder einen feinkörnigen Saum in der Umgebung des Kerns, welcher nicht weicht; zuweilen bleibt sogar nach Einwirkung der Essigsäure eine gleichmässige Opacität im Zellenraume zurück, ohne jedoch deutlich granulär zu erscheinen.

Ebenso variirt auch der *Zellenkern* in Bezug auf Grösse, Form, Anzahl und Physiognomie; in der Einzahl kommt ein Nucleus bald central, bald peripher im Zellenraume vor, sein Umriss ist mehr oder weniger scharf, die Form rund, eiförmig, dreieckig, 8-ähnlich, unregelmässig gekerbt, das Innere desselben entweder klar und glänzend, oder matt opak, oder körnig und punctförmig getrübt. Die nächste Umgebung des Nucleus erscheint bald hell, bald wolkig getrübt, oder fein körnig halonirt. Sehr häufig kommt ein gekerbter, sternförmiger, oder selbst vielfach getheilter Kern zur Beobachtung und dann stellt derselbe eine Gruppe von 3—5 Körperchen dar, deren zwei oder drei eine gemeinschaftliche Begränzung haben; auch bei diesen Elementarkörperchen könnte man die besondere Physiognomie wie bei den vollkommenen Kernen hervorheben, man vermisst daran höchstens das granuläre Aussehen. — Bei Anwendung von *Essigsäure und Erwärmung* der Objecte bemerkt man, wenn die Einwirkung im Kalten nicht erfolgt, die Aufhellung des Zelleninhalts, die Verdünnung der Zellenmembran, die scharfe Abgränzung und Theilung des Zellenkerns; alsbald *löst sich die Zellenhülle* auf, schwindet vollständig, und der Kern bleibt allein zurück, bald ohne opake Umhüllung, bald

in eine feinkörnige Masse eingebettet; in diesem Falle treten in dem Kern sehr häufig deutliche, punktförmige, jedoch helle *Kernkörperchen* auf.

Eiterkörperchen ergeben bei der mikroskopischen Untersuchung, gleichviel ob ein dysenterisches Excret (Fig. 5.) oder gonotorischer Abscesseiter von anderen Orten vorliegt, im Ganzen dieselben *Resultate wie die vollkommen entwickelten Schleimzellen*, und es muss mit besonderem Nachdruck die bekannte Thatsache wiederholt werden, dass der *histologische Normalcharakter* in den beiden Excret-Flüssigkeiten wo nicht identisch, wenigstens optisch *nicht zu unterscheiden* ist. Worin beruht also der Unterschied zwischen Schleim und Eiter, wie man gewöhnlich zwei augenscheinlich differente Flüssigkeiten zu benennen pflegt? — Der Unterschied ist nicht histologischer Natur, sondern mit dem blossen Auge, also *in der Gesammtheit einer gegebenen Excretmasse* erkennbar und das Folgende dient zur näheren Begründung dieses Ausspruches. Man findet sehr leicht den Unterschied zwischen kernhaltigen wohlgebildeten Eiterzellen und zwischen kernlosen unvollkommenen Schleimzellen, oder im concreten Falle: vergleicht man die entwickelten Zellen eines guten Normaleiters mit den jungen Zellen des Schleimes im Reiswasserstuhle der Cholera, so wird man unmöglich übersehen, dass die Eiter-Zellen mit deutlichen ein- oder mehrfachen Kernen versehen sind oder dieselben nach Zusatz von Essigsäure alsbald erkennen lassen, die Schleimkörperchen hingegen keine Kerne enthalten, auf Essigsäure-Zusatz dieselben eben so wenig auftreten, der granuläre Inhalt dabei unerheblich bleibt. Es ist jedoch bei diesem Vergleich zu bemerken, dass man es in einem solchen Falle mit irrationalen Grössen zu thun hat, deren Verrechnung schon insofern Schwierigkeiten bietet, als die Coëfficienten in Bezug auf ihr Alter und den gewichtigen Ausdruck ihrer Entwickelung nicht auf einer Stufe oder in paralleler Reihe stehen. Abortive, junge und rudimentäre, ebenso rasch producirte als auch rapid entleerte Gewebselemente (Cholera) können unmöglich den Vergleich mit solchen aushalten, die sich langsam und unbehindert entwickelt, ihre vollkommene Reife erlangt und sich etwa noch darüber hinaus metamorphosirt haben, bevor sie der Elimination anheim gefallen sind (Dysenterie). — Man kann also in dieser Beziehung die *Extreme der Entwickelungsreihe* wohl abstecken und die für sie charakteristischen Formen genau definiren, wie sie in einzelnen typischen Formen zur Beobachtung

kommen, dabei aber niemals übersehen, dass dieser abstracte Charakter der Flüssigkeit an keine Gränzen seines Vorkommens, namentlich nicht an besondere Krankheitsvorgänge strenge gebunden ist, dass er in extensiver und intensiver Beziehung häufigen Schwankungen unterliegt, und dass somit keiner Zellenform an und für sich eine ausschliesslich pathognomonische Bedeutung zukommt.

Dieser Betrachtung gemäss kann man die oben gewählten Excretmassen der Cholera und der Dysenterie als Extrême bezeichnen und den histologischen Charakter derselben folgendermassen formuliren: Die serösen oder Reiswasser-Stühle der Cholera charakterisiren sich besonders durch unvollständige Formen von Schleimzellen und von embryonalen, abortiven Körperchen, die einer reichlichen Menge von formloser Flüssigkeit beigemengt erscheinen; bei sorgfältiger Untersuchung findet man zuweilen auch kernhaltige Zellen, oder es tritt der Kern, wiewohl meistentheils nur ein einfacher, selten ein zusammengesetzter, bei Einwirkung des Wassers oder der Essigsäure zum Vorschein. Bei der eitrigen Dysenterie nimmt man hauptsächlich normale, vollständig entwickelte, ein- und mehrkernige Zellen wahr, die sehr dicht gedrängt erscheinen, da sie offenbar in einer verhältnissmässig geringen Menge von formloser Flüssigkeit suspendirt sind; aber häufig befinden sich diese Zellen in einem ungewöhnlichen Zustande von Zerfall, Einkerbung und Auflösung, so dass sie alle ihre normalen Kennzeichen aufgeben, unter die regelmässige Grösse fallen, weder im Wasser, noch in Essigsäure sich wesentlich verändern, namentlich den Zellenkern eben so wenig ersichtlich werden lassen, als die Klärung ihres Granular-Inhaltes gestatten. — Die mikroskopische Schilderung aller dieser Vorgänge und der mannigfaltigen Zustände der zarten Gewebselemente hat demnach einen weiten Umfang und allerdings eine gewisse Berechtigung in der physikalischen Diagnostik am Krankenbette; sie liefert ein mit eigenen Mitteln errungenes Resultat über alles Dasjenige, was in ihr Bereich gefallen war, und beantwortet das fragliche Object mit ihrer eigenen Sprache. Wenn das Mikroskop auf nicht-histologische Fragen keine andere als eine histologische Antwort gibt, so ist an der ungenügenden Lösung der Fragesteller Schuld, nicht das Instrument. So viel steht also fest, dass die mikroskopische Eiterprobe eine Illusion ist, und dass man mit bewaffnetem Auge die Elemente des Eiters und die des Schleimes in gemischten Flüssigkeiten, wie

sie bei Darmentleerungen vorzukommen pflegen, nicht so exact zu unterscheiden vermag, als man sie auf den blossen Blick abzuschätzen gewohnt ist. Reinen Schleim und ausgezeichneten Eiter in grossen Mengen unterscheidet auch das blosse Auge hinreichend sicher, während das Mikroskop denselben Unterschied in den kleinsten Quantitäten, die dem blossen Auge unerreichbar sind, sicherstellen wird, sonst aber, wo es sich um gemischte und zugleich serös getränkte Flüssigkeiten handelt, nur die Identität der zelligen Elemente nachweisen kann. Wir wissen, dass einfacher *Schleim* gewöhnlich durchscheinend, geruch- und farblos, klebrig und zähe, faserähnlich ausziehbar, sich mit Wasser nicht leicht mischt, sondern darin suspendirt bleibt oder zu Boden sinkt, sich leicht mit Luftbläschen mengt und dieselben hartnäckig festhält, u. s. w. Diese optischen Eigenschaften erkennt das Mikroskop auch an den kleinsten Quantitäten gewöhnlichen Schleimes und zeigt die Veränderungen, welche die Reagentien hervorrufen, z. B. die feinkörnige Trübung und Coagulation der formlosen Flüssigkeit, auch an solchen Schleimpartikelchen, welche das blosse Auge überhaupt gar nicht bemerkt. — Der *Eiter* ist auf der anderen Seite in seiner exquisiten Eigenthümlichkeit opak, rahmähnlich dick und gelblich, tropfbarflüssig, mit Wasser mehr emulsionsfähig, daher mischbar und weniger luftbläschenhaltig: und alle diese Eigenschaften ersieht man aus den kleinsten optischen Bildern eben so leicht, als man den Einflus von Reagentien im mikrochemischen Maassstab zu prüfen vermag.

Ungewöhnlich cohärente *Schleimklumpen* werden zuweilen durch den Stuhlgang entfernt, ohne irgend eine begründete Vermuthung über die Art ihres Zustandekommens zu gestatten. Am 23. April 1858 brachte die Mutter eines ganz gesunden 5 Jahre alten Mädchens ein solches Object in das Kinder-Spital zur Untersuchung in einem Schälchen, und gab an, die Masse sei von dem Kinde abgegangen, ohne dass sie vor oder nach irgend etwas Besonderes bemerkt hätte, da das Kind die ganze Zeit hindurch vollkommen normale Entleerungen habe. Der Gegenstand war ein an *anderthalb Zoll langer Hohlcylinder* einer gleichmässig zusammenhängenden Masse, deren äussere Fläche glatt, beinahe serös glänzend, die innere zart gewulstet und flockig, die Farbe blassgraulich, Weichheit und Dehnbarkeit wie bei gewöhnlichem Schleim im Sputum erschien. Sollte das Ganze aus dem Darmcanal gekommen sein, so konnte es auf den ersten Blick nur für den Abguss des Dickdarms gehalten

werden, dessen weites Caliber so ziemlich dem Cylinder entsprach. Interessant war der gleichmässige Zusammenhang, der sich in dem schleimähnlichen Stoff bei näherer Prüfung und Zerrung mittelst einer Nadel ergab. — Die *mikroskopische Untersuchung* (Fig. 2.) zeigte in jedem losgelösten Partikelchen Schleimzellen und Kerne aller möglichen Formen und Grössen, theils isolirt und gleichmässig in der Intercellularsubstanz suspendirt, theils in Gruppen von vier- bis achtzelligen Elementen vertheilt, theils auch, — was für Schleimsubstanzen ein charakteristisches Merkmal im optischen Ausdruck abgibt — beim Ausziehen in einen faserähnlichen Streifen, entsprechend dem Zuge verlängert, und zu ovalen und spindelförmigen Körperchen umgestaltet (Fig. 3). Die kleineren Formen der einzelnen Elemente variirten zwischen 0,006—0,009 Mm.; ihre Oberfläche war grösstentheils rauh, der Inhalt wegen stärkerer Lichtbrechung als die der umgebenden Substanz, einseitig schattirt, in vielen Zellen feinkörnig und durch Granulirung verdunkelt, im Ganzen mit einem gelblichen Schimmer versehen, welcher bei der Mucinmetamorphose von Epithelialgebilden, im stagnirenden Schleim und dessen Formbestandtheilen zumal nach einiger Zeit und beim Beginn der Zersetzung aufzutreten pflegt. Zahlreiche kerbige und kantige, durch Schrumpfung und Zerfall verschiedentlich missgestaltete Körperchen, die zwischen den wohlerhaltenen befindlich sind, so wie einzelne colossale Formen von granulären Körperchen, die aus den Aggregaten und Gruppirung der vorigen entstanden sind, sprechen ebenfalls für einen gewissen Grad der Maceration und eine Stufe der Umsetzung der Substanz, welche Erscheinungen nach 24 Stunden Ruhe an sämmtlichen Elementen deutlich wahrnehmbar erscheinen. — Beigemengt fanden sich vor: Bruchstücke von verdauter quergestreifter Muskelfaser, Stärkmehlkügelchen und lose Faserfragmente von Pflanzenstoffen; auffallend war der Mangel an Darmepithel, an Gallenpigment und Fetten, wovon auch unter dem Mikroskope keine Spur nachzuweisen war. In der eigentlichen Schleimflüssigkeit, welche die geformten Elemente in reichlicher Menge umschliesst, tritt alsbald die feinpunktförmige Körnung auf, und ist nach 24stündiger Ruhe des Objectes an jedem Partikelchen wahrnehmbar. — Dem unbewaffneten Auge könnte ein solcher cohärenter Schleimklumpen um so mehr für ein höheres Gewebe imponiren, als die äussere Fläche des Cylinders — der präsumtive Abguss des Dickdarmrohres — vollkommen glatt und glänzend, auf den

ersten Blick eine seröse Haut vortäuscht, während die zottig-weiche Innenfläche eine serös infiltrirte Schleimhaut nachahmt.

Die *Schleimkörperchen und Zellen* sind, wiewohl als Gewebselemente des Schleimes nicht unwichtig, dennoch *keine wesentlichen Bestandtheile der Schleimflüssigkeit.* So wie die Blutflüssigkeit, die Lymphe und der Chylus, eben so ist der Schleim in einer gewissen Menge sehr wohl ohne Formelemente denkbar und an bestimmten Orten gewiss in dieser Art vorhanden. Das Blut circulirt wahrscheinlich in manchen serösen Gefässen, deren Caliber zum Durchgang von Blutzellen nicht hinreicht; von der Lymphe und vom Chylus gilt dasselbe; der Schleim kommt oft auf ansehnlichen Strecken in einer Weise vor, wo die mikroskopischen Bilder sich einzig und allein auf die *zarte Streifung einer homogenen hyalinen Substanz* beschränken, ohne irgend wie geartete Körperchen wahrnehmen zu lassen. So besteht der glasähnlich durchscheinende Pfropf im Muttermunde aus einer gallertigen zähen Schleimmasse, die vollkommen formlos erscheint; nicht blos bei jungen Mädchen, sondern auch bei Schwangeren findet man zuweilen den Verschluss der Portio cervicalis uteri durch einen Schleimpfropf bewerkstelligt, worin selbst der gewöhnliche Befund von colloiden Kernen und Zellen vermisst wird, die von den sogenannten Ovula Nabothi stammen und sonst am häufigsten beigemengt vorzukommen pflegen. In ähnlicher Weise entdeckt man oft auf anderen Schleimhäuten, und in einzelnen Fällen selbst im diarrhoischen Excrete ganz unerhebliche Schleimkörperchen der hyalinen Masse beigemengt, und ihr völliger Abgang dürfte um so weniger auffallend erscheinen, als derselbe mit den übrigen Erscheinungen (Geruch- und Farblosigkeit der Masse etc.) übereinstimmen wird.

Es frägt sich also zunächst, woher diese geformten Elemente stammen? — Bei der Ungleichförmigkeit der *Schleimkörperchen und Zellen*, die man in einem glasähnlichen zähen Secret findet, und bei den geringen, ja unzureichenden Differenzen, welche eine blennorrhoische Flüssigkeit und der Abscesseiter bietet, gelangt man durch fortgesetzte Untersuchungen dahin, die Quelle der geformten Elemente des Schleimes nicht in einem bestimmten Gewebe zu suchen, welches allein dieselben zu liefern die Aufgabe hätte, sondern vielmehr den Ausspruch zu wagen, dass man es hiebei mit *sehr verschiedenen Gewebstheilen und deren Derivaten zu thun habe.* Abgestossene *Epithelialzellen* verlieren die den Kern umgebende Enchymhülle

frühzeitig, und der *Kern* bleibt in der Schleimflüssigkeit als einfaches, durch schleimige Metamorphose oft verändertes Körperchen zurück. — Sehr häufig bemerkt man Cylinderzellen mit zwei Zellenkernen, von denen der eine, dem Basalende näher gestellte, zum Entweichen vorspringt, wobei der tiefere einen weiteren Bestand und die Functionsfähigkeit der Zelle sichert. Nicht selten fand ich auch *drei bis vier ungleich grosse Kerne in einer* umfänglicheren Zelle, und dieselben Körperchen ohne besondere Zellhüllen fanden sich auch im Schleime. — Durch *Mucin-Umwandlung* und solche Veränderungen, welche der *colloiden* und *amyloiden Metamorphose* sehr ähnlich sind, werden viele dieser Kerngebilde derart alterirt, dass sie im Secrete als Modificationen von Schleimkörperchen erscheinen.

Die *Zellen der Lieberkühn'schen Drüsen* haben oft, zumal in ihren getrübten Formen, ein mit den Schleimzellen ganz gleiches Aussehen und ihre Desquamation muss als zweite Quelle dieser Formelemente angesehen werden. — Die farblosen *Enchymkörperchen der solitären und agminirten Follikel* werden bei Dehiscenz der Drüsenkapsel die Hauptmasse der Schleimkörperchen abgeben, wie dies für den Katarrh angenommen wird; ob sich aus den Elementarkörnchen und Kernen auch weiterhin im freien Zustande des Darminhaltes vollkommene Zellen ausbilden können, bleibt eine kaum zu lösende Frage. — Dagegen ist die massenhafte Production von vollkommenen Zellen in acuten entzündlichen Zuständen, namentlich bei Dysenterie, auf die anomalen Ernährungs-Verhältnisse des Substrates, d. i. der *Bindegewebselemente* der Schleimhaut, zu beziehen, und der Nachweis ist bei einiger Umsicht keinen so grossen Schwierigkeiten unterworfen. Das bekannte feingranuläre Aussehen der Schleimhaut nach Entfernung des eitrigen Ergusses erweist sich bei sorgfältiger Untersuchung als eine festhaftende, in ihrer Tiefe von blutstrotzenden Capillarschlingen vascularisirte Zellenwucherung, welche die Form von Erhabenheiten, Kolben, Cylindern, rundlichen Zöttchen und massigen Papillen und dergleichen annimmt und vorwaltend aus saftigen, dicht aneinander gelagerten und schwer zu trennenden *Formationselementen* (sphärischen, klaren Zellen mit geringer Differenzirung der Hülle, des Inhaltes, des Kernes und des Nucleolus) besteht. Entfernt man auch diese von der Schleimhaut, d. h. bis auf die Gefässschlingen hin, so überzeugt man sich eben von der *Schwierigkeit einer reinen Trennung der präexistenten und der neugebildeten Elemente*. Die ersten, die Bindegewebskörper der

Schleimhaut, befinden sich in einem Zustande von gallertiger Schwellung und Volumszunahme, und die benachbarte formlose Grundsubstanz zeigt zugleich eine solche Lockerung und schleimige Reduction, dass die ganze Physiognomie des Substrates dadurch der des umfänglichen Zellenkolbens ähnlich wird und dass es scheint, als stelle der letztere eine Verlängerung und gleichartige Fortsetzung, ein Auswachsen des Substrates dar. Bei gehöriger Isolirung der einzelnen Zellen gelangt man mittelst Reactionen zu der Einsicht, dass dieselben in lebhafter Theilung ihrer Kerne und in endogener Production begriffen sind. Die *oberflächlichsten* derselben werden durch Ueberhandnahme eines formlosen Ergusses flott gemacht und *erscheinen als Secretzellen* in der dicklichen Flüssigkeit des Darmrohres: es sind Schleim- oder Eiterzellen, die ihr Dasein der lebhaft angeregten Production der Bindegewebselemente des Substrates verdanken.

Unter den pathologischen Zuständen des *Darmepithels* ist die *Verfettung*, die im Bereiche von tuberculösen Geschwüren nicht selten zur Beobachtung kommt, erwähnenswerth (Fig. 8. A.); sie tritt als ein weisslicher Beschlag der Geschwürsränder und der inselförmigen Schleimhautreste am häufigsten im Coecum auf, und zwar zuerst als moleculäre Trübung des Zelleninhaltes, weiterhin als dunkle Punktmasse, die dem Zellenbeleg ein feingefurchtes, wie rissiges und trockenes Aussehen gibt, wobei das Epithel noch immer festhaftet, zuletzt aber in Form von Körnchen, welche die ganze Zelle erfüllen und am mikroskopischen Präparate eine schwarze Umrandung der heller bleibenden Drüsenmündungen bilden. Die Verfettung führt zum *Zerfall und zur Nekrose des Epithels*, dessen Zellen sich sofort ablösen und unter Desaggregation der Fettmolecüle zu Grunde gehen. — Die Drüsenzellen habe ich nur in untergeordneter Weise in dieser Richtung erkrankt gefunden. Nicht selten löste sich bei Behandlung dieser Zellen mit Säuren ein Theil der körnigen Trübung wie bei Incrustationen der Art auf, dass die Trübung auf theilweises Eindringen von Erden bezogen werden könnte. Möglicherweise wird über diesen bisher unbeachteten Vorgang die weitere Untersuchung nähere Aufschlüsse geben. — Auf der Schleimhaut der Gallenblase fand ich bei tuberculösen Individuen denselben Befund öfter, ja viel tiefer in die Mucosa und in das Bindegewebslager des submucösen Zellstoffs vorgedrungen; die Trübung und Verfettung bildet hier in der Höhe der netzförmigen Schleimhaut-

fältchen hellgelbe, mattglänzende Streifen auf rothbraunem Grunde; die Epithelzellen zeigen zum Theil ihre Kerne sehr deutlich und zerfallen unter zunehmender Trübung wie an den Darmgeschwüren. Die Befunde betreffen Leichen von Phthisikern mit tief gesunkener Lebensenergie und irreparablen Zerstörungen (Fig. 8. B). Ob der von Virchow (Archiv Bd. 11. S. 574) erwähnte Zustand, der auf einen intermediären Fettstrom im physiologischen Stoffwechsel bezogen wird, diesem Befunde gleichzustellen sei, halte ich für unwahrscheinlich. Was ich verbürgen kann, ist der auffallende Unterschied dieser Veränderung der Epithelialzellen und der von Erscheinungen von *Fettresorption*, welche ich bei Säuglingen sehr schön finde; die Cylinderzellen des ganzen Dünn- und Dickdarms strotzen zuweilen von hellglänzenden Fettkügelchen und Tropfen (Fig. 8. C.), die sich bis in das spitze Ansatzende der Zelle hinziehen und durch dieses hindurch gewiss unmittelbar in die zarten Capillarräume des Bindegewebes der Schleimhaut übergehen, wie Heidenhain (in Moleschott's Untersuchungen zur Naturlehre d. M. u. d. Th. 4. Bd. S. 251.) zur Evidenz nachgewiesen hat. Das subepitheliale Gewebe, welches Weber schon früher beschrieben und für den Darm als eine zweite Lage runder und ovaler Zellen angenommen hat, bietet deshalb so viel Schwierigkeiten des Nachweises, weil es nicht zu allen Zeiten in gleichmässiger Entwickelung und Mächtigkeit vorkommt. Dies gilt sowohl für den Darm als auch für die serösen Häute und die innere Gefässhaut: die der Epithelialschichte zu Grunde liegende Bindesubstanz ist hier, wie man sich bei gehöriger Vergrösserung überzeugen kann, nicht in eine eigene Begränzungs- oder Basement-Membran verdichtet, sondern von einem hyalinen, structurlosen Parenchym (Grundsubstanz) gebildet, worin in ungemein wechselnder Anzahl feine Streifen (Röhrchen), glänzende polymorphe Körperchen *(embryonale Kerne)* und einfache kernhaltige Bläschen *(Formations-Zellen)* zur Beobachtung kommen. Diese Formelemente sind im Allgemeinen, wenigstens auf dieser Stufe, *gleichwerthig für die Grundsubstanz, so wie für das Epithel*, in diesem werden sie (unter Auswachsen zu der typischen Grösse und Form) zu Schutz- und Resorptions-Gebilden (Cylinder-Zellen im Darm); in dem anderen werden sie zu Parenchymzellen und Bindegewebskörperchen, zu bleibenden und productiven Elementartheilen des Stützgewebes, welches in unmittelbarer Communication mit den mikroskopischen Resorptionsorganen die

Fortleitung der Flüssigkeiten nach dem Centrum hin ermöglicht *).

Um diese Betrachtungen für die Untersuchungen am Krankenbette zu verwerthen, wird es nach dieser cumulativen Schätzung der gewöhnlichen Formelemente hier am Platze sein, die *unterscheidenden Charaktere der Darmentleerungen* näher zu besprechen.

Die Schleimmasse, welche in Form von zarten Flocken oder von Klumpen entleert wird, stellt sowohl bei der *Diarrhöe* als auch bei der *Dysenterie* und bei der *Cholera* unter dem Mikroskop hauptsächlich feine hyaline Streifen von faserähnlicher Substanz dar, deren Zersetzung und durch Säuren eingeleitete Gerinnung sich durch eine äusserst zarte, gleichmässigvertheilte punktförmige Trübung kund gibt, — nebstdem eine verschieden grosse Anzahl von Schleimzellen und Körperchen, die das wichtigste Formelement dieser Flüssigkeit ausmachen. Bei der Diarrhöe und Dysenterie erscheinen die zelligen Elemente grösser, ausgebildet, und machen entsprechend ihrem Gewebscharakter die Reactionen vollständig durch; bei der Cholera dagegen sind dieselben viel kleiner und mit viel mehr Körnchenmasse unterlegt; selten zeigen sie hier ihre Kerne so deutlich wie dort, auch ermangeln sie der Reaction wie die vollkommeneren Schleimzellen diarrhoischer Stühle, im Ganzen scheinen sie auf einer viel jüngeren Stufe der Entwickelung zu verharren. Da sie von einer sehr ausgedehnten Schleimhautstrecke stammen, in profuser Menge und mit ungewöhnlicher Rapidität entleert werden, erscheinen sie auch unmittelbar nach

---

*) Gestielte Epithelzellen an serösen Häuten und am Endocard demonstrire ich seit mehr als zwei Jahren. Ueber das Auswachsen derselben an den Herzklappen besitze ich Präparate, welche unglaublich paradoxe Formen enthalten. — In Cystosarkomen, namentlich in Cysten-Parenchymen mit Drüsen-Neubildung, fand ich die instructivsten Belege für die unmittelbare (Röhren-) Communication der Cylinder-Epithelien mit den Bindegewebskörpern der sogen. Lagermasse. — Der helle Basalsaum der Cylinder-Zellen erweist sich bei hinreichender Vergrösserung als trichterförmiger Ansatz des Zellenkörpers mit freimündender Oeffnung; die zarten Streifen, welche daran neuerdings entdeckt und als Porencanälchen hingestellt wurden, sind an frischen und natürlichen Objecten *fetthaltiger* Zellen nicht zu sehen und für den Act der Resorption nicht unabweislich postulirt, seitdem man weiss, dass die Gegenwart von Galle (Seifenlösung) den Durchtritt des feinvertheilten Fettes durch wasserfeuchte Membranen ermöglicht, ohne dasselbe vorerst zu verseifen und alsbald wieder in der Zelle zu Oeltropfen umsetzen zu lassen.

ihrer Entstehung im Stuhle, ohne zur völligen Ausbildung und Reife zu gelangen. Es gehört jedoch eine umsichtige Untersuchung dazu, um dies zu unterscheiden. Die beigemengte Granular- und die fein-punktförmige Masse ist zum grossen Theil eine Art Emulsion, welche die molecularen Bestandtheile der eingenommenen Nahrungsmittel, das Endresultat ihrer Zertheilung, suspendirt enthält. Fettige und ölige Substanzen von verschiedener Art fehlen auch hier nicht und kommen selbst in mannigfachen Formen im Cholerastuhl vor; in geringerer Menge findet man sie zuweilen bei lange andauernder Diarrhöe.

Folgende Punkte wären bei der näheren Untersuchung ähnlicher Stuhlmassen ins Auge zu fassen, um eine Unterscheidung möglich zu machen. Die Choleraentleerung beträgt im Allgemeinen innerhalb einer Frist von 24 Stunden seit dem ersten Anfall eine enorme Quantität von Flüssigkeit und diese übertrifft in der Regel bei Weitem das diarrhoische und dysenterische Excret; allein nicht zu übersehen sind die Ausnahmen, welche zu irrigen Schlüssen verleiten könnten, als: a) Fälle von unzweifelhafter *Cholera ohne Reiswasser-Entleerungen;* es scheint sogar die Darmentleerung nicht das wesentliche Merkmal dieser Krankheit zu sein und Lindsay (Associations Journal 1854) hat festgestellt, dass zumal bei Geisteskranken derlei Fälle vorzukommen pflegen. — b) Fälle von *Diarrhöe mit ungewöhnlicher*, die Cholera-Entleerung bei Weitem übertreffender *Quantität* von dünnflüssiger Faecalmasse. c) Fälle von Katarrh und zumal von *Dysenterie* mit theilweise oder durchaus schleimigen Stühlen, deren *Menge unter der Norm* zurückbleibt; in dieser Beziehung zeichnet sich bekanntlich die gemeine Form der Dysenterie mit Tenesmus und spärlicher Darmentleerung aus.

Der wichtigste Unterschied der Darmentleerungen bei den beiden Classen von Erkrankungen beruht in dem *Verhältniss von Flüssigkeit zum Sediment:* die Wassermenge überwiegt bei der Cholera, die festen Bestandtheile beim Katarrh und bei der Dysenterie. Insofern bieten auch die chemischen und mikroskopischen Analysen einige Unterschiede. Die chemische Untersuchung erweist zumeist eine genauere Differenz bezüglich des Salzgehaltes und anderer Bestandtheile der verschiedenen Stühle, allein der ausübende Arzt findet selten Zeit und Gelegenheit, die Flüssigkeit einer näheren, namentlich einer quantitativen Bestimmung zu unterziehen; die mikroskopische Untersuchung dagegen ist kürzer und jedenfalls verwerthbar. —

Im Allgemeinen ist die *alkalinische Reaction* allen Fäcalmassen eigenthümlich. *Albumen* kommt, oft in beträchtlicher Menge, nicht blos bei katarrhalischen und dysenterischen Stühlen, sondern auch im Excrete bei anderen Krankheiten vor, und ein gewisser Antheil fehlt gewiss dem normalen Stuhlgang nicht. In einzelnen Fällen von profusen Entleerungen, besonders bei Dysenterie, ist der Albumengehalt unzweifelhaft von der Menge des Eiters abhängig und die typische Eiterflüssigkeit ist daran reicher als der gewöhnliche Schleim. Die Biliar-Reaction kommt hiebei der bei Cholerastühlen im Reiswasser-Excret gleich, nämlich nach Zusatz von Salpetersäure zu der kalten, oder tropfenweise zu der bis zum Aufkochen erhitzten Flüssigkeit tritt eine violette, röthliche, röthlichbraune und grüne Färbung ein. Die Farbe scheint von den verschiedenen chemischen Zuständen der *Gallenbestandtheile* abzuhängen und wechselt in der Scala je nach der Menge der angewandten Säure und der Erwärmung.

Für den klinischen Gebrauch ist die Rücksicht auf den vom Schwefelgehalt abhängigen *Geruch der Fäces* von Belang und in Uebereinstimmung mit den chemischen und histologischen Zuständen. Die Ge-ruchlosigkeit der Reiswasser-Stühle bei Cholera ist bedingt durch den Mangel einer eigentlichen fäecalen Beimengung; dagegen sind die Stühle bei fieberhaften Krankheiten gewöhnlich sehr übelriechend nach Maassgabe der dem Schleimgehalt beigemengten Stoffe. Je dunkler die Färbung, je grösser der Gehalt an Gallenbestandtheilen (mit Abrechnung des Blutes), desto intensiver der Geruch, desto ähnlicher die Excrete den normalen Fäces, und so bei Cholera, bei Katarrh und Dysenterie. Daher findet man bei der letzteren zuweilen, wenn die Stühle rein schleimig sind, blos einen matten widerwärtigen Geruch, gerade wie bei den Reiswasser-Stühlen im Collapsus bei der Cholera. Die molken-ähnliche Farbe und das geringe specifische Gewicht kommt wieder vorzugsweise den letzteren zu, obwohl nicht zu übersehen ist, dass im weiteren Verlaufe der Cholera die gewöhnlichen physikalischen Kennzeichen zur Unterscheidung der Cholerastühle von einfach katarrhalischen Stühlen nicht hinreichen. Die *histologischen Charaktere* der *Schleimkörperchen* in den Flocken der *Reiswasser-Stühle*, welche man gewiss nicht als irrelevant ansehen wird, (Fig. 7), beziehen sich auf 1. ihre grosse Anzahl — 2. kleinere Formen, — 3. niedrige Stufe der Entwickelung und daher — 4. unvollständige Ausbildung, —

5. Mangel der Zellenkerne und — 6. die abweichende Einwirkung des Wassers und der Essigsäure; man könnte diese Merkmale wohl als übereinstimmende Hilfsmittel zur Diagnose benützen, allein man darf sie nicht unbedingt für pathognomonisch an und für sich betrachten, da ja diese Charaktere eben nicht invariabel sind. Auch der Mangel der gewöhnlichen Trümmer von Speiseresten stimmt insofern besonders mit der Reiswasser-Flüssigkeit des Cholerastuhles überein, als die enorme Menge von Flüssigkeit, welche der Darmschlauch entleert, alle Spuren davon ausspült; kommen dergleichen dennoch vor, so sind sie wohl bezüglich der spärlichen Quantität, nicht aber in ihrer Physiognomie von solchen Speiseresten zu unterscheiden, wie sie bei Diarrhöe und Dysenterie gefunden werden.

Man hat früher dafür gehalten, dass die Entwickelung eines *deutlichen Zellenkerns* und besonders *mehrerer Kerne in einer Zelle* dem Eiter ganz eigenthümlich zukomme und den wahren Eiter von jeder anderen Flüssigkeit unterscheiden lasse; diesen Irrthum zu widerlegen, erachten wir nach dem bereits angeführten um so mehr für überflüssig, als es nebstdem auch erwiesen ist, dass diese vermeintlichen Eiterzellen den vollkommenen farblosen Blutzellen (weissen Blutkörperchen) histologisch ganz und gar ähnlich und histochemisch mit denselben völlig übereinstimmend sind.

Eben so wurde die Anwesenheit von *Oeltröpfchen* eine Zeit lang für eine Eitereigenthümlichkeit gehalten, und in der That findet man diesen Bestandtheil häufiger im Eiter als im Schleim, wo er seltener in erheblicher Menge vorzukommen pflegt. Allein abgesehen davon, dass man es im Darmcanal mit allerhand Ingestis zu thun hat, von denen Fett in verschiedener Form abzuleiten und die Fehlerquelle der Diagnostik kaum mit genügender Sicherheit zu vermeiden wäre, so könnte doch dieser Nebenbestandtheil die Gränze der Excretstoffe nicht mit gehöriger Schärfe bezeichnen und im günstigsten Falle, wo man die Oeltröpfchen als dem Eiter zugehörig nachzuweisen im Stande wäre, dürften dieselben für nichts mehr, als für Producte der Metamorphose angesehen werden, wie sie in vielen anderen flüssigen Geweben überhaupt, namentlich bei vergänglichen Texturelementen aufzutreten pflegen.

Ferner hat man einiges Gewicht gelegt auf die *Behandlung mit Alkalien und Säuren* (Zusatz und Schütteln mit Ammoniak- oder Kali-Lösung, mit Essigsäure), und auf die Veränderungen die-

ser Reactionen bei erhöhter Temperatur, wobei sich die beiden Flüssigkeiten verschiedentlich verhalten. Allein in exquisiten reinen Fällen bedarf es dieser Probe nicht, und im gemischten thierischen Excret, wo Schleim und Eiter in inniger Mischung mit Serum vorkommen, ist diese Reaction im Nachweise der Proportionsverhältnisse ebenso wenig stichhältig wie der allgemeine und der histologische Charakter. Vollgewichtig und in ihrer wahren Bedeutung tritt diese Schwierigkeit in Evidenz, sobald man es mit einem verdächtigen Darmübel zu thun bekommt, wo es von Wichtigkeit wäre zu entscheiden, ob und in wie fern eine organische Veränderung Platz gegriffen habe. Die Unterschiede des äusseren Aussehens der beiden Flüssigkeiten sind nicht immer ihrer Eigenthümlichkeit angemessen und geeignet, sie gehörig zu unterscheiden, wie schon Hassall (Microscopic Anatomy of the Human Body, vol. 1. S. 142) bemerkt, so dass es nicht ein wahres distinctives Merkmal gibt. In krankhaften Bedingungen wird daher eine dieser Flüssigkeiten stufenweise und allmälig in die andere übergehen, oder es werden sich beide mit einander so innig mengen, dass die Anwesenheit der einen die Eigenschaften der anderen völlig modificirt und das Ganze endlich allen physikalischen Unterscheidungs-Mitteln Trotz bietet.

Nebst den angeführten Oeltröpfchen findet man zuweilen *hyaline Bläschen* zwischen den Schleimfäserchen verstrickt und festhaftend, am häufigsten im dysenterischen Stuhle; ihre Natur ist da, wo sie dem Einfluss von Aether und Wärme widerstehen und demnach nicht Fett enthalten, zweifelhaft; am meisten Aehnlichkeit haben dieselben mit Blutzellenmembranen (Fig. 6, *dd*), die durch Hydrops aufgebläht und grossblasig, hell, farblos erscheinen. Bei chronischem Darm-Katarrh scheinen sie hydropisch erkrankten Schleimzellen zu entsprechen, deren Kern sich durch Verflüssigung und Aufblähung bis an die Zellenmembran erweitert und vergrössert hat.

Blutkörperchen in verschiedenen Stufen der Schrumpfung, Atrophie und sonstige Veränderungen kommen besonders in dem dysenterischen Excrete und bei Typhus (Fig. 6. a, b, c.) vor; die tuberculösen Darmgeschwüre bluten im Allgemeinen viel weniger.

Belangreicher ist die Ausscheidung von *geronnenen oder gerinnfähigen Exsudatmassen* bei schweren Fällen von Dysenterie (Fig. 5.). Wenn auch der Nachweis beträchtlicher Exsudatflocken und membranähnlicher Ausbreitungen dem blossen Auge

nicht entgehen kann, insofern der Stuhl gehörig untersucht wird, so gelingt es doch der mikroskopischen Untersuchung viel früher, und bei geringer Quantität des Excretes viel genauer, das Vorhandensein von scholligen und klumpigen Fibringerinnungen aufzuweisen. Grosse Fetzen von „croupösen Membranen“ kamen mir bisher gar nicht vor, ich zweifle jedoch an deren Möglichkeit eben so wenig als an den ausgehusteten Fibrincylindern, welche Abgussformen der Luftwege darstellen und von Vulpius irrthümlich für Venenthromben oder gar für Venen selbst gehalten wurden. — Nach einer mündlichen Mittheilung des Hrn. Dr. Dolge, practicirenden Arzte in Batavia, zeichnet sich die ostindische Dysenterie häufig durch den Abgang massenhafter starrer Exsudate, so wie durch eine enorme Hypertrophie sämmtlicher Schichten der Darmwandung aus. Der merkwürdigste Fall, den ich je gesehen, war ein *polypähnlicher Fibrinklumpen*, den man im Museo Alessandrini in Bologna als Spirituspräparat aufbewahrt hält (S. Reisebericht unter Bologna). Dergleichen cohärente membranähnliche Exsudatfetzen sind den polypösen Fibrincylindern bei acuter Bronchitis vollkommen analog, welche bei croupöser Pneumonie aufzutreten pflegen und bei completer Hepatisation eines Lungenlappens beobachtet werden, wiewohl sie auch hier nicht zu den gewöhnlichen Erscheinungen gehören. Bei einem dieser Fälle im k. k. Garnisons-Spitale zu Prag, wo die Erkrankung am dritten Tage lethal geendigt hatte, waren sämmtliche grössere Bronchen des hepatisirten Lungenflügels mit derlei zusammenhängenden Fibrincylindern wie ausgegossen, die letzteren zum Theil central erweicht und canalisirt, die peripheren Enden der herausgehobenen Stücke, das heisst, die in die feineren Bronchialzweige abgesetzte Exsudatmasse, weniger compact und allmälig in einen zähflüssig puriformen Erguss übergehend, der nebst Eiterzellen und abgestossenen Epithelzellen in der gallertigen Grundsubstanz der Flüssigkeit nur geringe Spuren von gerinnfähigem Stoffe enthielt.

Die *Formen*, unter denen sich die *Gerinnung auf der Darmschleimhaut* zu zeigen pflegt, sind so sehr mannigfach, dass man wohl von denselben in den meisten Fällen auf die in- und extensive entzündliche Affection der Mucosa Rückschlüsse zu machen im Stande ist, niemals aber sichere Anhaltspuncte über die Art der krankhaften Vorgänge in den Gewebselementen des Substrates gewinnt. In den kleinsten Quantitäten gerinnt der Erguss zu zarter *Knötchenform*, welche an der Schleimhaut

dem blossen Auge nach Entfernung der Secrete mittelst eines Wasserstrahles als feingranuläre Rauhigkeit erscheint. — Auf grössere Strecken ergossen bildet sich die Gerinnung zur *Netzform* mit kleineren und grösseren Massen, mit feineren und gröberen Balken, mit zartfaserigen Ausläufern oder *fadenähnlichen Fortsätzen* an der Peripherie, und mit knotigen oder *klumpigen Anschwellungen* an den Kreuzungsstellen des Netzwerkes. — Zuweilen lassen sich absatzweise gebildete *Schichten an einer hautartigen Ausbreitung* nachweisen, die dadurch im mikroskopischen Bilde eine beträchtliche Mächtigkeit erreicht und in ihrer Dicke ein verfilztes Stückwerk darstellt, dessen Zwischenräume von *kolbigen und feinwarzigen Klümpchen* derselben Masse ausgefüllt sind, oder einen variablen Antheil von einfachen und dunkelgranulären Zellen enthalten, wie sie sonst bei der eitrigen Form des Ergusses vorwaltend erscheinen. — Nicht selten stellt die ganze Gerinnung eine *durchlöcherte, gefensterte Membran* dar, woran jedoch die Lücken durchaus nicht regelmässig und etwa einem Organtheile, z. B. der Anordnung der Lieberkühn'schen Drüsen, entsprechend gefunden werden. In allen Fällen zeichnet sich die Substanz durch ihr Quellungsvermögen bei Zusatz von Essigsäure in der Art aus, dass die allenfalls eingeschlossenen Formelemente nachher deutlicher in die Erscheinung treten, sobald ihre opake und ungleich lichtbrechende Umgebung einigermassen aufgehellt wird.

Wir haben bereits der Quellen erwähnt, denen die profuse Zellenbildung entspringt, wie sie ganz besonders bei der eitrigen Dysenterie beobachtet wird; man bemerkt hiebei, dass die jungen embryonalen Zellen des auswachsenden Kolbens oder Cylinders klar, hyalin, saftig, — die abgestossenen dagegen trübe, opak, und von Pünktchen (Fettmolecülen) durchsetzt erscheinen. Dieses Phänomen markirt den Verfall der neugebildeten Zelle, ihre Persistenz auf der erlangten Stufe, ihren untergeordneten (secretionellen) Werth und ihre Bestimmung zur Elimination. In welcher Art der Rest der Zellenproduction zu bleibendem Gewebe verwerthet und etwa zur Hypertrophie der Schleimhaut verbraucht wird, wäre für die Dysenterie speciell noch zu untersuchen. — Höfle (l. c. S. 91) kommt bei dem Vergleich der dysenterischen mit den Cholera- und Typhusstühlen zu dem Schlusse, dass eine Uebereinstimmung der Befunde in dem Punkte zu finden sei, dass unter den drei verschiedenen Verhältnissen wesentlich nur *Krankheitsproducte*, keine eigentlichen Faecalmaterien entleert werden. Der grosse Un-

terschied des *dysenterischen* Krankheitsproductes, heisst es, beruht aber darin, dass dieses nicht ein seröses, *vielmehr ein fibrinöses Exsudat darstellt* und ausschliesslich auf der *Dickdarmschleimhaut* gebildet wird. — Nach unserer Darstellung könnten wir dem ersten Satz, der als Endresultat der mikroskopischen Untersuchung gelten soll, deshalb nicht unbedingt beipflichten, weil die Betonung der „Krankheitsproducte“ zu der Vorstellung verleiten könnte, als habe man ganz unzweideutig charakterisirte Massen nachzuweisen und deren specifische Eigenthümlichkeiten für den jeweiligen Krankheitsprocess zu vindiciren. Die mikroskopische Diagnostik ist gewiss nicht auf die Krankheitsproducte allein gerichtet und sollte sie es werden, so müsste sie sich auf höchst rudimentäre und unerhebliche Angaben beschränken, die kaum zu verwerthen wären. Das Mikroskop liefert Bilder und diese geben, so wie alle Coëfficienten der physikalischen Diagnostik, gleichviel welcher Natur sie sind, wichtige Anhaltspuncte ab, und werthvolle Belege für die Vorstellungen über die inneren Vorgänge im Organismus; *ein jedes Bild hat daher einen diagnostischen Werth*, nicht in dem Sinne, als dürfte man schematische Rubriken für die verschiedenen Krankheiten aufstellen und z. B. sagen: Tripelphosphate — ergo Typhus, — sondern in Bezug auf die möglichst detailirte Durchsicht des untersuchten Objectes und auf die Rückchlüsse, welche daraus für die Vorgänge im Organgewebe gezogen werden können. — Wenn wir nun die *„Krankheitsproducte“* nicht mit besonderem Accent hervorheben zu müssen glauben, so finden wir uns um somehr veranlasst, gegen den zweiten, darauf folgenden Satz unverholen unser Bedenken auszusprechen; denn die *Dysenterie = fibrinöses Exsudat auf der Dickdarmschleimhaut* ist unseres Dafürhaltens eben eine solche abstracte und schematische Gleichung, die auf einseitiger Auffassung beruht und eben deshalb zu Irrthümern Veranlassung gibt. Stellen wir die Gleichung als giltig hin, so folgt daraus, dass jedes fibrinöse Exsudat für Dysenterie spricht, — dass da, wo kein fibrinöses Exsudat vorkommt, keine Dysenterie ist, — wo aber eines vorkommt, dasselbe nur vom Colon herstammt — Sätze, die sammt und sonders einseitig und unrichtig sind. Viel präciser und umfassender hat Lebert die Vorgänge bei der Dysenterie dadurch bezeichnet, was er über die Excrete in den verschiedenen Graden der Ruhr angegeben: in den leichteren Formen seröse Flüssigkeit mit abgestossenem Darmepithel; in den höheren Graden Blut- und Eiterkörperchen, formlose Flüs-

sigkeit und Epithel; bei croupöser Dysenterie nebstdem fibrinöses Exsudat, in den höchsten Graden auch noch nekrotische Schleimhautpartien. Aehnlich verhält es sich bei Exsudationen der serösen Häute; das Exsudat kann als solches unmöglich anders als im *flüssigen Zustande* auf der freien Fläche erscheinen, es ist *Exsudat par excellence*, und führt im reinen Ergusse nur die fortgeschwemmten Epithelzellen des serösen Ueberzuges mit. Geht mit demselben gleichzeitig eine Zellenproduction von dem erkrankten Gewebe aus, so wird das *Serum* mit einfachen vergänglichen Elementen getrübt, und beim Ueberwiegen der letzteren über den flüssigen Bestandtheil bezeichnet man die Masse als *Eiter*. In dem einen wie in dem anderen Falle treten die gerinnfähigen Bestandtheile des Ergusses nach Massgabe ihrer Menge (häufig schon im Leben, zuweilen erst nach dem Tode) als starre Producte auf, die ungeachtet des wechselnden Aussehens von körnigen und zottigen Bildungen, faden- und leistenförmigen, netz- und membranähnlichen, einfachen und geschichteten Ausbreitungen doch nur den Namen *amorpher Gerinnungen* verdienen, da ihnen eine morphotische Bedeutung in histologischem Sinne nicht zugesprochen werden kann. Für einen gegebenen Fall mag die Diagnose durch eine a potiori gewählte Bezeichnung ihre Berechtigung haben; eine umsichtige mikroskopische Untersuchung sträubt sich aber gegen eine nominelle Diagnose ebenso sehr, als sie etwas Wichtigeres zu liefern anstrebt und zu leisten im Stande ist, nämlich ein detailirtes Gesammtbild des vorhandenen Zustandes und bei fortgesetzter Beobachtung eine complete Darstellung der Entwickelungsvorgänge und Veränderungen im Erkrankungsbezirke des Substrates.

Die Geschichte der Cholera reflectirt bezüglich der Diagnostik einen Theil der mikroskopischen Irrthümer und es bedurfte dieser, um auf dem Wege der Negation zu einer geklärten Einsicht zu gelangen, und eine Reihe von specifischen Cholera-Körperchen, welche den Horizont der pathologischen Anschauungen zu beschränken geeignet waren, nach und nach aufzugeben. Ausgehend von der Hypothese eines materiellen Agens, welches als Contagium wirkt, und welches man sich am liebsten als ein organisirtes Wesen oder geradezu als einen Parasiten dachte, stellte man an das Mikroskop keine geringere Anforderung als, den bösartigen Patho-Gnomen aufzufinden, um nachher mit einer passenden Medicin seinem Dasein ein Ende zu machen. Mit pathognomonischen Hoffnungen und

Bestrebungen hat wohl die Benützung des Mikroskops überall ihren Anfang genommen. Es bleibt mir unvergesslich, was mir in dieser Angelegenheit die hohe Autorität Purkyně's verbürgt hatte, als wir in der letzten Epidemie (1851—1854) Speisen und Getränke, Luft und Wasser und alle möglichen Stoffe auf den Nachweis der Choleraelemente hin in Angriff genommen hatten. Bei der Untersuchung des Trinkwassers aus einer verdächtigen Quelle kam eine Figur zum Vorschein, welche so sonderbar als regelmässig, so fremdartig als scharf und schön gezeichnet da lag, und die Theilnehmer der Untersuchung als das Corpus delicti — das lang gesuchte Cholerathierchen des Trinkwassers überraschte. Der Befund stand fest, er rührte sich nicht vom Objectglase, und nachdem sich alle Anwesenden von den Formen, der Grösse und der merkwürdigen Symmetrie des Wesens mit eigenen Augen überzeugt hatten, erklärte der Mikroskopiker die wahre Natur desselben — den feinen Glasritz. — Viel weiter gingen anfänglich englische Aerzte, von denen einige schön definirte runde Körper als Cholerazellen oder Pilze beschrieben hatten, die heute ein jeder als Eier von Enthelminthen erkennen müsste. — Ob die zahllosen *Vibrionen*, von denen man sich erst mit einer höheren optischen Kraft hinreichend überzeugt, im Cholera-Stuhle und Erbrochenen eine bestimmte Deutung zulassen, bleibt noch immer dahin gestellt; diese sind 0,0020—0,0040 Mm. lang und 0,0005—0,0007 Mm. breit, äusserst zart contourirt, am meisten dem *Bacterium termo* (Dujardin, Histoire naturelle des Infusoires, Paris 1841, p. 212. Tab. I. Fig. 1) ähnlich, sonst aber auch durch nichts von jenen unterschieden, denen man bei allen möglichen Zersetzungen in Flüssigkeiten begegnet. Pacini ist geneigt, denselben die Unterwühlung und Ablösung des Darmepithels zuzumuthen; in seiner Notiz (Sul Cholera asiatico, Firenze 1854, p. 11, wo die Villi intestinales als Befund der Cholera-Flüssigkeit angeführt werden), spricht er sich darüber nicht näher aus; nach einer mündlichen Mittheilung vom J. 1856 will er diese Molecular-Wesen auch im Inneren der Darmzotten, also im Bindegewebskörper der Villi intestinales beobachtet haben, und nach seiner Vorstellung wird nicht blos das Epithel von denselben unterminirt, sondern auch *das compacte Gewebe der Darmzotte derart gesprengt* und an der Basis abgesetzt, dass die Darmzotte selbst im Cholerastuhl zur Ansicht kommt.

Gewiss ist es, dass im Reiswasser-Stuhle der Cholera das Darmepithel so zusammenhängend abgelöst, und von der Schleim-

haut entfernt wird, dass nur dadurch und durch den reichlichen Wassergehalt des Ergusses das eigenthümliche Aussehen dieser Entleerung bedingt wird. Man kann zwar den diarrhoischen Stuhl durch Verdünnung mit Wasser zu einem reiswasserähnlichen machen, aber die mikroskopische Beschaffenheit der zarten weisslichen Flocken wird in diesem Falle nicht völlig mit derjenigen übereinstimmen, die bei der Cholera eine so charakteristische Desquamation in toto darstellt. Wenn es erlaubt ist, den Epithelialüberzug einiger Darmzotten mit dem Handschuh zu vergleichen, den man von den Fingern nur abstreifen kann, so ist es bei der Cholera eben immer ein ganzer Handschuh von Darmzotten (Fig. 7), den man in den Flocken der Flüssigkeit findet, ja nicht selten erstreckt sich der Zusammenhang des abgestossenen Epithels auf die ganze Circumferenz des Darmrohres und die Desquamation erscheint sodann in Form von collabirten Cylindern, deren Weite dem Caliber des Darmstückes entspricht. In dieser Weise kommt die Ablösung und Elimination des Epithels bei keiner anderen Krankheit vor. — Nach den Untersuchungen von Böhm wird auch das *Epithel der Lieberkühn'schen Drüsen* abgestossen und in Form von einfachen, blind endigenden Schläuchen oft im Zusammenhange mit dem der Darmzotten entleert. Unter anderen Verhältnissen gelingt es wohl auch, das Epithel durch Einweichen des Darms in Wasser zu entfernen, aber immer ist es in diesem Falle zähschleimig verklebt und festhaftend, während es sich im Cholera-Darm unter dem Wasser gleich einer feinpulverigen Masse erhebt und darin, oder oben aufschwimmt. Die Schleimhaut bleibt darunter vollkommen entblösst, und zeigt in diesem Falle die *sammtähnliche, feinzottige Oberfläche viel deutlicher* als bei irgend einem anderen Vorgange; denn durch den Abgang des schleimigklebrigen Epithelialbeleges, wodurch die Mucosa im normalen Zustande saftigweich und feuchtglänzend erhalten wird, treten hier die Zwischenzottenräume sehr deutlich zum Vorschein, und die mit blossem Auge wahrnehmbaren scharf markirten Zotten verleihen der ganzen Fläche ein *trockenrauhes, mehlig weissliches, mattglänzendes Aussehen.* — Die Abstossung des Epithels betrifft regelmässig die gesammte Strecke des Dünndarms; in vielen Fällen kommt sie im Magen, in selteneren Fällen auch im Dickdarm vor; die Ausfuhr jedoch ist durchaus nicht eine so constante Erscheinung, und wenn man im Cholera-Darm das Epithel-Desquamat noch an Ort und Stelle, oder an den abhängigen Partien des Dünn-

darms angehäuft findet, so ist kein Grund vorhanden, die Abstossung des Epithels für eine Leichenerscheinung zu halten, wo nur die Lähmung des Darms und der frühzeitige Tod die Ausscheidung vereitelt hatte.

Wir haben bereits oben bei der Besprechung der unvollkommenen Formen von Schleimkörperchen das Vorkommen derselben bei der profusen Cholera - Entleerung besonders hervorgehoben und die Untersuchung des Choleradarms in solchen Fällen führt zu dem Resultate, dass die Quelle dieser Formbestandtheile die geschlossenen (solitären und Peyer'schen) Follikel sind, deren Schwellung in manchen Fällen ein papulöses Exanthem, in anderen die sogenannte Psorenterie (Serres und Nonat) darstellt, in einigen Fällen durch Dehiscenz der zarten Kapsel sich entleert und als punktförmiges Grübchen markirt wird. In allen Fällen enthalten die *geschlossenen Follikel* dieselben *Elementarkörperchen, Kerne und feingranuläre Zellen,* welche den endogenen Drüseninhalt auch im normalen Zustande, jedoch nur im Verdauungsacte (wie man bei Selbstmördern, die kurz vor dem Tode gegessen hatten, findet) in namhafter Quantität, sonst aber in viel geringerer Menge, darstellen; erwiesener Massen ist die Dehiscenz derselben nicht ausschliesslich eine pathologische, sondern häufig eine blosse Leichenerscheinung, und so gilt von diesem, früher ebenso oft geläugneten, als überschätzend betonten Symptome, dass die *Intumescenz und der mammelonirte Zustand* der Drüsen an und für sich dem gesunden und dem erkrankten Darm eben so gut zukommen könne, wie die *Follicular-Dehiscenz und Reticulirung der Peyer'schen Drüsen-Plaques,* wenn man von anderen Veränderungen absieht, weder die Gesundheit der Darmschleimhaut ausschliesst, noch eine besondere Erkrankung derselben eigenthümlich charakterisirt.

Die Entblössung der Schleimhaut nach Lockerung des Epithels scheint sich zum Austritt des serösen Ergusses gerade so zu verhalten, wie die Ablösung der Epidermis und die Blosslegung der Haut zur Ausschwitzung eines beinahe wasserreinen Serums, welches sich vom Blutserum höchstens durch einen geringeren Salzgehalt unterscheidet. Nach Maassgabe der Grösse der ausschwitzenden Fläche und der Dauer des Verbleibens des Transsudates wird sich die Wechselwirkung zwischen dem Erguss und der Schleimhaut unter verschiedene Erscheinungen in Bezug auf *die Injection des Darms* vertheilen, und in Anbetracht dieser möglicherweise protrahirten Wechselwirkung befindet sich gerade der erkrankte Dünndarm in anderen Verhältnissen als

in allen übrigen Organen. In diesen imponirt die plötzlich eingedickte Blutflüssigkeit, welche beinahe gleichzeitig in allen Bezirken des Gefässsystems zum Stillstande gelangt, und die Asphyxie bedingt, an der trockenen und collabirten Leiche meistentheils als „Hyperämie", und es war nichts leichter als eine solche „Hyperämie" auch im Ganglien-System, namentlich am Plexus cardiacus anatomisch nachzuweisen, und auf die optische Erscheinung einer nicht näher auf ihre Bedingungen zurückgeführten Röthung die neuropathologische Cholera-Theorie zu gründen. Aehnlich verhält es sich mit der Unterbrechung der Harnsecretion in Folge der Eindickung des Blutes und der Beziehung der Cholera zur Urämie. Der Darm selbst kann äusserlich in eben derselben Weise bis zur gleichmässigen Rosenröthe gefärbt erscheinen, wenn in sämmtlichen Capillaren das durch den Serum-Verlust eingedickte Blut stockt: an der Schleimhaut dagegen bemerkt man die exquisiteste Anämie d. h. Farblosigkeit, zumal in jenen Fällen, wo der salzarme Erguss mit der entblössten Schleimhaut in Berührung geblieben und die geringe Quantität des Blutes in den Capillaren damit diluirt werden konnte, abgesehen von den selteneren Fällen, wo Blut in Menge ausgetreten und der Darminhalt davon blassröthlich gefärbt erscheint. — Da die Ablösung des Schleimhautepithels (und die von Pacini in einigen Fällen constatirte Trennung der Villi intestinales auf beträchtlichen Strecken des Dünndarms, woran die Schleimhaut wie glatt rasirt aussah), ohne irgend welche organische Veränderung der Gewebe auftritt, so ist sie auch hierin einer mechanischen Läsion vergleichbar und wird deshalb von dem genannten Forscher geradezu als eine traumatische bezeichnet und irgend einem organischen Wesen zugeschrieben, welches allein das unabhängige Vermögen besitzt, die Zerstörung auch nach dem Tode des befallenen Opfers fortzusetzen. Ohne gerade den gefundenen Vibrionen die Eigenthümlichkeit des Cholera-Contagiums zuzusprechen, wird nur darauf hingewiesen werden müssen, dass die Species dieser Molecular-Wesen nicht bestimmbar, ja nicht einmal ihre thierische oder pflanzliche Natur ermittelt, geschweige denn ihr biologischer Charakter erwiesen ist, und dass die Substanz des Contagiums immerhin als ein belebtes organisches Wesen, gleichviel ob organisirt oder als blastematische Flüssigkeit, gedacht werden kann (Pacini l. c.). — Die Betrachtungen, die sich an den mikroskopischen Befund anschliessen, haben wenigstens soviel für sich, dass sie mit

anderen constatirten Thatsachen nicht im Widerspruche stehen und ganz wohl mit jenen Erfahrungen vereinbar sind, welche von einem anderen Gesichtspuncte aus gewonnen wurden. (Vgl. Hauptbericht über die Cholera-Epidemie d. J. 1854 im K. Bayern im Lit. Anz. dies. Viertelj. Bd. 58 S. 16, N. 51 et seqq.).

Wenn man alle Modificationen, unter denen die Cholera sowohl in ihren Symptomen, als auch speciell in Bezug auf die Entleerungen auftritt, beachtet, so wird man nicht blos Stadien und Grade, sondern auch typische und atypische Formen — die sogenannten Anomalien — unterscheiden und es scheint, dass diese Epidemie wie alle verheerenden Weltseuchen, (wie namentlich auch der Typhus im Verlaufe der Jahrhunderte) in dem Maasse als sie endemisch wird, unter gelinderen Formen des allgemeinen Charakters solchen Abweichungen anheimfallen muss, dass diese endlich die Mehrzahl ausmachen und so sehr zur Regel gehören werden, dass dadurch unsere gegenwärtige Diagnostik und Prognostik ausser Kraft gesetzt wird. Schon bei der letzten Epidemie konnten die physikalischen Kennzeichen der Cholera-Stühle ebenso wenig von denen der diarrhoischen und dysenterischen Evacuationen getrennt werden, als der histologische Befund und die chemische Untersuchung sichere Anhaltspunkte bot. Das Individuum, die Familie, die Localität, kurz räumliche und zeitliche Verhältnisse modificiren den Typus einer jeden Epidemie mehr oder weniger, und Lindsay hat (im Association Journal October 1854) hinreichend hervorgehoben, wie sehr die Cholera bei Geisteskranken modificirt werden könne. Die Veränderungen an der Schleimhaut gestatten wohl im Allgemeinen die Annahme, dass die schädliche Potenz einmal rapid einwirkt und sich auf einem bestimmten Puncte concentrirt, so dass die Läsion sehr intensiv erscheint, während ein andermal die Veränderungen mehr extensiv und zugleich auch mehr oberflächlich auftreten; doch erhalten dadurch die Unterschiede einer Cholerine und der wahren Cholera, die der sporadischen und der epidemischen u. s. w. keine so feste anatomische Basis, um die ungefähre Abschätzung der Erscheinungen auf ein bestimmtes Maass zurückführen zu können. Beachtenswerth ist die Bemerkung von Lindsay, dass die Darmentleerungen der Geisteskranken sich im Allgemeinen durch ihre schleimige Beschaffenheit bei den verschiedenen Affectionen der Darmschleimhaut besonders auszeichnen, wie dies sonst nicht der Fall ist; da dieser Autor hierunter die Bevölkerung einer Irrenanstalt versteht und dieselbe mit der eines Kranken-

hauses vergleicht, so sollte die Dichtigkeit von Bewohnern gewisser Anstalten überhaupt berücksichtigt werden, um die Eigenthümlichkeiten der herrschenden Krankheit unter diesen Bedingungen (Casernen, Strafanstalten etc.) hervorheben zu können. Die strumöse Diathese könnte die Häufigkeit der schleimigen Entleerungen eben nicht erklären, so lange sie, so wie die skrophulöse Disposition, nicht durch bestimmte Veränderungen der Gewebe oder sonstige Erscheinungen bezeichnet ist. Struma und Rachitis, Skrophulose und Tuberculose haben allerdings zum gemeinschaftlichen Ausgangspuncte das Drüsensystem im weitesten Sinne des Wortes, wobei das *periphere Lymphsystem* in seiner unmittelbaren Beziehung zum *Stützgewebe des Organismus* (den Bindegewebskörperchen, welche Leydig als die Anfänge der Lymphcapillaren erwiesen hat) und der *Darm als erste Quelle der Hämatopoëse* die hervorragendste Rolle spielen. Allein der histologische Charakter der Organe, das Eigenthümliche, was die „Disposition" macht, ist noch so sehr in Dunkel gehüllt, wie die ursprünglichen (die letzten materiellen) Bedingungen, worauf endlich jede Reihe von wahrnehmbaren Veränderungen zurückzuführen ist.

Typhus. Die profusen Darmentleerungen haben in der Summe ihrer Kennzeichen allerdings viel Charakteristisches, wodurch sie sich von einer serösen Diarrhöe, von Cholera und von Dickdarmkatarrh unterscheiden. Im Vordergrunde steht hier die *Epithel-Desquamation*, welche jedoch nicht in grossen zusammenhängenden Flächen, sondern in kleinen losen Partikelchen auftritt. Diese betrifft vor Allem und vorzugsweise die Schleimhautflächen der durch den Process infiltrirten Drüsenhaufen (Peyer'schen Follikel) und ist von reichlichem formlosen Erguss in das Darmrohr begleitet, in welchem das Epithel alsbald durch Maceration derart zersetzt und misstaltet wird, dass es in der dünnbreiigen Fäcalmasse nur noch in Trümmern erkannt wird. Die resistenteren Kerne der Epithelzellen erscheinen unter dem Mikroskope als unzählige, gelbgefärbte Körperchen, denen häufig zarte, wolkige Reste der zerstörten Zelle anhaften; die gelbe Farbe rührt allem Anscheine nach von galliger Imbibition her, die in dem moleculären Detritus aus der zerfallenen Zellensubstanz nur undeutlich in die Erscheinung tritt. — Da die chemische Umsetzung der abgestossenen Massen und der vorhandenen Ingesta hiebei in rascher Weise und in grossem Maassstab vor sich geht, so kommt es bei Gegenwart von kohlensauerem Ammoniak und Erdphos-

phaten zur Bildung von *Krystallen in grosser Menge*, die nach Ausscheidung der Kohlensäure Verbindungen der Phosphorsäure mit Magnesia, Ammoniak (und Kalk) darstellen und in allen Formen des prismatischen Systems beobachtet werden. Man hat anfänglich auf die Sarg-Deckel-Formen der Trippel-Phosphate zu viel Gewicht gelegt, und später denselben allen diagnostischen Werth rein abgesprochen; der Mittelweg in der Abschätzung ist der beste. Es ist gewiss, dass die Phosphate nicht blos bei allen möglichen Stuhlentleerungen der verschiedensten Krankheiten und selbst in Excrementen gesunder Menschen gefunden werden, da sie nur das Product eines rein chemischen Vorganges in thierischen Substanzen darstellen, und somit auch in anderen Excreten, namentlich in Harnsedimenten, nicht fehlen werden; allein die relative Menge derselben in einem jeden mikroskopischen Bilde, und die absolute Quantität der innerhalb einer kurzen Frist abgegangenen Salze, welche Simon in der Asche auf 30—32% (darunter 12—13% phosphorsauerer Magnesia) angibt, bezeichnen die typhöse Stuhlentleerung in Verbindung mit den übrigen Merkmalen des Substanzverlustes doch unverkennbar. Funke hat auch *kohlensauren Kalk* in Form krystallinischer Massen und trommelschlägel-ähnlichen Figuren im typhösen Stuhle gefunden. — *Blut* erscheint im typhösen Darmexcret viel constanter als bei irgend einem pathologischen Process und zwar von mikroskopischen Quantitäten bis zu einer mit dem blossen Auge wahrnehmbaren Menge. Die Hyperämie und Infiltration der Follikel führt schon häufig zu capillarem Blutaustritt; die Dehiscenz und Ulceration derselben ist umsomehr von Blutabgang in beträchtlicher Masse begleitet. Von Substanzverlusten der Schleimhaut, die bei den verschiedenen Krankheiten vermuthet oder erst durch die Autopsie nachgewiesen werden, zeichnet sich das typhöse Geschwür durch Blutungen am meisten aus; indessen ist der „blutige Stuhl" nach einer mit blossem Auge gemachten Wahrnehmung durchaus kein sicherer Anhaltspunkt, da das Blut in den Faecalmassen von Gallenpigment und Ingestis viel mehr verdeckt sein kann als z. B. im Harn bei Haematurie, und das Mikroskop in solchen Excreten die Blutzellen viel genauer nachzuweisen im Stande ist, als der geübteste praktische Blick. Die grosse Quantität des abgegangenen Blutes ist für sich betrachtet ebenso wenig charakteristisch beim Typhus, als sie bekanntlich auch anderen Zuständen zukommt, nämlich der Dysenterie, der Verletzung gros-

ser Gefässe, der Leukämie und in exorbitantem Maasse der allgemeinen Anämie, die in capillaren Blutungen auf ausgedehnten Schleimhautstrecken eben sowohl ihre Quelle als ihren lethalen Abschluss findet. Viel wichtiger als das Quantum, scheint uns die Beschaffenheit der Veränderungen der Blutzellen im typhösen Darminhalt zu sein, deren Erscheinungen wohl der Hauptsache nach auf die Dauer des Aufenthaltes der Blutzellen im Darmrohre, auf die durch denselben zurückgelegte Strecke und auf den sonstigen coëxistirenden Darminhalt zu beziehen sind. Auffallend ist es, dass man an einer Anzahl von Blutkörperchen unter sonst ähnlichen Verhältnissen die verschiedensten Umwandlungen zu Gesichte bekommt: aufgeblähte, vollkommen helle Bläschen mit geringer oder gar keiner Färbung, zarte Hüllen ohne Inhalt, geschrumpfte und eckige hellgelbe Körperchen, Stern- und Biscuitformen von rothbrauner Farbe, verzerrte Figuren, worunter die backschüssel-förmigen die häufigsten sind, endlich aus den vorigen abzuleitende desaggregirte Pigmentkörnchen. Einestheils gibt wohl der Contact der ausgetretenen Blutflüssigkeit mit den verschiedenen Massen, dem Exsudate in den Follikeln, dem salzhaltigen Serum, den Nahrungsmitteln und Medicamenten, Anhaltspunkte genug zur Verfolgung der variablen Erscheinungen; anderentheils drängt sich jedoch hier wie bei allen Experimenten mit dem Blute der Gedanke unabweichlich auf, dass die einzelnen in einer Gruppe beisammen stehenden Blutkörperchen auch ursprünglich in ihrer Grösse, Form und inneren Constitution einigermassen abweichen und zwar mehr als es blos die Altersdifferenzen derselben beurkunden sollten.

Der *Eiweissgehalt* der typhösen Stühle ist grösser als man beim Aufkochen der filtrirten Flüssigkeit anzunehmen hätte, denn das kohlensauere Ammoniak hält das Eiweiss auch beim Erhitzen gelöst und man erhält erst nach Zusatz einer Säure das Coagulum. Nicht blos der Erguss in den Follikel-Apparat, sondern auch der Austritt des Blutes en masse ist als Quelle dieses Proteinstoffes zu betrachten, dessen Qualitäten man auch gemeinhin auf das Infiltrat bezieht, da die Chemie hierin keine genaueren Unterschiede festzusetzen vermag.

Untersucht man die Schleimhaut eines zur Autopsie gekommenen Darms, so findet man, dass das Infiltrat der noch nicht dehiscirten Follikel neben dem amorphen Erguss eine massenhafte *Zellenproduction* (Fig. 6, *e*, *f*.) enthält, die sich häu-

fig durch excessive Grösse der sphärischen Elemente und grosse, ein- und mehrfache Zellenkerne auszeichnet. Wenn schon der blosse Blick das Infiltrat der beträchtlich geschwollenen Schleimhaut markähnlich findet und das unbewaffnete Auge ein solches Product mit dem des Medullar-Krebses vergleicht, so weist das Mikroskop bei der Leichenuntersuchung diese Aehnlichkeit bis ins Detail nach und es bleibt histologisch nur der indirecte Beweis einer Verschiedenheit der Zellenmassen übrig; die differentielle Diagnose beschränkt sich hier allein auf die Angabe des Mangels eines Krebsgerüstes und der dem Krebs unter gewöhnlichen Verhältnissen zukommenden Metamorphosen der Elemente seines rahmähnlichen Saftes. Allerdings gibt der Sitz, die Ausbreitung und der klinische Verlauf anderweitige Anhaltspunkte, welche in Uebereinstimmung mit dem erwähnten mikroskopischen Befund die Diagnose sicherstellen. — Schwieriger als an der Leiche wäre die Diagnose am Krankenbette auf die dem typhösen Infiltrat zukommende Zellenmasse zu basiren; denn äusserst selten gelingt es, den durch Follicular-Dehiscenz und Ulceration abgestossenen Zelleninhalt in so unverändertem Zustande nachzuweisen, dass ein untrüglicher Rückschluss aus diesem Befunde allein zu ziehen wäre. Dieses Gewebe ist nämlich ungemein vergänglich und ebenso rasch als es entstanden, zerfällt es auch. Die mehrkernigen Zellen zumal schwinden oft während der Untersuchung unter dem Mikroskope, ihre zarten Hüllen lösen sich in einen wolkigen Detritus auf, welcher die mehr widerstandfähigen rundlichen und eckigen *Kerne* zu dunklen sphärischen Molecularmassen umhüllt und dieselben im Darminhalt als *weissliche Pünktchen* und *Körnchen* erscheinen lässt. — Von gleicher Bedeutung mit diesen sind die mehr oder minder zahlreichen, deutlich als solche wahrnehmbaren, *farblosen Zellen*, die sphärisch, ungleich klar, zuweilen glatt oder etwas rauh und matt granulirt erscheinen, bei Behandlung mit schwach angesäuertem destillirtem Wasser ihren kleinen Kern oder mehrere derselben zu erkennen geben und sich somit als einfache (Schleim- oder Eiter-) Zellen erweisen, wie sie auf der Schleimhaut als auch in den Follikeln vorkommen. Man kann sie wohl als jüngere Formen der oben beschriebenen grossen Zellen des Drüsen-Infiltrates ansehen; die Entwickelungsstadien geben sich eben durch ein Grössenmaass kund, welches zugleich den Grad ihrer Vergänglichkeit ausdrückt, so dass die grössten und ältesten am wenigsten resistiren

und im Typhusstuhle sehr selten zur Beobachtung kommen, während die kleinsten, jüngsten Elemente beinahe unverändert entleert werden. — Bei länger angehaltenem Stuhle, wo ein einigermassen weniger wässriges, mehr breiiges Secret entleert wird, findet man in den unteren sich absetzenden Schichten zuweilen beträchtliche Klümpchen dieser Kerne- und Zellenhaufen, die von feinmoleculärem Detritus zusammen geballt und von Erdphosphaten niedergehalten werden. Man gewinnt in einem solchen Falle auch die Ueberzeugung, dass die Krystallisation der Trippelphosphate viel massenhafter auftritt als in einem mehr wässerigen Stuhle, und dass die Menge der vorgefundenen Krystalle mithin einen ungefähren Maassstab für die Dauer der Stagnation der Darmcontenta abzugeben geeignet ist.

Bei der Schorfbildung an typhösen Geschwüren kommt endlich bei sorgfältiger Untersuchung *nekrotisches Schleimhautgewebe* zur Beobachtung, dessen Nachweis den Zustand hinreichend charakterisirt, in welchem sich die erkrankte Darmpartie befindet. Das nekrotische Gewebe gibt sich hier, so wie aller Orten, in mikroskopischen Partikelchen durch einen zäheren Zusammenhang der übrigens durch Maceration zu einem morschen, schmutzig braunen Flocken erweichten Gewebselemente zu erkennen; unzweideutig ist jedesmal die fest- und zähfaserige Spaltbarkeit, welche ein solcher Gewebsfetzen beim Ausbreiten auf einem Deckglase zeigt, und welche sonst keinem anderen wie immer gearteten Exsudate auf der Schleimhautfläche zukommt. Die Faserung lässt kaum je eine weitere Deutung des Befundes auf ein specielles Gewebe zu; indessen genügt es, sich gegenwärtig zu halten, dass dieses Bild nur in jenen seltenen Fällen erscheinen wird, wo nach Exulceration der Follikel das Reticulum, welches die Schleimhaut als Drüsenskelett in den Peyer'schen Plaques bildet, durch Schmelzung und Maceration zerfällt und abgestossen wird. Ich habe die Nekrose der Darmschleimhaut bei Typhus in einem Bilde zur Beobachtung bekommen, welches dem der Nekrose der Laryngeal-Schleimhaut — namentlich der Epiglottis — vollkommen ähnlich war; an der Epiglottis, wo meines Wissens die typhöse Zerstörung nicht beschrieben wird, kamen im Verlaufe des J. 1857 drei Fälle im k. k. Garnisonsspital vor, worunter einer mit brandigem Zerfall des Netzknorpels und Schwund desselben bis auf den fünften Theil der ganzen Länge des Kehldeckels. Hier gibt das resistente Fasernetz nach vor-

ausgegangener Zerstörung der darin eingeschlossenen Knorpelzellen einen sicheren Anhaltspunkt zur Bezeichnung der Localität, welche den Sitz der Zerstörung abgibt; in allen Fällen jedoch findet man an dem faserig ausgezogenen Gewebsrudimente rauhe zackige Contouren der gelösten Textur, Trübung derselben durch feine Molecularmassen und Pigmentkörnchen, die an den Rändern der Fäserchen innig haften, und gleichmässige Durchfärbung mit einer trüben Flüssigkeit, deren Entfernung nur durch wiederholtes Auswaschen gelingt. Ist das Präparat möglichst rein gewaschen, was am Besten unter beständiger Controle des Mikroskopes bei einer schwachen Vergrösserung auszuführen ist, so gewahrt man in den meisten Fällen eines wirklichen Gewebes die zarten spindelförmigen Kerne des theils zusammenhängenden, theils aufgefaserten Bindegewebes, nicht selten auch kurze Fragmente abgerissener elastischer Fäserchen. — Gewinnt man mit dem Gewebsflocken auch entblösste, jedoch unversehrte Darmzotten, so gewahrt man in denselben *reichliche, rothbraune Pigmentkörnchen*, wie es scheint, vorzugsweise den Kernen oder Bindegewebskörperchen entsprechend angeordnet. Dies kommt übrigens auch bei Cholera vor, worauf Wedl zuerst aufmerksam gemacht hat.

Diese Veränderung der Schleimhaut ist regelmässig von profuser Blutung begleitet, welche sich jedoch ebenso häufig der blossen Wahrnehmung entzieht, als sie auch mikroskopisch nicht direct nachweisbar, ja selbst an der Leiche nur unter Berücksichtigung aller Umstände erschlossen werden kann. Das Blut befindet sich nämlich in solchen Fällen in einem durchaus dissoluten Zustande und gleichwie es an der Schleimhautfläche diffuse Imbibitions-Flecke im Bereiche der typhös erkrankten Darmpartie bedingt, ebenso verleiht es den Contentis und dem Excrete zuweilen eine tiefere röthlichbraune oder grünlich graue Nuance der Farbe, in welcher kaum der Antheil der galligen und der Blutfarbstoffe nachzuweisen sein wird. Im Darmcanal markirt sich der Blutfarbstoff als dunklere Pigmentirung, welche im Bereiche der blutigen Durchtränkung der Schleimhaut später in verschiedenen Stufen schiefergrau erscheint. — Treibt man während der Untersuchung nekrotischer Schleimhautpartien aus den Gefässen der Nachbarschaft Blut in den Geschwürsgrund hinein, um es der Untersuchung zu unterziehen, so gewahrt man in der Flüssigkeit zuweilen ganz ausgezeichnet die Erscheinun-

gen der *Blutdissolution* und *Sepsis*, wie sie Rokitansky beschrieben und (S. 386 Pathol. Anat. I. Bd. 1855) gezeichnet hat. Uebrigens gilt für die Darmblutung sowohl bei Typhus als auch bei allen übrigen Krankheiten die Regel, dass man die Quelle derselben am allerwenigsten dort suchen dürfe, wo die Blutspuren an der Leiche gefunden werden. Die Gewebsimbibition bezeichnet wohl in vielen Fällen die Strecke der krankhaften Läsion, das Blut en masse befindet sich in einem mehr oder weniger alterirten Zustande oft sehr weit entfernt auf einer tiefer liegenden Schleimhautstrecke, die von Schleim und Epithel geschützt und unverändert aussieht (Fig. 6, *a*).

So wie bei Nekrose der Darmschleimhaut auf losgetrennte Gewebsportionen ganz besonders Rücksicht zu nehmen wäre, so hat man sich wieder in Acht zu nehmen, gewebsähnliche Flocken von anderen Gegenständen, namentlich von *geronnener Fibrine* damit zu verwechseln. Es kommt dem Exudationsprocess überhaupt und dem Blutaustritt insbesondere zu, dass die gerinnfähigen Bestandtheile der Flüssigkeit erstarren und in mikroskopischen Fäserchen, membranähnlichen Ausbreitungen und netzförmig durchbrochenen Täfelchen zur Untersuchung kommen, welche den Fragmenten einer zerstörten Schleimhautstelle oft täuschend ähnlich sehen, dieselbe gelbliche Färbung zeigen wie jene, und zuweilen selbst zellige und kernartige Gebilde — vielleicht Elemente der Exsudatzellen oder Reste der geformten Blutbestandtheile einschliessen. Ihre Elasticität beim angewandten Zuge oder beim Drucke unter dem Deckgläschen, ihr starkes jedoch ungleichförmiges Lichtbrechungsvermögen und das Aufquellen, dem sie bei Zusatz eines kleinen Tröpfchens von Essigsäure unterliegen, lässt sie bald als amorphe Gerinnungen erkennen.

Die *Ingesta* der typhösen Stühle, welche sich binnen 24 Stunden oft wiederholt hatten, tragen an jedem folgenden Stuhle immer mehr die Zeichen der Unversehrtheit und somit der geringen Einwirkung der Darmsäfte auf dieselben, so dass man in einem solchen Bilde unveränderter Muskelfaser-Fragmente, Amylon-Körperchen, Pflanzen-Resten (besonders nach Genuss von Citronensaft) den Ausdruck der darnieder liegenden Verdauung findet. Zugleich bemerkt man, wie bei profusen Darmentleerungen überhaupt, auch beim Typhus zuweilen den reichlichen Abgang von *Eiern der Enthelminthen* (Fig. 11): Ascaris, Oxyuris und Trichocephalus, welcher letztere bei der Autopsie nicht selten in beträchtlicher Anzahl unmittelbar hinter der

Bauhin'schen Klappe, im Coecum und im Colon ascendens vorfindlich ist.

Es bedarf nicht erst einer besonderen ausführlichen Discussion darüber, dass der mikroskopische Befund des typhösen Stuhles je nach dem Zeitpunkte variiren muss, wann das Secret im Verlaufe der Krankheit einer genauen Untersuchung unterbreitet wird, ja es wird sehr viele lethal ablaufende Typhusfälle geben, wo nicht eine einzige Prüfung am Krankenbette das angestrebte Ergebniss liefert. Es verhält sich hier wie mit allen Erkrankungen, wo nur die Summe aller wahrnehmbaren Erscheinungen zu einem Schlusse berechtigt, und das Mikroskop kann eben nichts mehr leisten, als eine dieser Erscheinungen analysiren, um ihre messbaren Grössen näher zu bestimmen und die hervortretenden Einzelnheiten auf ihre Bedingungen zurückzuführen. Wenn nun in einem Falle gerade diese Erscheinung keinen Aufschluss gibt, so ist der Ausspruch des Mikroskopes hierüber trotz seiner negativen Bedeutung beinahe eben soviel werth als der beste positive, indem der erste zum weiteren Verfolg der ungewöhnlichen Bedingungen auffordert. Folgender Fall mag in dieser Beziehung für viele andere gelten.

J. R., 21 Jahre alter Gensd'arme, starb an Typhus mit Milztumor am 27. April 1858 nach sechstägigem Krankenlager. — Im Dünndarm galligtingirter Schleim mit Epithelialdesquamation, die Schleimhaut gallig imbibirt, nur im unteren Ileum in einer Strecke von sechs Zoll gleichmässig dunkelroth gefärbt, jedoch ohne Spur einer deutlichen Gefässinjection; der Dickdarm in seiner ganzen Capacität mit dickbreiigen, festhaftenden Fäcalmassen vollgepfropft, die Flexura sigmoides und das Rectum besonders stark erfüllt, und der Beckenraum hiedurch wie ausgestopft. Mesenterialdrüsen linsengross, dicht, fest, farblos; im untersten Ileum Infiltration der Follikel auf folgende Stellen beschränkt: auf eine Strecke von 4 Zoll vor der Cöcalklappe einzelne solitäre Drüschen zu hirsekorngrossen Knötchen entwickelt, an vier Gruppen eine Schwellung bis zur Höhe einer halben Erbse wahrnehmbar, an einer nebstdem die Dehiscenz in Form einer centralen linsenförmigen Vertiefung wahrnehmbar, aus welcher sich ein rahmähnlicher Saft ergiesst (dieser Aufbruch scheint erst als Leichensymptom aufgetreten zu sein). Von den Peyer'schen Drüsenhaufen nur *fünf Plaques* im Zustande einer 1 — 1½ Lin. hohen Schwellung und *markähnlichen Infiltration*, einer davon zur Hälfte, zwei andere, u. z. die von der Cöcalklappe entferntesten, in ihrer ganzen Ausdehnung reticulirt, das *Reticulum breiig weich* und zu einer morschen Pulpa zerfliessend, schmutzig durchröthet, die Umgebung dunkelroth imbibirt. Die zuführenden und abgehenden Mesenterialgefässe beinahe leer, der Darminhalt in dieser Strecke blutroth durchfärbt. — Aus dem übrigen Leichenbefund wird es genügen, Folgendes hervorzuheben: Blutaustritt in den subcutanen Fettpolster des Hypogastriums, zwischen Muskelfascien daselbst, in die Musculatur selbst

und besonders massenhaft auf einer bis 1 Lin. dicken Schichte und darüber in das subperitoneale Bindegewebe von der Symphyse bis an den Nabel seitlich bis in die Lendengegend; blutige Durchtränkung der beiden Lungen bei gleichmässigem Luftgehalt in sämmtlichen Partien, mit Ausnahme der luftarmen Partien des hinteren Umfangs der unteren Lappen; endlich Anstauung reichlichen, dick- und zähflüssigen, dunkelrothen Blutes in den kleineren Venen der Hirnhäute und ähnliche dichtgesäete Blutpunkte an den Schnittflächen des Gehirns. — Die mikroskopische Analyse der reichlichen Dickdarmfaeces ergibt (wie in Fig. 1) gelblichen Detritus in vorwaltender Menge, reichliche Emulsion und humusartige Substanzen in granulären Kugeln von 0.030 — 0.070 Mm., hellgelbe, scharfcontourirte Plaques von Gallenbestandtheilen, von ähnlicher Grösse und variablen Formen, zum Theil vollkommen glatt, zum Theil von Oeltröpfchen getupft; kurze starre, dunkelrandige Trümmer von Pflanzenfasern und Fragmente von Spiralgefässen (reichlich); Fetttropfen und prismatische Krystalle (untergeordnet).

Der Befund bei Darm-Tuberculose entspricht in den meisten Fällen dem des einfachen Katarrhs, mit Ausnahme jener Fälle, wo auch dieser fehlt und der pathologische Vorgang sich jeder sicheren Diagnose, ja selbst oft der Vermuthung entzieht. Dies gilt von den Fällen des secundären Auftretens der Tuberculose im Darm ebenso wie von den ungleich selteneren, jedoch nicht zu läugnenden Fällen, wo die tuberculöse Destruction den Darm allein, oder wenigstens primär betrifft, wobei dann ein acuter Nachschub von Miliartuberculose in den Lungen dem Leben ein Ende setzt. Die Blutungen sind bei tuberculöser Phthise, nach dem Befund zu schliessen, wie erwähnt, ganz unerheblich und man findet selbst lebhaft injicirte Randgefässe an den Geschwüren derart in ein derbes Infiltrat eingebettet, dass sie zum Durchbruch weniger disponiren; bei tiefgreifender Destruction entzieht sich aber selbst eine namhafte Blutmenge durch die bei längerem Aufenthalt im Darm bedingten Alterationen der approximativen Bestimmung. Wenn daher die Darmblutungen nicht fehlen, so sind sie doch imperceptibel und ihre Bedeutung um so höher anzuschlagen, als die Oekonomie des Organismus durch diesen Substanzverlust auf ungeahnte Weise Schaden leiden muss. — In einigen seltenen Fällen bemerkte ich an tuberculösen, durch organisirte Adhäsionen fixirten Geschwüren den durch Nekrose sämmtlicher Darmschichten ermöglichten *Prolapsus* von ähnlich erkrankten *Mesenterialdrüsen* (an der Coecalklappe) nach dem Darmrohre hin; (eine ähnliche Erscheinung kam im Kinderspitale an der Trachea tuberculöser Kinder in den letzten zwei Jahren dreimal vor), und es wäre in ähnlichen Fällen an die Möglichkeit zu denken, in den Auswurfstoffen nekrotisches Drüsen-

gewebe nachzuweisen. Dass nekrotisches Gewebe in den Sputis, worauf man bei Phthisikern zu fahnden pflegt, das Periculum in mora signalisire, ist jetzt allgemein bekannt; nur möge man in einem solchen Falle den Befund nicht voreilig für den Inhalt von Cavernen erklären, welche vom starren Callus umgeben äusserst selten durch den Respirationsact entleert werden können. Alle sonstigen Möglichkeiten vertheilen sich aber auf den gesammten Tractus der ersten Wege, von wo aus die Elimination nekrotischer Gewebsrudimente viel leichter erfolgt.

Eiter in Klumpen oder in Form zäher grünlich-gelber Massen wird zuweilen bei *Follicular-Verschwärung* entleert, wenn die Geschwüre tief und weit verzweigt sind, um den Inhalt unter dem Niveau der Schleimhautfläche zurückzuhalten. In solchen Fällen (Fig. 4) kommen nebst den *Humusklümpchen* (*b*), dem erdigen Detritus (*c*) und den langen in lebhafter Bewegung begriffenen *Vibrionen* (*d*) als Beimengung der Eitermasse (*a*) zuweilen die ungewöhnlichsten Gegenstände zum Vorschein, die je die Darmwege passirten und in den Lücken stecken blieben: alle möglichen Sämereien, Fragmente von harten Schalen des Kernobstes, verholzte Pflanzenzellen, Splitter von metallenen Kunstgegenständen, Chitindecken von essbaren und unessbaren Thieren; nicht selten führen diese Gegenstände bei der Exulceration der Darmschleimhaut die submucöse Fistelbildung weiter fort und Fälle von Perforation des Dickdarms (meistentheils des Coecum) in Folge von Anschoppung fremder Körper sind häufig genug beobachtet worden. — Welchen unglaublichen Ausgang eine Darmperforation bei chronischem Bestande nehmen und welche *Neubildungsvorgänge* sie zu veranlassen im Stande sei, lehrt folgender Fall:

Pietro Oberti. 24 J. alt, Lombarde vom 43. Inf. Regt., starb am 14. Jänner 1858 an *chronischer Peritonitis.* — Der Sectionsbefund erwies neben chronischer Bronchitis mit Lungenemphysem und Infarct, Lungenödem in beiden unteren Lappen, chronische Meningeal-Trübungen und Bindegewebsverdickungen mit Phlebektasie und serösem Erguss, Osteophyt an der Lamina interna; die Nieren waren, entsprechend dem marastischen Zustande fettig und colloid entartet; die Bauchdecken waren eingezogen, teigig weich; das Peritonaeum zeigte die näher zu besprechenden Veränderungen, deren histologische Untersuchung von besonderem Interesse war. Die Baucheingeweide unter einander und mit den Bauchwandungen durch dichte, schiefergrau gefärbte Bindegewebsmassen fest verwachsen und allseitig fixirt; die kaum entwirrbaren Darmwindungen im Bereiche des Beckens und am Hilus hepatis zahlreiche, zum Theil isolirte, zum Theil durch Fistelgänge unter einander communicirende Abscesse einschliessend; der Inhalt der letzteren dicker, grünlich gelber Eiter. Bei dem Versuche die einzelnen Partien des Darmrohres zu trennen, wichen die Schichten der

Darmwandungen, namentlich die Mucosa und die Muscularis viel leichter aus einander, als die Verwachsungen des Peritonaeum. Die Schleimhaut des Magens und des übrigen Gedärms schiefergrau durchfärbt, verdickt, leicht abstreifbar, besonders im unteren Ileum und im ganzen Colon ungemein brüchig und ihre Schichten von den tieferen Lagen sehr leicht trennbar. In den Gefässen nur spärliches dünnflüssiges Blut. — Darminhalt: breiig-erdige, wenig gefärbte Fäcalmassen, gleichmässig über die Schleimhaut-Flächen verbreitet und wie hingeschmiert. *An zwei Stellen des Darmrohres Perforation der Wandung und Communication mit dem Bauchfellsack*, u. z. unmittelbar an der Bauhin'schen Klappe in Form eines unregelmässig ausgezackten, die Cöcalklappe selbst betreffenden Substanzverlustes, und dann an der Flexura lienalis coli in Form einer etwa 10 Linien langen Querspalte des Rohres. Von der ersten Stelle aus ziehen sich ungleich weite Hohlgänge, zum Theil innerhalb der bindegewebigen Adhäsionen, zum Theil längs des Darmrohres, nach oben gegen den unteren Umfang der Leber und nach unten in den Beckenraum hin, woselbst sie in einen geräumigen, die hintere Blasenwand und den ganzen Douglas'schen Raum einnehmenden Blindsack münden. Von der Flexura coli lienalis aus verfolgt man in ähnlicher Weise tief- und weitverzweigte Rinnen, deren unterste an der oberen Krümmung des S Romanum ihre Gränze findet, ohne mit der vorhin erwähnten im Beckenraume zu communiciren. *Sämmtliche Räumlichkeiten dieser abnormen Rinnen und Schläuche sind mit Fäcalmassen* dicht erfüllt, welche dieselbe breiig-erdige, blassgelbe Beschaffenheit haben wie der Darminhalt. Nach sorgfältiger Entfernung des Kothextravasates und Abspülung der getrennten Darmwindungen und der zwischen denselben eingebetteten Eiterdepôts kam ein überraschendes Bild zur Ansicht: *sämmtliche Wandungen* dieser Hohlräume hatten ein sammtähnlich villöses Aussehen und waren *von dicht vascularisirten Zotten bedeckt*, deren volle Injection der ganzen Fläche eine lebhafte Röthung verlieh und deren Länge sich nach Aufgiessen frischen Wassers, woselbst sie frei flottiren konnten, auf 2—5 Millimeter bemessen liess. — Die Abscesswandungen waren hingegen von glattem, blassgrau gefärbtem Callusgewebe gebildet und ohne namhafte Vascularisation.

Die glatten Schleimhautränder an den beiden Durchbruchstellen, der umfängliche Substanzverlust an dem Mesenterialüberzuge daselbst, sowie die durchgreifende Verwachsung des Darms mit Einschluss von Abscesshöhlen, in deren Umfang die anliegenden Darmwandungen von der serösen Fläche aus verdünnt erschienen, setzten bei dem Mangel von Geschwürsbildung an der Darmschleimhaut die Entstehung der Perforation durch die Peritonitis ausser allen Zweifel. Interessanter als dies ist die Thatsache des wochen- vielleicht monatelangen Bestandes einer mit der Existenz noch verträglichen Kothaustretung, deren enorme Ausbreitung wohl ihres Gleichen sucht. Die *Organisation* der von dem ausgetretenen Kothe *neugebahnten Wege* liefert nicht blos den histologischen Nachweis der Möglichkeit eines langwierigen Contactes fremder, anorganischer Massen mit serösen Häuten, sondern auch wichtige Anhaltspuncte über den Vorgang *der Neubildung einer schleimhautähnlichen Textur*, welche wenigstens theilweise die Function der normalen Schleimhaut zu versehen geeignet ist. Die im frischen Zustande vorgenommene Untersuchung der schleimhautähnlichen Auskleidung (Fig. 10.) betraf zunächst die Villositäten an der

hinteren Blasenwand, wo sie in exquisitester Länge und Vascularisation zu finden waren. Von dem ins Wasser gelegten Hautstück liessen sich durch Scheerenschnitte einzelne Zöttchen und Gruppen derselben abtragen, welche bei einer variablen Länge von 2—5 Mm. eine Dicke von 0.5 — 0.8 Mm. hatten; zwischen denselben lagerten zahlreiche kleinere Cylinder und kolbenförmige Gebilde von 0.2 -- 0.8 Mm. Länge und 0.2 -- 0.3 Mm. Breite, welche der Fläche an einzelnen Stellen ein feinkörniges oder weichfilziges Aussehen verliehen. Die Zöttchen waren sämmtlich von colossalen Capillargefässen durchzogen, deren Caliber an den messbaren Stellen zwischen 0.020 — 0.040 Mm. schwankten. In den wenigsten Zotten fand sich eine einzige Gefässschlinge vor, an der das zu- und das abführende Gefässstück nur in der Basis kenntlich, an der Spitze hingegen die Schlinge sackförmig ausgedehnt erschien; an den breiten Zotten drängte sich ein ganzes Convolut von Gefässwindungen vor, deren Schlingen hie und da in einigem Abstand von einander entfernt die netzförmige Vertheilung der Blutbahn — eine Art Rete mirabile — sehen liessen, anderwärts dagegen zu einem Knäuel zusammengeballt, die Form der Malpighischen Körper in der Niere nachahmten. In der Tiefe communicirten die einzelnen an die Zöttchen hintretenden Gefässe in einer deutlich wahrnehmbaren Netzform untereinander; die Maschen dieser letzteren bald rundlich und polygonal, bald gestreckt, überall durch zahlreiche Gefässverbindungen mit einem tiefer liegenden, ähnlich geformten Gefässnetz in Communication stehend. An dem tieferen Gefässnetze zeigten die einzelnen Abschnitte wohl ein grösseres Caliber als das vorhin erwähnte der Endschlingen; das oberflächliche Gefässnetz kam jedoch mit den letzteren so ziemlich überein, so das es vielmehr schien, als wären die peripheren Endschlingen geräumiger als die zuführenden Communicationswege an der Basis der Zöttchen, eine Erscheinung, welche sich an blutstrotzenden Gefässen neuer Bildung häufig wiederholt.

Da sich die weiche und succulente Neubildung sehr leicht trennen liess, so gelang es nicht schwer, das Bindegewebsstratum der Serosa als diejenige Ausbreitung zu erkennen, von welcher aus nach Schwund der oberflächlichen Schichte und mit Fortsetzung der praeexistenten Gefässe die ganze weichfilzige Ausbreitung der neuen Haut sich entwickelt hatte. Nach Abnahme dieser letzteren an einzelnen Stellen präsentirte sich das fibröse Gewebe des Peritonaeums rauh, aufgelockert und hie und da netzförmig aufgefasert, allenthalben von blutführenden Gefässen reichlich durchzogen. — Die mikroskopischen Bilder der abgetragenen Neubildung variirten je nach der Mächtigkeit der Auskleidung; am meisten erschien das Bild charakteristisch, welches der dünnsten Lage entnommen in Fig. 10 bei einer 80mal. Vergrösserung (Amici) wiedergegeben ist. Aus einer reichlich vascularisirten Grundlage *A* erheben sich am Rande des Scheerenschnittchens zahlreiche ungleich grosse Villositäten *B*, deren jede entweder eine Gefässschlinge oder ein Convolut eines strotzend erfüllten Gefässes einschliesst. Die Peripheire einer jeden Zotte ist von einer dichten Zellenlage gebildet, deren äusserste Schichte sich besonders an einigen Zotten, wie in *a*, ganz deutlich und entschieden der Cylinderform der Epithelialzellen assimilirt. — An vielen Zotten scheint das centrale Gefäss mitten in einem Zellencylinder eingebettet zu sein, indessen gelingt es, die Zellen nach längerer Präparation, wobei dieselben eine sehr innige Ver-

bindung unter einander beurkunden, so weit zu entfernen, dass ein zartstreifiger Papillarkörper *(b)* als Träger der Gefässschlinge zurückbleibt, dessen longitudinale Faserzüge an der Basis der Zotte in ihrem unmittelbaren Anschluss an das Grundgewebe verfolgt werden können. — Die Aehnlichkeit mit normalen Darmzotten — wenn man nämlich von der ungleichen Länge, Dicke und Vertheilung absieht — bezieht sich stellenweise sogar auf die Interstitien der Zotten wie in *c*, welche die Form von kurzen, gewöhnlich spitz endigenden Follikeln nachahmen und von einem Epithel ausgekleidet sind, dessen Cylinderform sich als Fortsetzung des Zottenepithels auch hier mit dem der Darmdrüsen übereinstimmend erweist. Einige dieser Zellen sind unter *C*, *d* bei 360mal. Vg. dargestellt; die Figur *e* stellt eine Partie des aus dem Grundgewebe gewonnenen Zerzupfungspräparates dar. Bei Anwendung von schwacher Essigsäure traten die Kerne überall markirt hervor; interessant war hiebei die wolkige Trübung des Präparates und die Erscheinung einer feinpunctirten, undeutlich streifigen Feuchtigkeit in der Umgebung und in den Zwischenzottenräumen, welches Phänomen bei einer ähnlichen Behandlung schleimhältiger Substanzen aufzutreten pflegt.

Entleerte Gewebsstücke. Die Untersuchung ungewöhnlicher, durch den Stuhlgang entleerter Gewebsfetzen ist in sofern belangreich, als durch den Nachweis eines physiologischen oder pathologischen Gewebes und der an demselben stattgefundenen Veränderungen als blossen Folgen der Einwirkung der Darmsäfte, vielleicht die *ursprüngliche Zugehörigkeit des Gewebes zum Darmtractus* erschlossen werden kann. Der Ausschluss von Ingestis, entschieden mit grossen Schwierigkeiten verbunden, wird nur unter Berücksichtigung aller Verhältnisse ausführbar sein, und das specielle Verhalten der Gewebsmasse mit Beachtung aller übrigen Symptome auf die Lostrennung einer ursprünglich im organischen Zusammenhange mit den Wandungen des Darmcanales gestandenen Textur hinweisen können. Schwieriger würde es sein, die Localität zu bestimmen, auf welche ein abgegangenes Gewebe zu beziehen. — Die ähnlichen Fälle, welche von Frerichs (s. *Verdauung* in Wagner's Hdwtb. d. Physiol.) und Virchow (l. c.) besprochen werden, beziehen sich auf unverdaute Speisereste, die in Divertikeln aufgehalten und macerirt wurden.

1. *Beobachtung. Durch den Stuhlgang entleertes polypenähnliches Gebilde* (am 23. April 1855) Fig. 9, *A*, *B*. Länge 5 Centim., Dicke 8—12 Mm. Form cylindrisch, ein Ende gerissen, das andere abgerundet, Oberfläche rauh, ungleichkörnig, von morschen, weisslichen, der Adicopire ähnlichen Bröckelchen bedeckt, Rinde zerreisslich, das Innere einen faserigen, vorwaltend in der Längenaxe streifigen, weisslichen Körper von beinahe sehniger Dichtigkeit darstellend, dessen gerissenes Ende schmutzig rothe Striemen zeigt. — Das Präparat wurde mir im Spiritus übermittelt und rührt von einer bejahrten Frau her, welche an chronischem Magen- und

Darmleiden behandelt wurde, die letzte Zeit hindurch wegen Appetitlosigkeit und häufigem Erbrechen, wobei zuweilen Spulwürmer abgingen, nur spärliche flüssige Nahrungsmittel zu sich nahm, und an hartnäckiger Stuhlverstopfung litt. Zuweilen sollen die Schmerzen derart gewesen sein, dass der Arzt an eine innere Incarceration des Darms gedacht und dieselbe auch als wirklich vorhanden angenommen hätte, wenn der Zustand sich nicht gebessert und die üblen Zufälle auf Aqua laurocerasi wenigstens für eine kurze Zeit nicht gewichen wären. Nach dem Abgang des in Frage stehenden Gebildes fühlte die Frau eine auffallende Erleichterung; doch scheint uns die Coincidenz des Excretbefundes mit der Besserung keinen sicheren Schluss zu gestatten.

Mikroskopische Untersuchung: Fig. 9, *C.* 1) Die oberflächliche, mürbe Masse enthält: Fettkörnchen, Oelfett in Tropfen, lose Epithelialzellen von Pflasterform. 2) Das gerissene Ende besteht aus starren Fasern, die sich an Zerzupfungspräparaten als *Bündel von spindelförmigen Elementen mit sehr schlanken*, durch Essigsäure nachweisbaren *Kernen* ergeben; zwischen den Bündeln verlaufen einzelne *elastische Fäserchen*, einige Bündel umschliessen dickwandige, von aufgelöstem Blut dunkelgebräunte *Gefässe*, deren Vorkommen sich auch in der Länge des cylindrischen Centralkörpers nachweisen lässt. Viele dieser Bündel verändern sich bei Zusatz von Essigsäure gar nicht; sie enthalten dicke plumpe Fasern, und quellen nach längerer Einwirkung des Reagens leicht auf, ohne Kerne erscheinen zu lassen. 3) Die centrale Hauptmasse besteht zum Theil aus *Faserzügen*, deren specielles Verhalten dasselbe ist wie an dem gerissenen Ende; in überwiegender Menge kommen jedoch *Fettzellen in einem fibrillären Bindegewebstroma (C)* zum Vorschein, welches dieselben zu grösseren und kleineren Areolis umspinnt und ungemein leicht zerreisslich erscheint. Hie und da lagern gelbe und braune hellglänzende *Pigmentkörner* zwischen den Balken des Gerüstes. Die Fettzellen haben überall eine bedeutende Grösse und runde pralle Form, ihre Fläche ist jedoch von den feinsten Fetttröpfchen und punktförmigen Tupfen verdunkelt, und aus dem Inneren derselben schimmert hie und da eine undeutliche krystallinische Substanz durch. 4) An der Grenze zwischen dem Faser- und Fettgewebe und der oberflächlichen mürben Masse lässt sich eine *gerunzelte Membran* darstellen, welche aus einer zarten homogenen Ausbreitung mit spärlichen Kernen besteht; ihre äussere Fläche zeigt ausser dem fettbreiigen Beschlag kein weiteres näher bestimmbares Detail.

Aus dieser Darstellung erweist sich das gerissene Ende als die Basis oder der Stiel, das andere als die Spitze eines Gebildes, welches vermöge seiner Textur als ein von einer Schleimhaut überzogenes *Lipom* zu bezeichnen wäre, wenn dieser Annahme ein häufiges Vorkommen des Lipoms im Darm zu Hilfe käme. Die Beschaffenheit des abgerissenen Stieles wiese auf eine stattgehabte Zerrung hin, wodurch die Gefässe wie durch Torsion obliterirt und das Ganze durch Auffaserung getrennt wurde. Die Veränderungen des Gewebes an der Oberfläche und im Inneren müssten als Folgen der Einwirkung der Darmcontenta betrachtet werden, wenn sie an Geweben, welche dem Kochen und der Verdauung ausgesetzt waren, den Zusammenhang nicht auch in dieser Weise intact liessen; dafür spräche auch der Umstand, dass die Patientin seit längerer Zeit nur spärlich Flüssigkeiten genossen und nach angehaltener Entlerung einen Stuhl gehabt hatte, mit welchem das Lipom zum Vorschein kam und die Beschwerden wichen. —

2. *Beobachtung. Abgang eines durch Strangulation nekrosirten Darmstückes.* Dieses Ereigniss kam im Verlaufe der Jahre 1850—1858 dreimal vor und ist kein so aussergewöhnlicher Gegenstand, um erst umständlich besprochen werden zu müssen. (Seltener ist jedenfalls die Entleerung eines nekrotischen Gewebsfetzens durch die Harnwege, wovon mir ein Fall von Prof. Güntner mitgetheilt wurde). Auch hiebei bemerkt man nach einer länger andauernder Störung der Darmfunction bald nach dem Abgange des Gewebes eine auffallende Erleichterung im Befinden, welches vordem durch die drohendsten Symptome einer inneren Incarceration gefährdet war. Die nekrotischen Darmstücke haben gemeinhin ein morsches, schmutzig graubraunes Aussehen; nach Abtrocknung der Häute, die von trüber Flüssigkeit durchtränkt sind, lässt sich bald eine membranähnliche, glatte Ausbreitung, bald die faserige Spaltbarkeit nachweisen; die Elementar-Untersuchung constatirt höchstens Binde- und Fettgewebe des Mesenteriums, äusserst schwierig die Musculatur, nie Elemente der Schleimhaut. Die Präparate sind in der Regel von Tripelphosphaten und erdigen Sedimenten stark durchsetzt und verdunkelt. — Die Fälle von Abgang solcher Darmpartien sind in der Chirurgie längst bekannt und oft beschrieben; sie lassen oft eine ungünstige Prognose stellen, indem Darmhypertrophie, Adhäsionen, Stricturen und ähnliche Hindernisse der Stuhlentleerung bleibend sind. (Vgl. Alexander Monro: Remarks on intussusceptio, Phys. und Lit. Etsay T. II. — William Domgall: History of a case of ileus in which a considerable portion of the intestine was voided by stool; in Medical Commentaries, T. 9.)

3. *Beobachtung.* Ein vielgereister ansehnlicher Herr, der wegen seiner eigenthümlichen Unterleibskrankheit alle möglichen ärztlichen Notabilitäten von Europa consultirt hatte, kam im Sommer 1857 nach Prag und präsentirte dem k. k. Ober-Stabsarzt, Herrn Dr. Russheim, ein durch den Stuhlgang entleertes Gewebe, welches in ziemlich morschem Zustande, an einem Rissende von coagulirtem Blute durchsetzt und in Alkohol conservirt war. Die Vermuthungen der früheren Aerzte scheinen sich zumeist auf Hydatiden oder Echinococcus-Säcke oder ein Pseudoplasma bezogen zu haben; das grösste der abgegangenen Gewebsrudimente von 1 Zoll Länge, 5—8 Lin. Breite liess an mikroskopischen Schnittchen unzweideutig die Elemente eines Bindegewebe- und eines doppelten Muskelstratums sehen, deren Anordnung mit den Darmschichten übereinstimmte, und es wäre nach diesem Befunde mit grosser Wahrscheinlichkeit eine stattgehabte Invagination des Darms anzunehmen, die sich nun hoffentlich zum Wohl des Patienten auf diese Art in Lösung befand.

4. *Beobachtung.* Bemerkenswerth in negativer Beziehung ist der folgende Fall, der mir in der jüngsten Zeit durch die Gefälligkeit des Collegen H. Dr. Franke mitgetheilt wurde. Die historische Notiz lautet:

„J. B., Lohgerbergeselle aus Deutschprausnitz, Bezirk Trautenau gebürtig, 29 Jahre alt, von grosser Statur, starkem Knochenbau, entwickelter aber schlaffer Musculatur, ziemlich blühendem Aussehen, früher stets gesund, zog sich im Monate December 1855 durch Verkühlung und Diätfehler einen acuten Magenkatarrh zu, wurde deshalb in Wien, wo er damals in Arbeit stand, anfänglich zu Hause, später in einer öffentlichen Krankenanstalt behandelt, und nach 14 Tagen gebessert entlassen. Da derselbe jedoch bald wieder in Arbeit trat, und sich in Folge dessen nicht

genügend schonen konnte, so brach die Krankheit wieder aus, nahm jedoch seiner Erzählung nach einen mehr chronischen Verlauf wieder an. Von da ab gebrauchte er theils Hausmittel, theils abführende Pillen, worunter auch die Morison'schen — verschlimmerte sich jedoch dadurch sein Uebel derart, dass er zeitweilig die Arbeit aufgeben musste. Namentlich waren es kolikartige Schmerzen im Leibe, und eine hartnäckige Stuhlverstopfung, die ihn von Zeit zu Zeit ans Krankenbett fesselten. Dabei nahmen seine Kräfte allmählig ab, so dass er nur die leichteren Arbeiten seiner Profession zu verrichten im Stande war. — Die hartnäckige Stuhlverstopfung bewog ihn zu *drastischen* Abführmitteln Zuflucht zu nehmen, und er behauptet, sich durch dieselben jederzeit Linderung verschafft zu haben. Eine entsprechende Diät wurde während dieser Krankheit *nicht* beobachtet. — Das andauernde Unwohlsein zwang den J. B. zu Weihnachten 1857 in seine Heimat zurückzukehren; er gebrauchte da abermals verschiedene Volksmittel, worunter die meisten, der Wirkung nach zu urtheilen, höchst wahrscheinlich drastische Abführmittel waren. Erst im Februar 1858 kam J. B. ambulatorisch zu mir, und erzählte mir das Obengesagte. Ich schrieb ihm eine geeignete Diät vor, und leitete ein theils gelind solvirendes, theils tonisches Heilverfahren ein, und trug ihm auf, seine Stühle zu beobachten, was er bisher nie gethan hatte. Acht Tage später brachte er mir abgegangene Gewebsstücke, welche vorliegen; ob dergleichen schon früher im Stuhle vorhanden waren, wusste er nicht anzugeben. Anfangs April l. J. kam J. B. abermals zu mir, und bemerkte, dass er sich jetzt viel wohler fühle, namentlich von den heftigen Kolikschmerzen und der Stuhlverstopfung befreit sei, einen besseren Appetit habe und sich kräftiger fühle, weshalb er nach Wien zurückzukehren beschlossen habe. Auch erwähnte er, dass nunmehr jetzt viel seltener, und nur dünne „weissliche Häute" im Stuhle seien. Seit der Zeit sah ich ihn nicht wieder."

Mikroskopischer Befund: 1. Ein 6 Zoll langes, 1—1½ Zoll breites Stück, längsstreifig, fest zusammenhängend, jedoch in der Längenaxe leicht spaltbar, besteht durchaus nur aus *Sehnengewebe*, welches keine andere Veränderung, sondern blos eine Lockerung in Folge seines längeren Aufenthaltes in warmen Flüssigkeiteu erfahren hatte. — 2. Eine zarthäutige cylindrische Röhre von 14 Lin. Länge mit einem Lumen von 5 Lin. Durchmesser in blättrige Hüllen spaltbar, die sich in Ringform und in der Längendimension abziehen lassen; das Detail zeigt gefensterte Membranen und elastisches Gewebe von einer dickwandigen *Arterie.* — Ein zweites Stück von ¼ Zoll Länge verhält sich ebenso. — 3. Zahlreiche lose Gewebsfetzen von unregelmässigen Formen und von ungleicher Grösse und Dicke erweisen sich als Rudimente von dichtem *fascienartigem Gewebe* mit geringen Spuren von Fettgewebe. — Aus dem Befunde geht hervor, dass vorliegende Gewebe nur auf unverdauliche Ingesta (Sehnen, Arterien, Fascien) zu beziehen seien, welche aber in keinem nothwendigen und irgend wie nachweisbaren Zusammenhange mit dem Leiden stehen, es wäre denn, dass man sich bei einem zu Diätfehlern disponirten Individuum der Vermuthung überliesse, dasselbe sei bei seiner animalischen Kost selbst während der Krankheit nicht sehr wählerisch gewesen, durch Retention einzelner Speisereste sei die Kolik u. s. w. unterhalten worden, und Patient habe die durch Molimina entleerten unverdauten Häute zu seiner persönlichen Beruhigung für die Materia peccans gehalten.

5. *Reihe.* Endlich gehört hierher die mikroskopische Untersuchung der einer unmittelbaren Inspection mehr zugänglichen Vorkommnisse im Endtheile des Rectum und namentlich am Anus (Hämorrhoiden, Polypen, Hauthypertrophien, Krebs). Welche erspriesslichen Dienste das Mikroskop für die Diagnostik in solchen Fällen bietet, hoffe ich bei einer anderen Gelegenheit ausführlich zu erörtern, und zugleich den Werth der optischen Kraft für die klinische Untersuchung des Uro- Genital-Systems als Pendant zu der von mir zuerst geführten Diagnose des Harnblasenkrebses (Viertelj. Bd. 49, 1856) zu erproben.

Parasiten. Am häufigsten kommen bei Untersuchungen der Darmexcrete die *Eier* der bei uns gewöhnlichen *Enthelminthen* (Fig. 11) zur Beobachtung und zwar besonders in flüssigen Stühlen, ohne hiebei an eine bestimmte Krankheit der Darmschleimhaut gebunden zu sein. Der Nachweis von diesen Eiern ist nur mittelst des Mikroskopes zu führen und sowie der Abgang von den erwachsenen Thieren, welche übrigens vom ungeübten Auge leicht übersehen wird, der einzig sichere Beleg für die Diagnose der Wurmkrankheit. Ich habe unter Fig. 11 die gemeinsten Formen von Enthelminthen-Eiern verzeichnet, wie sie bei einer etwa 280mal. Vergrösserung aussehen; die absoluten Maasse, die ich nach mehrmals vorgenommenen Messungen als Mittelwerthe anführe, stimmen mit denen der älteren Autoren, wie sie auch Küchenmeister anführt, ziemlich überein, und geringe Schwankungen kommen ohnedies auf Rechnung der individuellen Grösse. Es wäre gewiss nicht überflüssig, naturgetreue Abbildungen der Enthelminthen selbst zu liefern, da alle bisher gelieferten noch vieles zu wünschen übrig lassen; indessen gehört dies mehr in einen completen Atlas der Darmpathologie als zu der vorliegenden Aufgabe.

Ascaris lumbricoides (Spulwurm). Runde Eier messen durchschnittlich 0.087 Mm. im Diameter, bei den oblongen (*A*) finden wir die Länge = 0.0825 Mm., die Breite = 0.0452 Mm. Es sind die grössten von allen; im frischen Zustande zeigen sie eine helle, zackig-lappige Hülle einer eiweissartigen Substanz, welche eine Mächtigkeit von 0.0075—0.0090 Mm. besitzt und sehr vergänglich ist. Bei den grössten oblongen Eiern bemerkt man während des Rollens in der Profilansicht die Bohnenform (*B*) mit einem seitlichen hilusartigen Eindruck. Der doppelte scharfmarkirte Contour der Eisschale, der den Dotter in der Durchschnittsansicht wie ein Ring umgibt, ist bei diesen Eiern viel dicker als bei den folgenden.

Trichocephalus dispar (Peitschenwurm). Die Eier (*C*) sind

oval, an beiden Polen abgestutzt, die Eischale doppelt und kräftig contourirt, und so wie der Dotter bräunlich gefärbt. Länge = 0.054 bis 0.058 Mm., Breite (in der Mitte) = 0.025 Mm., an den Polen = 0.010 Mm.

Oxyuris vermicularis (Pfriemenwurm) (*D*). Die Eier sind meistens hell, die Eischale doppelt, jedoch zuerst contourirt, Form oval, häufig asymmetrisch, so dass die eine Krümmung (wie bei der Krystalllinse) mehr convex ist als die andere; zuweilen findet man an der stärker convexen Seite nächst des einen Poles eine seichte Depression, die als schwache Verdunkelung oder als zarter Streifen sichtbar wird. Länge = 0,0516—0.0560 Mm., Breite = 0.0225—0.0258 Mm.

Taenia Solium (Bandwurm). Die Eier sind kugelrund (*E*), die Schale von 3—4 scharfmarkirten kräftigen Contouren gezeichnet, blassbräunlich oder dunkelbraun (bis ins vollkommen Opake) tingirt und von allen Eiern der Enthelminthen die stärkste. Ihre Grösse variirt zwischen 0.040—0.45 Mm. im Durchmesser und darüber. An den reifen Eiern der Taenia gewahrt man im normalen Zustande innerhalb der Hülle (Chorion, *a*) den Zellenkern (Nucleus mit Nucleolus, *b*), das Keimbläschen (die Embryonalkugel, c) und einen Fettkörnchenhaufen (die Dotterkugel, d). — Die Eileiter, welche bei diesen Thieren die Eier enthalten, werden im getrennten Zustande, wenn Bandwurmglieder abgehen, einigermassen durchsichtig und lassen sich wahrscheinlich in Folge der Maceration in den Darmcontentis sehr leicht entfernen; ein blasser Saum, der zuweilen an isolirten Taenia-Eiern als äusserste Hülle wahrgenommen wird und von Fettkörnchen und Oeltröpfchen durchsetzt erscheint, rührt wahrscheinlich noch vom Eileiter her und löst sich ebenso bald auf wie die zarte hyaline Umhüllungsmasse an den Ascariden-Eiern.

Nebst diesen thierischen Objecten können die Haken von *Echinococcus* (und selbst von *Cysticercus*) bei Durchbruch ihrer Lagerstätte und Entleerung in den Darm zur Beobachtung kommen, und der mikroskopische Nachweis derselben in den Darmcontentis ist der allein gültige Beleg für die genaue Diagnose ihres Vorhandenseins in irgend einer Cyste. Die beigegebenen Umrisse der zerstreut vorkommenden Haken (Fig 11, *F. G.*) versinnlichen die relative Grösse dieser ungemein resistenten Horngebilde bei den Blasenwürmern, welche bekanntlich verirrte und incystirte Scolices von Bandwurmspecies sind; die Grösse der Echinococcus-Haken (*F*) = 0.0258

Mm. verhält sich zu der von Cysticercus-Haken (*G*) = 0.1290 und 0.1720 Mm. wie etwa = 3 : 15 : 20. *)

Ueber einen *Echinorrhynchus*, den ich im Dünndarm eines in Prof. Löschner's Kinderspital an Leukämie verstorbenen, 9 Jahre alten Knaben (Kořina Adolph, am 9. Aug. 1857) gefunden habe, theile ich die beifolgende Abbildung Fig. 12, *A*, *B*, *C*, *D*, und die dem frischen Objecte entnommenen Maasse mit.

Körperlänge 5.60 Mm. — grösste Breite 0.60 Mm. — Länge des Kopfes (resp. Rüssels) 0.36 Mm. — Breite desselben am vorderen Ende 0.34 Mm. — an der halsförmigen Einschnürung 0.24 Mm. — Breite am Hinterleib 0.40 Mm. — Länge der Rüsselscheide (vom vorderen Kopfende bis zum Grunde der blindsackähnlichen Begränzung der Scheide) 0.64 Mm. — Durchmesser der Eier (oder vielmehr placentaartigen Zellengebilde) schwankend zwischen 0.026—0.040 Mm. und zwischen 0.0043—0.0086 Mm. — Länge der gekrümmten Haken: an der kleinen Curvatur: 0.0774 Mm.—an der grossen Curvatur 0.1032 Mm. — Ihre Dicke an der Basis: 0.0258 Mm. — in der Mitte 0.0129 Mm. — Die Haken stehen alternirend in 8 Reihen (B, auf der einen Fläche gezählt, obwohl wegen der zahlreichen Kreuzungen schwierig erkenntlich), die der Längenrichtung des Rüssels entsprechen, in jeder Reihe zählt man fünf Haken deutlich, der sechste (unterste) Haken ist undeutlich ausgeprägt und von den oberen, die nach einwärts gekehrt liegen, überdeckt und gekreuzt. Zur Seite des Grundes der Rüsselscheide liegen zwei dunkel granulirte Körper (Lemnisci), mittelst bandartiger Streifen an die Aussen- und Unterfläche der Scheide befestigt; das Ligam. suspensorium, die „Glocke" und der Fruchtleiter sind scharf begränzt und dunkler contourirt als die Umgebung derselben, nämlich das Parenchym, worin die Placenten innerhalb des Muskelschlauches, eine Stunde lang in kreisender Bewegung beobachtet wurde. — Nach einer mündlichen Rücksprache mit dem H. Prof. Stein, der ein specieller Forscher der niederen Thierclassen und eine verlässliche Autorität über diese Eingeweidewürmer ist, die derselbe zum besonderen Gegenstande seiner gründlichen Untersuchungen gewählt hatte, konnte das Thier allem Anscheine nach, wofür auch das lebensfrische Aussehen desselben sprach, längere Zeit im menschlichen Darm lebend zugebracht haben, und den Unterleib des Menschen sehr wohl zu seinem Wohnorte wählen, da es erwiesen ist, dass von den Enthelminthen, die man bei den Haussäugethieren und beim Affen gefunden hat, nach und nach auch beim Menschen Repräsentanten entdeckt wurden. Der *Echinorrchynchus* kommt beim Schweine vor und zwar der *E. gigas*, mit welchem unser Fund die meiste Aehnlichkeit hätte, obwohl derselbe ein sehr junges, mithin unentwickeltes Individuum ist, dessen Bestimmung daher nicht mit gehöriger Schärfe ausführbar ist. Die Annahme einer Verirrung des Parasiten aus irgend einem anderen Wohnthiere in den Darmcanal des Knaben wäre ebenso unerwiesen als unerklärlich, da die nächsten Vermuthungen und Bedingungen einer solchen Wanderung, wie

*) *Acarus dysenteriae*, Linn. (Höfle l. c. S. 43 der Anmerkungen) wurde im Monate August 1858 im Kinderspital beinahe in jedem Tropfen einer dysenterischen Entleerung gefunden und gesammelt, jedoch als ein inconstantes Epiphaenomen ermittelt.

man sie auch denken wollte, im Kinderspital entfallen; indessen wäre selbst für diesen Fall die Thatsache nicht ohne Interesse, dass das Thier lebend der Leiche entnommen und eine Stunde lang unter dem Mikroskope betrachtet, die Kreisbewegung der zelligen Körper innerhalb des Muskelschlauches wahrnehmen liess, welche eine, wenn auch posthume, Contraction der musculösen Elemente voraussetzen lässt.

Das Thier gehört in die Classe der Würmer und ist ein Repräsentant der Familie *Acanthocephali* (Vgl. Victor Carus, Icones zootomicae Taf. VII. Fig. 2—10 nach Prof. Stein). Der spindelförmige Körper *(A)* hat ein Kopfende *(a)* mit dem charakteristischen Rüssel, und ein Genitalende *(b)* mit der Mündung des Geschlechtsapparates. Ein Darmschlauch fehlt gänzlich, und die Aufnahme der Ernährungsflüssigkeit wird somit, wie bei den Cestoden und Nematoden, durch Endosmose der allgemeinen Decke vermittelt, welche als *Cutis* die äusserste, zart und scharf contourirte Begränzung des Thieres bildet. Auf diese folgt nach innen zu ein dunkler Saum, der die Cutis von dem eigentlichen Muskelschlauche des Thieres trennt und als Hohlraum eine opake *Körnermasse* enthält, in der sich eine Bewegung der Flüssigkeit nach oben gegen das Kopfende wahrnehmen lässt. Die Begränzung dieses Hohlraumes fand ich durch regelmässige Querstreifen in gleich grosse viereckige Feldchen, nach Art eines Epithels abgetheilt; die Mitte eines jeden Feldchens nahm ein kernartiges Körperchen ein *(c)*. — Der *Muskelschlauch (d)*, der die Eingeweide unmittelbar einschliesst, besteht aus glatten Muskelfasern, die in parallelen Zügen die Querrichtung der Leibesaxe befolgen und somit in ihrer Gesammtheit einen ringförmigen Muskelkörper darstellen. Der Muskelschlauch hat nur an seinem unteren Ende eine Oeffnung, nämlich die Genitalmündung; nach oben hin schmiegt sich derselbe unter allmäliger Verdünnung seiner Wandung an die *Rüsselscheide* an, deren unteres Ende von demselben seitlich umhüllt wird, *(B, e)*. Die *Rüsselscheide* selbst ist ein Blindsack-ähnlicher Körper, der ungefähr den sechsten Theil der gesammten Körperlänge einnimmt *(f)*; an seinem vorderen Ende trägt derselbe den igel-stacheligen *Rüssel*, im Inneren bemerkt man neben einer zufälligen Faltung der hautartigen Scheidenwand nichts anderes als eine Gruppe zelliger, zart contourirter Gebilde, welche im Grunde der Rüsselscheide *(B, g)* zu einem länglich runden Haufen zusammengedrängt, diese Partie einigermassen verdunkeln. Man erklärt diesen Zellenhaufen für ein *Gehirnganglion* und für die einzige Spur von Nervengewebe bei diesen Thieren. — Der vorgestreckte *Rüssel* erweist sich als ein dicker compacter Körper von stumpfconischer, mit der Basis nach oben gekehrter Form. Er ist ein blosser Haftapparat, ohne Saugmündungen und ohne Höhle. Seine Oberfläche ist uneben rauh, sowohl im Sinne der Hakenreihen gefurcht und gerifft, als auch entsprechend dem Ansatz der einzelnen Haken, mit warzigen Erhabenheiten versehen, die sowohl einzeln als auch zusammen wahrscheinlich einen compacten musculösen Körper darstellen. Wir wollten das Unicum nicht opfern, um durch Zerstörung desselben zu einiger Einsicht über das Wesen dieses kräftigen Körpertheiles zu gelangen. — An der unteren, der Leibeshöhle zugewandten Fläche der Rüsselscheide befestigen sich drei bandartige, zart contourirte, längsgestreifte Gebilde, wovon der mittlere als *Ligamentum suspensorium (A, h)*, die beiden seitlichen als *Musculi retractores* (*i, i*, nach oben und zur Seite geschoben) bezeichnet werden; alle drei ziehen gegen

das hintere Leibesende innerhalb des Muskelschlauches hin; die Retractoren inseriren sich, ohne eine bestimmte Demarcation ihres hinteren Endes, allmälig verschwindend an der Innenfläche des Muskelschlauches. Das Suspensorium lässt sich bis in den Genitalschlauch verfolgen und hat den Zweck, die durch Contraction des Muskelschlauches hervorgestülpte Rüsselscheide zurückzuhalten und den Haftapparat, der die ganze Körperlast zu tragen hat, von innen und unten zu unterstützen; die Retractoren dagegen ziehen den Grund der Rüsselscheide zurück und stülpen somit den Rüssel mittelbar in die Leibeshöhle ein, so dass derselbe im retrahirten Zustande, wie beim Echinococcus und Cysticercus, mitten im Körper zu liegen kommt, wobei dann die Rüsselhaken in umgekehrter Richtung, nämlich mit den Spitzen nach oben, zu sehen sind. — Nebst dem Lig. suspensorium und den beiden Mm. retractores nehmen noch zwei eigenthümliche Körper, die sogenannten *Lemnisci (B, l, l)* ihren Verlauf um die Rüsselscheide, an deren oberem Ende sie unmittelbar unter dem Rüsselkörper ihren Ansatz finden. Man ist über die Natur und Bedeutung dieser walzenförmigen, nach oben zu dünn auslaufenden Organe noch nicht ins Klare gekommen und betrachtet sie muthmasslich als Hohlcylinder oder schlauchartige Parenchymtheile, welche die Communication zwischen dem extramusculären (subcutanen) Hohlraume und zwischen der eigentlichen Leibeshöhle vermitteln. Das Gewebe derselben erscheint in dem dünneren Theile deutlich gestreift, in dem hinteren dickeren Stücke dunkel und feinkörnig; in unserem Exemplare sind beide Lemnisci zusammen geschoben und nach oben an die Rüsselscheide gerückt verzeichnet, wie sie an dem eingelegten und befriedigend conservirten Präparate noch immer wahrgenommen werden. — Sowohl die Lemnisci als auch die extramusculären Räume enthalten bei vollkommen entwickelten Thieren zellenartige Hohlgebilde von länglich runder Form; der äussere Umriss dieser Höhlen ist scharf begränzt, das Innere enthält einen gleichfalls länglich runden feingranulären Kern, der von dem Zellenumriss durch einen gleichmässig breiten hellen Saum getrennt ist. Die Uebereinstimmung dieser Höhlen in den Lemnisci und in dem äusseren Raume, welcher mit der schwimmenden Körnermasse erfüllt ist, lässt in diesen Organen eben verwandte, der Saftströmung dienliche Gebilde vermuthen.

Höchst merkwürdig ist die Einrichtung des *Genitalapparates* bei diesen Thieren. An unserem Exemplare, welches ein Weibchen ist, bemerkt man am hinteren Leibesende eine, durch eine seichte Einkerbung markirte Oeffnung *(b)* der Cutis und des Muskelschlauches, von welcher aus in der Richtung nach oben gegen das Kopfende hin zwei cylindrische Schläuche mitten durch die Leibeshöhle verfolgt werden; der *untere Schlauch* stellt den *Uterus (u)* und die *Vagina (v)* dar, der *obere (x)* hat den Namen *Glocke* (von Siebold) erhalten und ist ein eigenthümlicher Oviduct, der die Eier auf eine ganz merkwürdige Weise befördert, um sie dem Fruchtsack oder Uterus zu übermitteln. Man findet nämlich an der Glocke, die in der Leibeshöhle gleichsam frei herabhängt und an dem Lig. suspensorium, welches mitten durch dieselbe zieht, befestigt ist, zwei Mündungen, eine obere, oder den nach der Leibeshöhle hinsehenden Eingang, und einen unteren Ausgang, der sich im entwickelten Zustande des Thieres trichterförmig zuspitzt und mit einer engen Oeffnung in den Fruchtschlauch (Uterus) eindringt. Die Bewegungen der Glocke dienen zur Beförderung

der Eier in der Weise, dass dieselben von dem sich erweiternden Eingang aufgenommen, gleichsam verschlungen werden und, wenn sie ihre völlige Reife erlangt haben, durch den Ausgang in den Uterus getrieben, wenn sie aber noch unreif sind, durch einen eigenen Seitenausgang ausgestossen und neuerdings in die Leibeshöhle geschafft werden, um bis zur völligen Reife herumzukreisen. Die Richtung der Pfeile versinnlicht in *C* diese Bewegungen.

Ein entwickeltes *Ei* (*D*, *a* nach V. Carus) hat eine oblonge weberschiffchen-ähnliche Form, deren grösster Querdurchmesser etwa den sechsten bis achten Theil der Eilänge beträgt. Nebst den drei hellen Eischalen bemerkt man im Inneren des scharfcontourirten Eies den feinkörnigen Dotter und einen zellenartigen Körper, wahrscheinlich das Keimbläschen. Die Anlagen zu Eiern sind oblonge, leicht gekrümmte Körper mit Kern und Kernkörperchen *(b, c)*; sie kommen in der Leibeshöhle *in Zellenhaufen* eingebettet vor, welche man für *Ovarien* oder *Placenta-ähnliche Gebilde* erklärt; darnach wären in jungen Thieren, wie in dem vorgefundenen Exemplare, die in dem Muskelschlauche frei herumschwimmenden Zellenelemente (*C*, zu beiden Seiten des Genitalschlauches) nicht für Eier selbst, sondern für die Anlagen der künftigen Ovarien oder Placentae zu halten. Einige darunter zeichneten sich durch ihre Grösse und länglich-runde Form, sowie durch einen deutlichen Kern aus, und diese möchten den unreifen Eiern mit einer Eischale oder ohne eine solche am ähnlichsten sein. *)

---

*) Diesen merkwürdigen — beim Menschen meines Wissens zum erstenmale vorgekommenen — Fund verdanke ich der vorsichtigen Eröffnung des Darms bei Leichenöffnungen, wie ich dieselbe zu üben gewohnt bin. Der Darm wird von der Cöcalklappe an unter *horizontaler Haltung beider Hände* (die Handteller nach oben gerichtet) in der Art eröffnet, dass die geknöpfte Branche der Scheere an dem nach abwärts gerichteten Ansatzrande des Darmrohres eingeführt wird, während die linke Hand den Darm in die spitzgeöffneten Winkel der Scheere hineinzieht. Man gewinnt dabei die Uebersicht der ganzen Schleimhaut in der Circumferenz des Rohres, ohne dass die in der Regel vis à vis dem Mesenterialansatz gelagerten Geschwüre und dergleichen Befunde zerschnitten würden. Der Daumen der linken Hand bewegt sich allein an der Schleimhaut des aufgeschnittenen Darmstückes, die Finger dagegen heben den in die Fläche ausgebreiteten Darm von unten und halten denselben von dem Augenblicke der Eröffnung an so lange dem beobachtenden Auge entgegen, als man den Inhalt des Darmrohres, ohne denselben abgespült zu haben, durchzusehen für nothwendig erachtet. — Es ist bekannt, wie leicht die viel grösseren Enthelminthen, Oxyuris und Trichocephalus, übersehen werden, und selbstverständlich kann der Umstand, dass jemand etwas nicht gefunden habe, unmöglich als vollgiltiger Beweis gelten, dass es nicht dagewesen sei. Wer nicht suchen kann, findet die kleinen Enthelminthen entweder gar nicht oder, wie es heisst, sehr selten. Prof. Zenker hat gezeigt, wie man die kleinen Männchen von Oxyuris vermicularis in grosser Anzahl gewinnen könne (Küchenmeister's Parasiten). — Seitdem gelang es mir nicht wieder, einen Echinorrhynchus zu finden und es wird der ferneren Aufmerksamkeit vorbehalten bleiben, das Thier ent-

Ueber das *Männchen*, welches ich für diese Species nicht kenne, führe ich nur nach dem Gattungscharakter und den mir zugänglich gewordenen Abbildungen von E. anthuris Duj. (vom Triton) und von E. angustatus Rud. so viel an, dass bei demselben ein am Suspensorium hängender erster und an diesem befestigt ein zweiter Hoden befindlich ist; aus jedem dieser Drüsenkörper tritt ein *Vas deferens* hervor, welches sich nach unten blasenartig erweitert und durch einen *Ductus ejaculatorius* an dem unteren Leibesende mündet. Hier befindet sich ein napfförmig aufgeblasener *Penis*, der als Begattungsorgan nicht in die Vagina zu dringen, sondern die Vulva aufzunehmen und umzuhüllen bestimmt ist. (Zu vergleichen mit der Penisscheide des Trichocephalus).

Distoma hepaticum. (Taf. III. Fig. 11, H.) Diesen seltenen, von Wenigen gesehenen Gast in der menschlichen Leber, fand ich bei einem 21 J. alten Italiener (Giovanni Battista Gerotto di Grisolera, Sect. Prot. N. 236, am 5. Juli 1858), der nach einer 2monatl. Krankheit an eitrigem Erguss im rechten Pleurasacke mit consecutiver Compression und Atrophie der Lunge im k. k. Garnisonsspital zu Prag gestorben ist. Ueber den Parasiten selbst, der sich nach einer genauen Untersuchung sämmtlicher Gallenwege nur in der Einzahl und zwar in einem Gallengange des grossen Leberlappens frei nachweisen liess, kann ich keine neuen Bemerkungen beibringen, und verweise auf die bekannten Beschreibungen desselben in den Lehrbüchern. — Von mehr Interesse ist es hier, auf einen Zustand der Leber hinzuweisen, der freilich ganz unabhängig von dem Leberegel bestand, nämlich eine ausgezeichnete *Hypertrophie der Leber und sämmtlicher Drüsenorgane* des Unterleibes, welche bei der mikroskopischen Untersuchung und Vergleichung mit normalen und atrophischen Texturen instructive Anhaltspunkte bot. Die Leberacini maassen an den durch den Pfortaderblutgehalt scharf markirten Inselchen der Durchschnittsfläche 1,5—1,8 Mm. im Diameter, während die einer normalen Laber höchstens auf 1,2 Mm. gelangen, die einer atrophischen Leber aber auf 0,8—0,5—0,3 Mm. sinken. Die *Hypertrophie* der Drüsensubstanz war sowohl eine *numerische, als auch eine elementare* (oder enchymatöse), das heisst, die Anzahl der Drüsenzellen war in Zu-

---

weder als einen seltenen Parasiten des Menschen zu adoptiren oder als einen verirrten Wanderer dahinzustellen. — Von seltenen Parasiten besitze ich ein von Pacini in Florenz geschenktes Präparat von *Oestrus hominis*. — Ueber sogen. Pseudoparasiten, namentlich Insecten im menschlichen Dauungscanal, habe ich einige merkwürdige Beobachtungen verzeichnet und (in der Plenar-Vers. d. Prager Doct.-Colleg. am 31. Oct. 1854) mitgetheilt; die Diagnose beruht hier auf dem Studium der comparativen Anatomie und Histologie.

nahme begriffen und es fanden sich zahlreiche, zwei- und mehrkernige Zellen vor, die sich durch Bildung von Querscheidewänden zu neuen selbstständigen Zellen abtheilten, und die vorfindlichen, völlig ausgebildeten einkernigen Leberzellen erreichten das ungewöhnliche Maass von 0,0258 Mm. und darüber, während die normalen 0,016 messen, die atrophischen dagegen auf 0.0086 und darunter sinken, indem weiterhin nur undeutliche Zellenreste in der Fettkörnchen- und Oeltropfen-Masse zu finden sind. — Gleichzeitig war nebst der Drüsenhyperterophie eine Hypertrophie des serösen Ueberzuges mit dem befriedigenden Nachweis des Zellenwachsthums in der subepithelialen Schichte und der Bindegewebsumwandlung bis zu beträchtlicher Schwartenbildung zu finden. In wiefern dieser Befund auf einen stattgefundenen (protrahirten) entzündlichen Vorgang zu beziehen wäre, möchte ich deshalb nur mit Rückhalt erledigen, weil jeder freie oder interstitielle Erguss vermisst wurde und der stärkere Blutgehalt des Leberparenchyms an den schwartig verdickten Kapselstellen einer Gefässentwickelung im Bereiche der Hypertrophie entsprach, worin die Gefässlumina gleichfalls gross und weit, somit auch ihr Inhalt reichlich sein musste. Die Textur zeigte nirgends die geringste Spur einer Degeneration und jedes Drüsenelement musste seiner Physiognomie nach als vollkommen functionsfähig anerkannt werden; es scheint aber gar nicht gerechtfertigt werden zu können, ein entschieden grosses, mit gehöriger Lebensenergie fungirendes Organ des entzündlichen Charakters verdächtig zu machen.

Infusorien. Prof. Malmsten in Stockholm fand im J. 1856 neue Intestinalthiere beim Menschen (Virchow's Archiv Bd. 12, S. 302), und zwar zuerst bei einem 38 J. alten Bootsmann, der nach überstandener Cholera lange Zeit hindurch ein Gefühl von Leere und Saugen im Epigastrium, Kollern im Bauche hatte, und abwechselnd an Verstopfung und an Koliken und Diarrhöe litt, wobei dünne Excremente von gelbgrauer Farbe und wenig excrementiellem Geruch, mit unverdauten Speiseresten, jedoch ohne Blut, mehrmals des Tages abgingen. Nach Verheilung eines kleinen Geschwüres im Rectum, welches an der gegen die Prostata liegenden Wand gelegen war und einen dünnen, etwas blutig gemischten Eiter absonderte, befanden sich im Stuhle, welcher sogleich nach seinem Abgang mikroskopisch untersucht wurde, so wie auch in dem mittelst eines Löffels durch Einführung eines langen Speculums heraus ge

nommenen frischen Darmschleime dieselben Infusorien wie im Stuhle, wovon Prof. Lovén eine kurze Beschreibung und Abbildung liefert.

(*Paramaecium? coli.*) Länge des Thieres 0.1 Mm., Form oval, drehrund, vorn leicht zugespitzt. Aeussere Haut mit Cilien besetzt in schief laufenden Reihen ohne Gitterung. Der Mund mit längeren Wimpern versehen, Oesophagus weit nach innen reichend. Der After der Bauchseite genähert. In der Leibeshöhle ein Nucleus, schwach contourirt; — zwei contractile Bläschen, das grössere in der Nähe des Anus, das kleinere in der Mitte der Rückenseite, mit äusserst langsamen, jedoch nicht unbedeutenden Formveränderungen; — endlich verschluckte Nahrungsstoffe, meist Amylumzellen und Fetttropfen. In lauwarmer Temperatur halten sich die Thiere beinahe 24 Stunden, sonst vergehen sie in kurzer Zeit, Zusatz von Wasser ist denselben nicht zuträglich.

Malmsten hält es für denkbar, dass dieses Infusorium in grosser Menge vorhanden die sogenannte Lienterie bedingen könnte, deren muthmasslicher Zusammenhang mit Ulcerationen übrigens dahingestellt bleibt. Sie fehlten zwar im Dickdarm an brandigen Ulcerationen der solitären Follikel nicht, in bedeutender Menge jedoch fanden sie sich im Schleime der gesunden Mucosa des Coecum und des Processus vermiformis.

Die gewöhnlichsten Infusorien sind die sogenannten *Vibrionen* (T. II. F. 4, *d*), die sich wohl von ähnlichen, in faulenden Flüssigkeiten vorkommenden nicht wesentlich unterscheiden, bei Stuhlentleerungen jedoch nicht unberücksichtigt zu lassen sind, weil sie in enormer Menge und lebhafter Bewegung unzweifelhaft einen Heerd von Zersetzung und Stagnation anzeigen, dessen Inhalt bei mangelhafter oder gänzlich fehlender Elimination als deletärer Stoff die Gewebselemente angreift und zerstört (Leberabscess und Gangrän, sphacelöse Geschwüre, Diphtheritis, Follicularverschwärung und Fistelbildung u. dgl. m.)

Cercomonas intestinalis (mihi). Taf. I. Fig. 2, z. Vg. 640 Amici. Mit entschiedener Gewissheit kann ich das myriadenweise Vorkommen dieser Monade in gelée-artigem Schleimexcret bei Kindern verbürgen, eines einzelligen Infusoriums, dessen verwandte Formen Ehrenberg als *Bodonen* zusammenfasst, (Bodo = Grenzstein) und dieselben in die Milchstrasse der mikroskopischen Wahrnehmung versetzt.

Der Gattungsname *Cercomonas* kommt (sowie auch Donné's Trichomonas) bei Dujardin vor. Beide Thiere sind delicate Objecte, besonders Cercomonas erfordert eine starke optische Kraft und günstige Beleuchtung. — Grösse: 0.018—0.021 Mm. in der Gesammt-Länge, 0.0086—0.011 Mm. in der Breite. — Form: ähnlich einer Kaulquappe; an dem zugestutzten Ende mit einem rundlich-ovalen Saugnapf versehen, in dessen Tiefe zwei kernartige Gebilde durchschimmern. Der Schwanz — 0.003—0.004 Mm. lang, 0.0008—0.0016 Mm. dick. — Bewegungen:

Locomotion in Kreistouren gleich dem Schwalbenflug, — dann schwankend, wobei die Profilansichten wahrgenommen werden; nebstdem vibrirt bei ruhenden Thieren der Ruderschwanz wie bei Spermotozoiden und in agone öffnet und schliesst sich der Saum des Saugnapfs undulirend, was einer Athembewegung ähnlich sieht. Diese letztere wurde mehre Stunden lang in dem frischen Excrete beobachtet; einzelne Thiere waren noch am anderen Tage kenntlich. Die jüngsten Formen gleichen ovalen, gallertig aufgequollenen Schleimkörperchen.

Entophyten. In der Leiche eines alten, an Cholämie in Folge von Verstopfung des Ductus choledochus durch ein Medullar-Carcinom verstorbenen Mannes fand ich an dem Ostium duodenale, vom Darmlumen aus, auf der Wucherung des Pseudoplasmas einen pflanzlichen Parasiten, der vielleicht als ein seltenes Epiphänomen wieder zur Beobachtung kommen könnte, und dessen ich hier daher in Kurzem erwähne. Der Pilz sass auf einem opaken Klumpen einer gelb-durchfärbten Matrix und erhob sich, wie die beigegebene Figur 13 (bei 180mal. Vg.) zeigt, mittelst eines Stieles kolbenförmig über das Niveau der Umgebung. Die Farbe desselben war lebhaft rubinroth; je nach der Grösse des Kolbens befanden sich im Inneren 1—4 zellige Körper der Länge nach geordnet. — Ich bedauere unendlich, dass es mir damals im Drange der obligaten Geschäfte nicht möglich war, über diesen Befund eine genauere Untersuchung vorzunehmen und etwas mehr als die blosse Zeichnung zu fixiren. Der Physiognomie nach ähnelte das Gebilde am meisten der Puccinia favi (Ardsten in: Gazette des hôpitaux, Paris 1851 p. 477 und: Annales des maladies de la peau et de la syphilis. Paris 1851. Vol. III, p. 281. Küchenmeister: Die pflanzlichen Parasiten 1855. p. 127, Taf. V, Fig. 6).

Ueber sonstige pflanzliche Parasiten, welche bisher nicht blos im Magen, sondern auch im Darmcanal gefunden wurden, kann ich nur die Bestätigung beibringen, dass man ihnen in krankhaften Stuhlentleerungen nicht selten begegnet; namentlich wären nach meinen Notizen zu erwähnen: 1. zarte Thallusfädchen von höchstens 0,0002—0.0007 Mm. Dicke, die zumeist dem *Leptothrix buccalis* gleichen (zufällig und bei Katarrh); — 2. Breite Thallusfäden, wie die von *Oidium albicans*, gewöhnlich 0.002=0.004 Mm. dick, mit Sporen, die der Gährungsalge gleichen (bei croupöser Dysenterie); — 3. Die *Sarcina ventriculi*, in diarrhoischen und dysenterischen Stühlen (übrigens auch im Harn und bei Lungengangrän angetroffen); — 4. *Cryptococcus cerevisiae*, in reichlicher Menge bei Diabetes (S. meine Notiz hierüber in Virchow's Archiv Bd. 11, 1857. S. 185 und Dr.

Petters: Untersuchungen über die Honigharnruhr, Vierteljahrschrift Bd. 55. S. 81.)

Mit wenigen Worten mag noch einiger *abnormen Färbungen* Erwähnung geschehen, die im Vorhergehenden nicht in Betracht gezogen wurden.

Die über die Natur der *Kalomel-Stühle* erlangten Resultate, mit deren chemischer Untersuchung sich vor Jahren Hornemann, Merklein und Rampold beschäftigt haben, lassen sich in folgende Sätze zusammenfassen: 1. Die eigenthümliche hellgrüne, oder grün- und grau-schwarze Färbung der Kalomelstühle lässt sich aus der chemischen Veränderung allein erklären, welche das Kalomel im Darmcanal erleidet, und welche schon bei blossem Zusammenreiben von Kalomel mit gelben Darmcontentis entsteht. — 2. Es kann dabei ebensowohl eine Reduction zu regulinischem Quecksilber (Hornemann) als zu Schwefelquecksilber (Merklein), vielleicht auch zu Quecksilberoxydul (Rampold) stattfinden. — 3. Durch vorhandene Galle wird wahrscheinlich nur die hellgrüne Färbung der Fäces bewirkt (Hornemann, Rampold), sie ist aber zur Hervorbringung namentlich der dunkleren Nüancen nicht durchaus nöthig. – 4. Endlich kann zufällig vor oder nach dem Gebrauch des Kalomels eine Gallenentleerung durch den Stuhl stattfinden, wie dies auch ohne vorausgegangenen Kalomelgebrauch vorkommt. Höfle führt diese Untersuchungen ausführlicher an, und kommt (S. 97) bezüglich der therapeutischen Anwendung des Kalomels zu dem Schlusse: dass Scrupeldosen, wenn auch nicht schädlich, doch überflüssig sind, da jedenfalls ein Theil des Kalomels doch unverändert den Darmcanal passirt. In unseren ärztlichen Kreisen, wo die innerliche Anwendung mineralischer Medicamente immer mehr beschränkt wird, werden wir wenig Gelegenheit finden, dergleichen Experimente an Patienten vorzunehmen, deren Genesung auch ohne diese erzielt wird oder überhaupt unmöglich ist.

Sulfas cupri. Bei profuser Darmentleerung, wo dieses Mittel in Anwendung kam, fanden wir im Excrete Veränderungen, die auch durch die Behandlung einer ähnlichen reinen Excretmasse mit einer verdünnten Solution des Salzes hervorgebracht werden. Die Farbe wird dabei je nach dem Gallengehalt viel dunkler grün als man nach der geringen Quantität des Mittels vermuthen sollte; die Schleimflüssigkeit gerinnt zu einer flockigen und zähekrümmeligen Masse, die geformten Elemente, namentlich Eiterzellen, schrumpfen zu opaken, dunkel und rauh contourirten Körperchen

bis zum Unkenntlichwerden ihrer ursprünglichen Physiognomie. Bei reichlichem Wassergehalt und Beimengung fettiger Bestandtheile gewann die Flüssigkeit einmal das gewöhnliche Aussehen von Meconium, wovon es sich jedoch durch das Mikroskop hinreichend unterscheiden liess. Zu forensischen Zwecken ist die chemische Reaction in solchen Fällen durchaus nothwendig; in physiologischer Beziehung sind die chemischen Analysen der Exportstoffe dieses Weges noch lange nicht so weit gediehen wie die der Harnwege, und die quantitative Betimmung derselben wäre besonders für die Pathologie und Pharmakodynamik von höchstem Belang.

Meconium. In kleinen Portionen ist die Farbe und die sonstige Beschaffenheit desselben kaum als sicheres Merkmal anzusehen; die mikroskopische Diagnose ist allein beweiskräftig. Der Befund ist nicht der von Darmepithel und Schleim, wie man in den meisten Büchern zu lesen bekommt, sondern — wie Förster noch unlängst (Wiener Medic. Wochenschrift, August 1858) beschrieben hat — identisch mit dem des Vernix caseosus, indem ein Theil der Hautschmiere mit den Epidermisschuppen und Wollhaaren in der Amniosflüssigkeit suspendirt und durch den Mund in den Darmcanal gelangt, wo nur noch die Gallenbestandtheile hinzutreten, während das Wasser von der Darmschleimhaut aufgesaugt und wahrscheinlich durch die Harnwege wieder secernirt wird. — Das Vorkommen von Wollhaaren ist bei uns schon früher bekannt und forensisch gewürdigt worden; Maschka erwähnt derselben ausdrücklich bei Gelegenheit einer Besprechung (Vierteljahrschrift, Bd. 56. Lit. Anz. S. 14); beachtenswerth ist jedoch die Anregung Förster's, den mikroskopischen Befund des Meconium von dessen erstem Auftreten im Foetus bis zum Abschluss des intrauterinen Lebens zu verfolgen und den fötalen Stoffwechsel der Gewebe und Flüssigkeiten genauer zu ermitteln.

Melaena. Sie ist oft von Ingestis (z. B. beim Genusse schwarzer Beeren) und von medicamentösen Stoffen (z. B. von Syr. diacodii, Succus liquiritiae) nur durch das Mikroskop mit Gewissheit zu unterscheiden und lässt, sobald sie festgestellt ist, immer ein Blutextravasat voraussetzen, dessen Farbenveränderung und theerartige Beschaffenheit mit den räumlichen Verhältnissen, der Menge und der Dauer der Einwirkung des Magen- und des Darmsaftes in Proportion steht. Corrosionen, Geschwüre, Krebs, mechanische Verletzungen von Gefässen der Darmschleimhaut, bei Koprostase und Unwegsamkeit des Darm-

rohres, Texturerkrankungen und Perforationen der Gefässwandungen und dgl. liegen in solchen Fällen den sogenannten „Obstructionen“ zu Grunde.

Ueber *farblose Stuhlentleerungen* besitze ich mehrere Verzeichnungen, welche auf verschiedene Bedingungen (Gallensteine, Krebs, Hypertrophie u. dgl.) zurückzuführen waren. Nachstehender Fall hat mein Interesse lebhaft erregt, obwohl ich gestehen muss, dass mir die von dem Praktiker mitgetheilte Beobachtung, sammt dem mikroskopischen und dem chemischen Befunde die erwünschte klare Einsicht nicht geboten hat.

A. F., eine bejahrte Frau, wurde im Jahre 1855 längere Zeit hindurch an sogenannten Hämorrhoidalbeschwerden behandelt, bekanntlich ein Collectiv-Ausdruck für Leiden aller Art, bei denen mancher Praktiker keine bestimmtere Diagnose machen kann. Die hervorragendsten Symptome waren hartnäckige Stypsis, Unterleibschmerzen, Dyspepsie, Erbrechen der eingenommenen Flüssigkeiten und Medicamente (Abführmittel). Am 25. März wurde mir ein hühnereigrosser Knollen einer trockenen, wenig riechenden Faecalmasse zur Untersuchung übermittelt, welcher im frischen Zustande, so wie auch später, von graulichweisser Farbe, leicht bröcklich war und angeblich mit grosser schmerzhafter Anstrengung entleert wurde. Die Frau soll zu dieser Zeit ikterisch gewesen sein und seit vierzehn Tagen keinen Stuhl gehabt haben; nachher soll auf den Gebrauch von Ol. Ricin. Besserung des Zustandes eingetreten sein, ohne dass sich je die üblen Zufälle wiederholt hätten. — Die *mikroskopische Untersuchung* ergab eine grosse Menge von umfänglichen *Kugeln blassgelblicher Fettsalze*, die sich globulär desaggregirten und erwärmt in einen seifenartigen Brei zerflossen; zahlreiche *Fetttröpfchen* und *Fettinseln*, nebstdem zahlreiche stangenförmige und *nadelspitze Krystalle*, rhombische Prismen mit spitzauslaufenden Polen, von der Grösse 0.007—0.012 Mm., Drusen feiner nadelförmiger Krystalle, dann eine beträchtliche Quantität eines erdigen *Detritus*, der sich in Säuren nur zum Theil löste, spärliche Trümmer von pflasterförmigen Epithelzellen und deren Kerne, Fragmente von Pflanzenfasern, mit den vorigen Bestandtheilen zu einer schwer entwirrbaren Pasta zusammengeballt. — Auffallend war der *Mangel an Gallenpigment*, indem auch unter dem Mikroskope keine Spur der rothbraunen und braungelben Plaques und Fetttropfen-ähnlichen Figuren nachgewiessen werden konnte; Blut- oder Fibrin-Bestandtheile waren gewiss nicht vorhanden, Albumen konnte nur in geringer Menge zugegen sein.

Die *quantitative chemische Analyse*, welche H. Dr. Krell vorzunehmen die Gefälligkeit hatte, ergab folgendes Resultat: In 100 Theilen der Fäcalmasse: Wasser 32,900 pCt.; fettsaure Salze 62,842 pCt. (Margarin-Säure, etwas wenig Stearin- und Elain-Säure, grösstentheils mit Natron verseift, aber auch in geringer Menge als freies Fett vorhanden). — Im Wasser unlösliche Salze (phosphorsaurer Kalk und phoshorsaure Magnesia): 1,166 pCt. — Im Wasser lösliche Salze (Chlornatrium, phosphorsaures Natron, schwefelsaures Alkali): 1,256 pCt. — Rückstand der Pflanzenfasern, Epithelien und Eiweiss: 1,836 pCt.

Als *abnorm* stellt sich auch hier heraus der *grosse Fettgehalt im Verhältniss zum Eiweiss*, und der gänzliche *Mangel an Gallensäuren und Gallenpigment*, durch welchen Mangel die weissliche Färbung des Fäcalklumpens bedingt ist. Die Behinderung der Gallenexcretion wurde einmal durch den absoluten Mangel von Gallenspuren, dann aber durch den ungemeinen Fettgehalt des Fäcalklumpens ermittelt, denn bekanntlich geht beim Verschluss des Ductus choledochus (oder, wie das Experiment an Hunden gelehrt hat, bei künstlicher Ableitung der Galle durch eine Fistel) das Fett unverändert und ohne Gewichtsverlust durch den Darmcanal. Welcher Art das Hinderniss der Gallenausfuhr in diesem Falle gewesen sei (ob Gallensteine?), konnte nicht ermittelt werden; ich habe mich später bei sorgfältiger Untersuchung der Gallenwege in ähnlichen Fällen überzeugt, dass selbst die Autopsie die Erklärung eines temporären Hindernisses in den Gallenwegen nicht immer zur völligen Befriedigung liefert. Die leichteste von den denkbaren Möglichkeiten, die katarrhalische Schwellung der Schleimhaut, schwillt an der Leiche derart ab, dass die Sonde kein namhaftes Hinderniss findet. Der schräge Verlauf, den der Duct. choled. bei einer Länge von 1 Centim. und darüber durch die Muskelschichten des Duodenum nimmt, mag hiebei, wie bei den Ureteren auf ihrem Wege durch die Blasenwandungen, berücksichtigt werden und zuweilen über die Art des Hindernisses einigen Aufschluss geben, wofern man auf eine Texturveränderung oder auf eine Functionsanomalie der dem Ductus zunächst liegenden Organtheile Bedacht zu nehmen nicht versäumte. Andererseits ist es gewiss übertrieben, die Veränderungen bei einem Duodenal-Katarrh sich so vergänglich vorzustellen, dass sie an der Leiche auch ganz spurlos verschwänden. Die Infiltration des submucösen Zellstoffs bietet allerdings dem harten sondirenden Instrumente nicht diejenige Resistenz, die den festen Verschluss eines Hohlganges erklären soll, die aber zur Retention von Flüssigkeiten gar nicht postulirt wird; eine Abschwellung jedoch, die an der Leiche zu Stande gekommen, wird hier, wie beim Oedem der Epiglottis und an anderen Stellen, eine Faltung zurücklassen, die in Anbetracht sonstiger simultaner Erscheinungen als Symptom des Katarrhs unverkennbar ist, gleich wie das Erysipel der allgemeinen Decken, Scarlatina und Morbilli an der Leiche nicht ganz unkenntliche Spuren zurücklassen. — Da es aber unstreitig Fälle von Ikterus gibt, die weder von einem Duodenalkatarrh, noch von einem sonstigen Hin-

dernisse in den Gallenwegen, somit überhaupt nicht von einer Gallenstauung abzuleiten sind, so entsteht die Frage, *ob die ikterische Färbung immer und nothwendiger Weise dem Gallenpigment zuzuschreiben sei*, und ob die Galle dasjenige sei, was nach diesen Vorstellungen in Folge von Retention und Resorption vom Leberparenchym aus in die Circulation gelangt und einestheils die Gewebe gelb tingirt, anderen Theils die verschiedenen nervösen Zufälle und Functionsstörungen bedingt. Es gibt viele Vorgänge im Blute mit acutem febrilem Verlauf, die mit Ikterus auftreten, jedoch mit der Gallensecretion und Excretion nichts gemein haben. Bei den Infectionen des Blutes (Septhaemie und Ichorhaemie), bei Metastasen und Intoxicationen (Schlangenbiss) steht die ikterische Färbung und die typhoide Allgemeinerkrankung weder mit einer parenchymatösen Veränderuug der Drüsenelemente der Leber, noch mit einer Gallenstauung in irgend einer constanten Beziehung; bei dem gelben miasmatischen Fieber der tropischen Gegenden und bei dem Puerperalfieber ist dies wohl ähnlich der Fall. Unter den localen Vorgängen im Organismus kommt der Lungengangrän und der sphacelösen Splenitis in manchen Fällen unzweifelhaft dieselbe Gruppe von Erscheinungen zu, die mit Alterationen der Gallenorgane nichts zu thun haben und wobei der Ikterus von der Galle unabhängig ist. In einem Falle, wo zahlreiche sphacelöse Entzündungsheerde in der Milz den einzigen Befund an der Leiche eines ikterischen, unter typhoiden Erscheinungen verstorbenen Individuums bieten, wird das Blut mit dissoluten Blutkörperchen, welche den Farbstoff an das Blutserum abgegeben haben, hinreichend versehen werden, um die farblosen Gewebe in wahrnehmbarer Weise gelb zu färben. Die Gallenbereitung in den Parenchymzellen der Leber und die Fortleitung der bereiteten Galle ist dabei nicht behindert; in wie weit jedoch der Einfluss, den das vom Pfortaderblut gelieferte Plasma auf die metabolische Kraft der Leberzellen und auf die Functionsenergie eines ganzen Acinus nimmt, durch den localen Vorgang im Milzparenchym und durch die abnorme Constitution des Milzvenenblutes modificirt werde, ist eine Frage, die uns auf das Gebiet der Pathologie der Leber und der hiebei wahrzunehmenden Anomalien der Darmfunction führt. Hierüber bei einer anderen Gelegenheit.

Nachweis zu den Abbildungen.

(Wo die Vergrösserungsziffer nicht eigens angegeben ist, gilt die 360mal. Vg. Amici.)

Taf. I. Fig. 1. *Normalbefund des Darmexcretes* S. 3, 35. *a—f* Holzfasern, Trümmer, elastische Fasern, Amylonkörner, Zellengewebe und *y* verholzte Pflanzenzellen mit Tüpfelcanälchen; *r r* vergilbte Muskelfragmente (Bowman's Discs); — *m*, *n* gelbe Fetttropfen mit krystallisirender Erstarrung zu Margarin und *o* zu Stearinkörnern (bei Diarrhoea ablactatorum, sauere Reaction).

Fig. 2. *Schleim.* S. 8, 10. Mucinmetamorphose, gelatinöse Quellung und Colloidauflösung von Formelementen in gallertigen Schleimklumpen.
z, *Cercomonas intestinalis*, Vg. 640, S. 51.

Taf. II. Fig. 3. *Schleim.* Vg. 80. S. 8. (Aehnlich bei unvollständiger Verdauung von Amylaceen).

Fig. 4. *Follicular-Blennorrhoe*, S. 36, 51. — *e* und *f* Epithelzellen, Vg. 1020. Trichterform, die Krempe des Basalsaumes z. Th. abgebrochen, keine Poren. (*Cf.* Fig. 8.)

Fig. 5. *Dysenterie.* S. 5, 10, 17, 18, 51. — Gerinnfähiger Erguss mit Eiterzellen.

Fig. 6. *Typhus.* S. 17, 29. *a* Haematinfleck (dunkelroth), *b*, *c* Schrumpfung, *d* hydropische Aufblähung von Blutzellen; *e*, *f* Zellenproduction aus den Follikeln und Mesenterialdrüsen.

Taf. III. Fig. 7. *Cholera* (Vg. 180) S. 6, 15, 23.

Fig. 8. *A.* Verfettung des Epithels, Vg. 80. S. 11. — *B.* Nekrose, *C.* Fettresorption in Epithelialzellen Vg. 640. S. 12. — *D.* obere Flächenansicht des Epithels bei Einstellung des Focus auf den Basalsaum, — *E*, dasselbe bei tiefer Einstellung. Vg. 640.

Fig. 9. *Abgegangenes Gewebstück.* S. 39. *A* und *B* in nat. Grösse, *C.* Vg. 180 Plössl, Fettgewebe mit Trübung des Zellinhalts wie bei Adipocire.

Fig. 10. zur Seite 37. (Siehe Taf. IV. Fig. 10.)

Fig. 11, *A.* Ei von *Ascaris* S. 43, — *H. Distoma* S. 49, *a* in nat. Grösse, *b*, *b* Eier von demselben Vg. 360.

Taf. IV. Fig. 10. *Schleimhautähnliche Neubildung am Peritonaeum* Vg. 80. S. 37. Hiezu Fig. 10 auf Taf. III.

Fig. 11. *Haken von Cestoden: F. Echinococcus*, *G. Cysticercus* S. 44. — Fig. 11, *B.* Ei von *Ascaris* S. 43. — *C.* von *Trichocephalus* (braun), — *D.* von *Oxyuris*, — *E*, *E* von *Taenia Solium.*

Fig. 12. *Echinorrhynchus* S. 45. *A.* Vg. 20, *B.* u. *C.* Vg. 80. — *D.* Eier bei starker Vergrösserung nach V. Carus.

Fig. 13. *Puccinia* (?) S. 52.

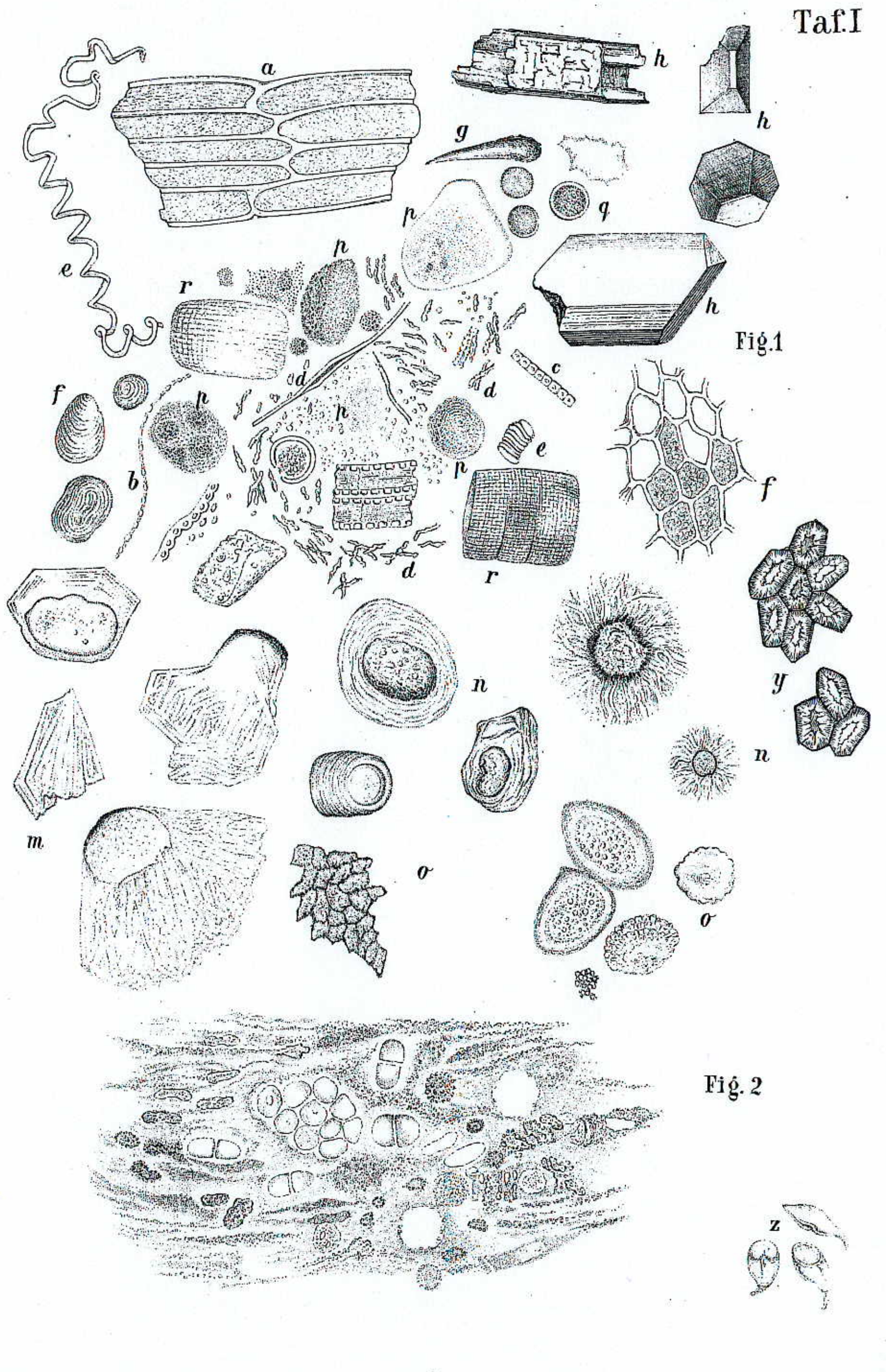

Gez. v. Dr. Lambl. Verlag v. K. Andre in Prag. Steindr. v. J. Habel, Prag.

Taf. II

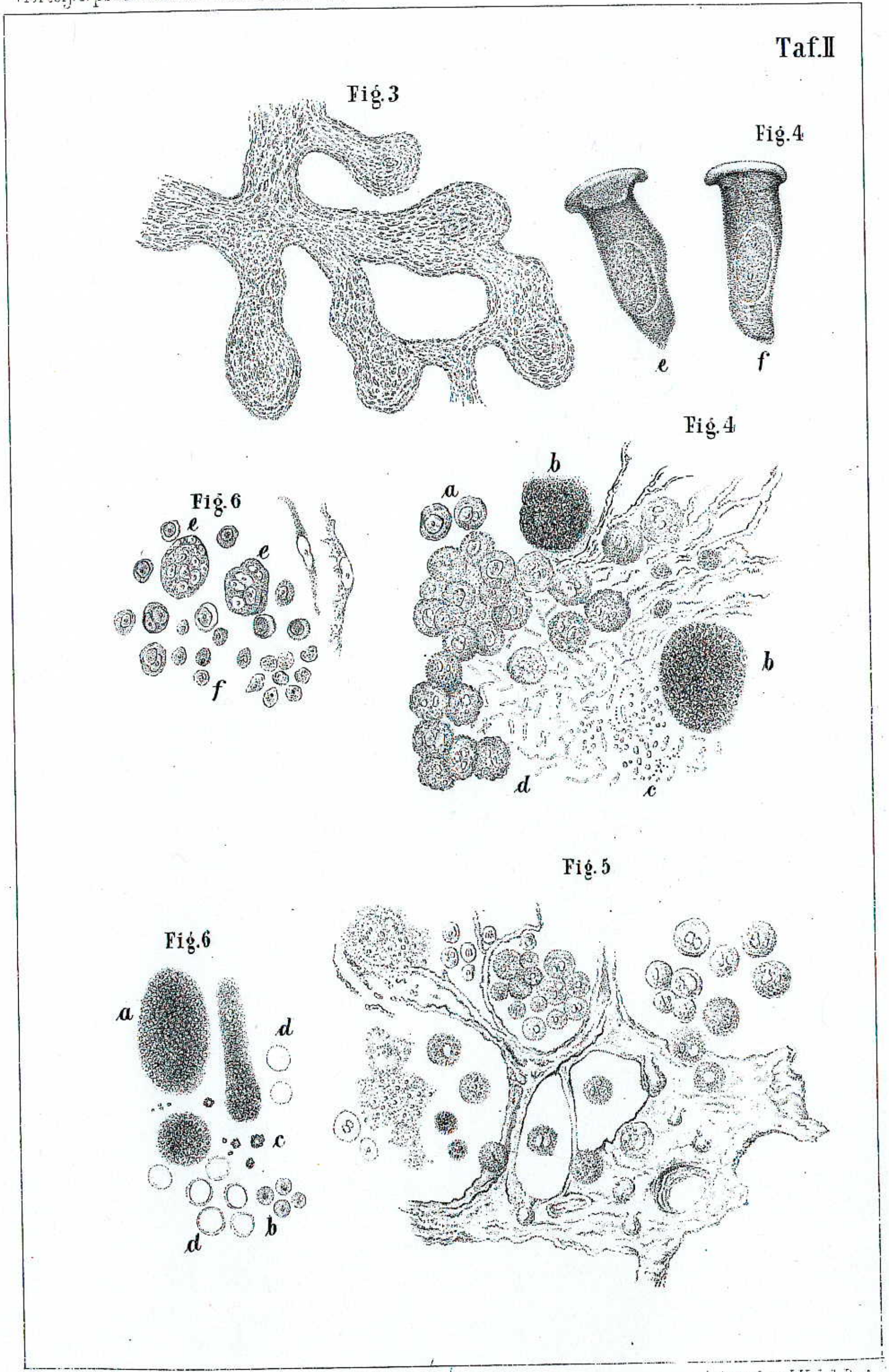

Gez. v. Dr. Lambl. Verlag v. K. Andre in Prag. Steindr. v. J. [illegible], Prag.

Taf. III

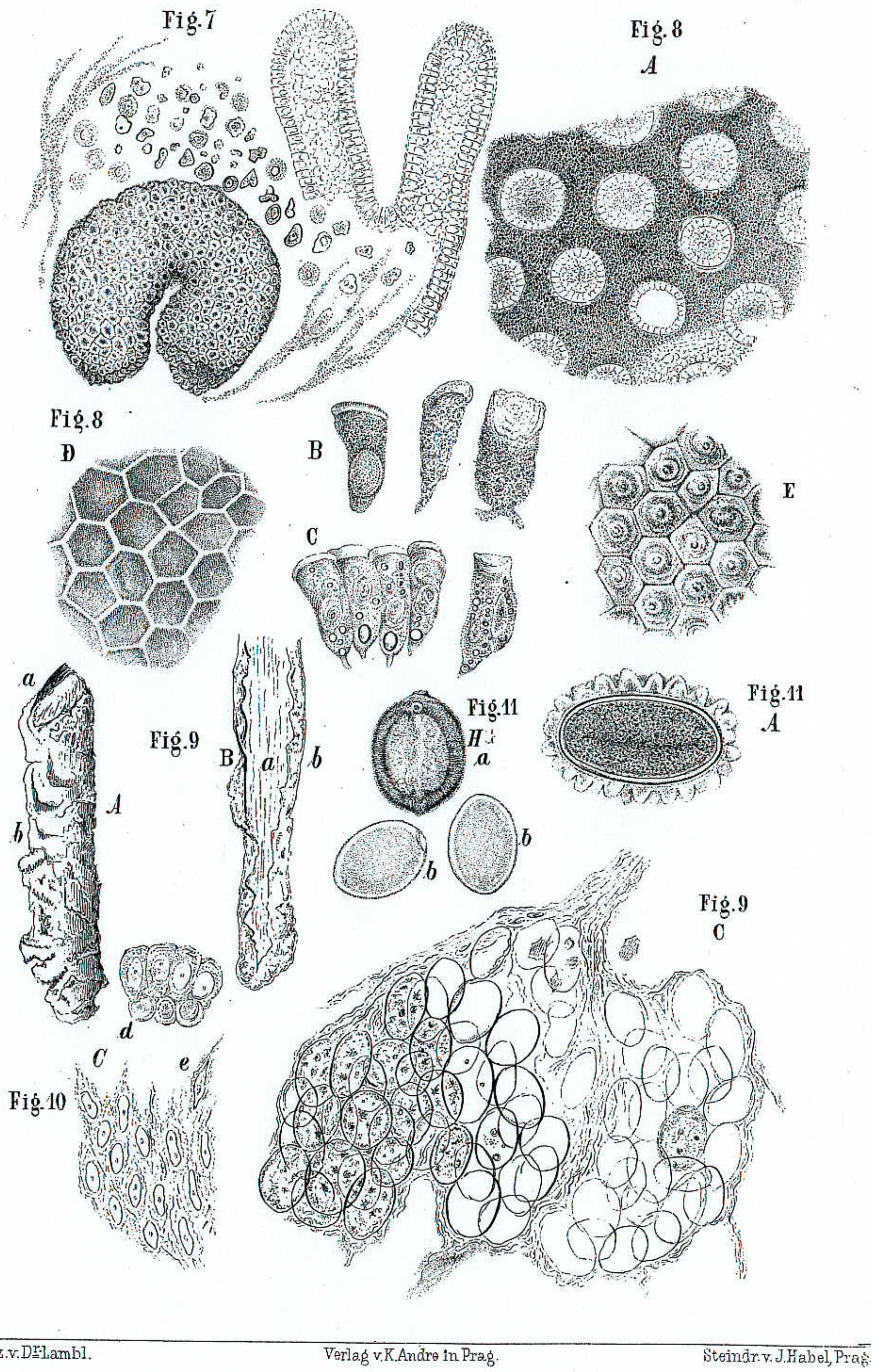

Gez. v. Dr. Lambl. Verlag v. K. Andre in Prag. Steindr. v. J. Habel, Prag.

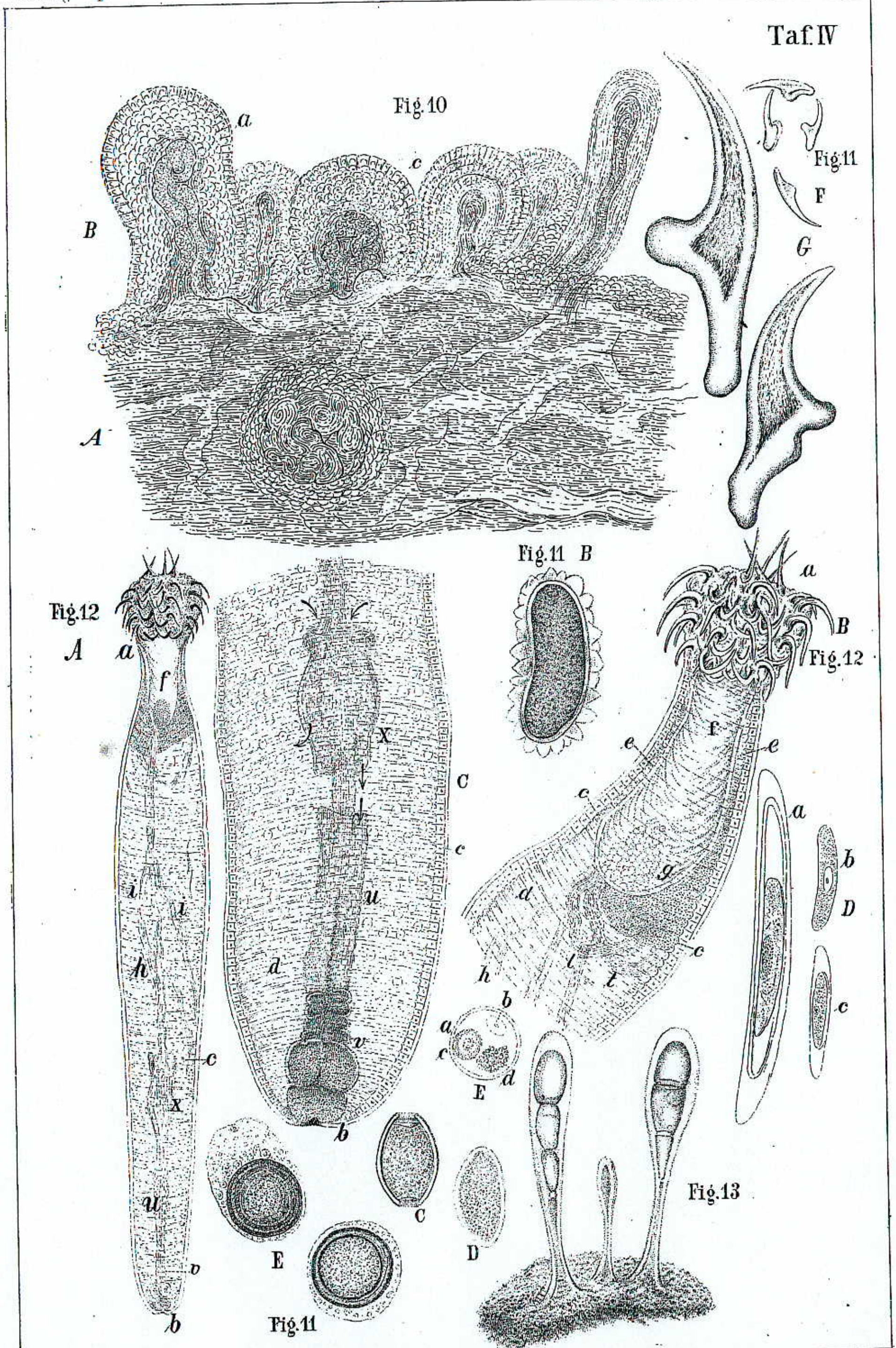

Gez. v. Dr. Lambl. Verlag v. K. Andre in Prag. Steindr. v. J. Habel, Prag.
