# Supplementary material for: Giardia and Vilém Dušan Lambl
Source: PLoS Negl Trop Dis. 2014 May 8;8(5):e2686. doi: 10.1371/journal.pntd.0002686 (PMC4014406; doi:10.1371/journal.pntd.0002686)
Supplement: Text S2 — Article with the first description of Lambl's excrescences. Full text of [13]. (PDF) [file pntd.0002686.s002.pdf]

MVS 89|13

über der Aftermündung, ein spitzes, leicht gebauchtes Skalpell durch alle Weichtheile bis in die Furche, schiebt die Spitze des Messers etwa ½ Zoll in der Sondenfurche nach abwärts und gegen die Blase hin, und schneidet dann, den Griff desselben senkend, in der Mittellinie des Mittelfleisches aus, eine Hautwunde von mehr als Zolllänge anlegend. Das Skalpell wird nun bei Seite gelegt, die Spitze des Zeigefingers der linken Hand geht in die Wunde ein, der freie Nagelrand sucht die Furche der Leitsonde und wird auf deren rechtseitigem Rande eingesetzt, derart, dass die freie Nagelfläche der Mittellinie des Körpers zusieht. Während der linke Zeigefinger die beschriebene Stellung behauptet, ergreift die rechte Hand das lange, schmale, im vorderen Drittel schneidige, geknöpfte Steinmesser, und führt es, von dem Nagel des linken Zeigefingers geleitet, mit seinem Knöpfchen in die Furche der Sonde. Der linke Zeigefinger verlässt sofort die Wunde, und der Operateur ergreift mit seiner linken Hand den Griff der Leitsonde aus der Hand des Gehilfen, senkt selben etwas, als wollte er den Schnabel tiefer in die Blase führen, hebt aber zugleich den Griff gegen sich, um den häutigen und prostatischen Theil der Harnröhre dem Schambogen zu nähern, und schiebt das Knopfmesser parallel mit der Achse des Stammes des Kranken auf der Furche in die Blase. Der Widerstand der Prostata ist leicht überwunden, dieselbe wird beim Einschieben des Messers, und mehr noch beim leicht drückenden Herausziehen desselben in der Mittellinie eingeschnitten, worauf der in der Blase enthaltene Harn durch die Wunde sich entleert. Ist das Knopfmesser aus der Wunde zurückgezogen, so führt man den linken Zeigefinger durch die Wunde unterhalb der Leitsonde in die Blase; dringt selber mit ziemlicher Leichtigkeit zwischen Sonde und Prostata ein, so wird die Furchensonde ausgezogen, die Spitze des Zeigefingers untersucht die Lagerung und Grösse des Steines in der Blase, der Zeigefinger wird dann so gestellt, dass seine Volarfläche nach oben sieht, und die geschlossene Zange wird auf der Volarfläche in die Blase geschoben; dort angelangt wird sie nach Entfernung des Zeigefingers weit geöffnet, der Stein gefasst und in stetigen oder leicht wankenden Zügen ausgezogen.

Wenn wir den ersten Akt dieser Methode, nämlich die Eröffnung der Harnröhre und Durchschneidung der Weichtheile des Dammes, näher betrachten, so fällt der hohe Einstichspunkt auf, welcher den schwammigen Theil der Harnröhre vor der Harnröhrenzwiebel trifft, so dass letzterer nothwendiger Weise in 2 Hälften gespalten wird. Arabische Aerzte pflegen den Einstichspunkt noch näher der hinteren Wand des Hodensackes zu wählen, weil man daselbst die Sondenfurche ganz deutlich durchzufühlen im Stande ist und nie Gefahr läuft, selbe beim Einstiche zu verfehlen. Indem sie beim Ausschneiden die Weichtheile des Dammes nur in der Länge eines halben Zolles durchtrennen, und durch diese kleine, wenig blutende Wunde die Sondenfurche mit der Spitze des Zeigefingers aufsuchen, geben sie diesem ersten Akte der Operation ein sehr elegantes Aussehen. Das nachgeführte langschneidige Knopfmesser durchtrennt dann in einem Zuge die Harnröhrenzwiebel, den häutigen und prostatischen Theil der Harnröhre und die Weichtheile des Dammes bis auf einen Zoll von der Aftermündung entfernt. Die Verletzung des Mastdarmes wird allerdings durch dieses Verfahren erschwert, aber es bleibt oft lange ein Fistelgang hinter dem Hodensacke zurück. — Kehren wir nach dieser Abschweifung zur Thatsache der unausweichlichen Durchschneidung der Harnröhrenzwiebel zurück. Man betrachtet die Verletzung dieses Theiles der Harnröhre in Europa der folgenden Blutung halber als bedenklich, und die üblichen Methoden sind auf Schonung dieses Theiles berechnet. Bei den 50 nach der beschriebenen Methode auf meiner Klinik operirten Fällen war die Blutung während der Operation meist mässig, und Nachblutung kam nur bei drei Individuen vor; bei einem dieser Kranken wiederholte sie sich am 3. Tage; bei allen aber reichten kühle Ueberschläge und Ruhe zur Blutstillung hin und sämmtliche drei Kranke genasen. Es kann aus der Seltenheit der Blutungen nach dieser Methode, wie ich selbe beobachtet habe, übrigens nur geschlossen werden, dass die Durchschneidung der Harnröhrenzwiebel in Egypten unbedenklich ist. Mein verehrter Lehrer und theurer Freund, Prof. Dr. Schuh, hat in Wien drei Fälle nach dieser Methode operirt und 2mal heftige Blutungen erlebt. In Egypten gehören aber Blutungen nach Operationen überhaupt zu den grössten Seltenheiten. Ich habe noch keinen Fall von Nachblutung nach Amputationen hier gesehen. Der einzige Fall von Nachblutung, der aber auch tödtlich ablief, kam nach Unterbindung einer Zungenhälfte wegen Epithelialkrebs am 9. Tage nach der Unterbindung zur Nachtzeit vor. Bei der Sorglosigkeit der Araber würden die meisten Fälle von Nachblutungen tödtlich ablaufen, wenn nicht der Allvater für diese seine Auserwählten so gnädig gesorgt hätte. Wenn ich aus der Seltenheit der Blutungen auf Fibrinreichthum, und aus der Blässe der Haut und der Häufigkeit hochgradig entwickelter, selbstständiger Anaemien auf Blutkugelarmuth bei den Arabern Egyptens schliesse, so bedarf eine solche Vermuthung des Nachweises der Blutanalyse, welche Aufgabe ich Anderen überlassen will. — Es muss zu dem ersten Akte dieser Operation noch bemerkt werden, dass bei Kindern und fetten Erwachsenen dem Einstiche in die Harnröhre das Einschneiden der oberflächlichen Weichtheile des Mittelfleisches vorausgeschickt werden muss, da das Treffen der Harnröhre durch alle Weichtheile hindurch unter solchen Verhältnissen unsicher wird.

In Bezug des zweiten Aktes dieser Operation ziehe ich das kurzschneidige Knopfmesser dem langschneidigen vor, weil ich durch den Gebrauch des ersteren die Gefahr der Verletzung des Mastdarmes vermindert glaube. Was mir aber als das Wichtigste bei diesem Akte erscheint, ist, den Einschnitt in die Prostata, mit Ausnahme des Vorhandenseins ausserordentlich grosser Steine, klein zu machen; zwischen 1—2 Linien Tiefe genügen selbst bei Steinen, welche 3—4 Unzen wiegen. Da die Dicke der Prostata vor der Operation nur undeutlich erkannt werden kann, so setzt ein ausgiebiger Einschnitt der Durchschneidung der fibrösen Hülle der Prostata aus, wodurch dem Kranken die grösste aller Gefahren erwächst, nämlich die einer Harn- und Eiterversenkung ins Becken. Ausserdem kömmt man bei einem ausgiebigen Schnitte in das Prostatagewebe beim Herausführen des Messers in grössere Nähe des Mastdarmes, und schneidet ihn leichter ein. Ich habe jene Fälle am glücklichsten verlaufen gesehen, wo bei kleinen oder mittelgrossen Steinen die Prostata kaum eingeschnitten wurde, wie diess die arabischen Aerzte meist zu thun pflegen, welche sich nach Zurückziehung des Knopfmessers mit dem Zeigefinger durch den Blasenhals drängen und die Zange nachführen. Bei meinen 50 medianen Steinschnitten wurde der Mastdarm 5mal verletzt; bei dem einen dieser Fälle war der ungewöhnlichen Grösse des Steines halber die Verletzung absichtlich geschehen. Von den andern Fällen wurde 1 bei einem Kinde ohne Zuthun der Kunst geheilt, bei den übrigen 3 blieben mehr weniger umfangreiche Fisteln zurück. Es kommt somit auf 10 Operirte eine Verletzung des Mastdarmes, was nicht gegen die Methode spricht. (Schluss folgt.)

Papilläre Excrescenzen an der Semilunar-Klappe der Aorta.

Vom Assistenzarzte **Dr. Lambl** in Prag.

(Mit zwei Abbildungen.)

Von diesen Excrescenzen findet man bisher nirgends eine Angabe, weder eine Beschreibung in den anatomischen Büchern noch eine Abbildung in Tafelwerken. Sie wurden wahrscheinlich wegen ihrer ungemeinen Zartheit übersehen, und sind daher auch nicht Gegenstand einer mikroskopischen Untersuchung geworden.

Mit dem blossen Auge nimmt man sie bei besonderer Aufmerksamkeit nur im Wasser, wo sie frei flottiren, wahr, während sie sonst der Klappenfläche anliegend unbemerkt bleiben. — Es sind feinfädige Gebilde von 0,040—0,080 Lin. Dicke und ¼ bis 1½ L. Länge, die theils einzeln, theils zu Büscheln oder Pinseln vereinigt, zuweilen von einem gemeinschaftlichen Stiel ausgehend, zuweilen einem zarten Leistchen in einer Reihe entwachsen erscheinen.

Sie sitzen auf der dem Ventrikel zugekehrten Fläche am häufigsten an den halbmondförmigen Leistchen, selten am freien Rande, immer aber in der Nähe des nodulus Arantii, zuweilen neben, bald unterhalb, bald auf demselben, und zwar vorzugsweise am hinteren Zipfel der Aortenklappe, seltener auch am linken und rechten Zipfel. Sie haften wie feine eingefilzte Seidenfäden so innig an der Klappe, dass es einer gewissen Gewalt bedarf, um sie mittelst einer Pincette loszureissen. Es bedarf keiner weitläufigen Deduction, dass und wie sie von den sogen. Vegetationen (Fibringerinnungen) zu unterscheiden seien; sie haben mit den Schollen amorpher Masse

nicht einmal die äussere Formähnlichkeit gemein, und sind vielmehr organische, nach dem Typus des physiologischen Bildungsvorganges zur Entwicklung kommende Gebilde, die ohne Zweifel einen langwierigen Bestand haben. und nur unter Umständen zu einer pathologischen Bedeutung gelangen.

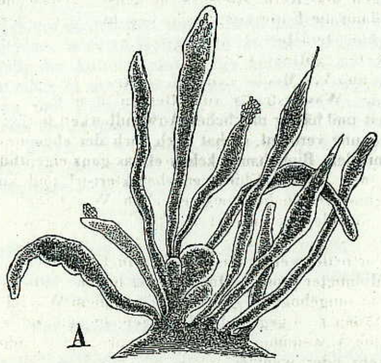

A

Papillar-Excrescenz an der Aorten-Klappe (Vgr. 68 Plössl).

Ihr mikroskopisches Bild (*A*) lässt sich am passendsten mit den gefässlosen Ansätzen an den Synovialzotten vergleichen: cactusähnliche Formen, die man bald kolbig, bandartig und fädig — bald zottig, papillär, zungenförmig — bald einfach, bald verzweigt — immer aber mit den reinsten, schärfsten Contouren gezeichnet findet, und sie ohne besondere Präparation, ohne Behandlung mit Reagentien bis ins elementäre Detail untersuchen kann.

Sie bestehen in ihren jüngsten Formen aus soliden Anhäufungen rundlicher Zellen von 0,004—0,006''' Durchmesser, die in der tieferen Epithelschichte aus den normalen entstehen, und sich durch Spaltung ihrer Kerne vermehren. — Die Zellen identifiziren sich später zum Theil zu einem bald homogenen, bald aber fibrillär gestreiften, gegen Essigsäure resistenten soliden Strange, der als Centralaxe den ganzen Körper der Excrescenz durchzieht und im Allgemeinen opak ist; — zum Theile jedoch reiben sich die Zellen als eine einfache oder mehrschichtige Epithellage an der Peripherie des kompakten Axenzylinders an, und werden den normalen Epithelzellen des Endocards gleich.

Der solide Centralkörper steht mit der Bindegewebsschichte der Klappe in innigem Zusammenhange, so dass es in einigen Excrescenzen oft das Ansehen hat, als wäre der spiralig gewundene Strang vom Bindegewebe aus mit Vorwölbung der Epithelschichte ausgewachsen; derselbe ist meistentheils von körnigem Pigment gebräunt, u. z. bald in Form einer diffusen Punktmasse, bald aber in regelmässig geordneten der Zellenanordnung entsprechenden Körnerhaufen, die sich nachweisbar innerhalb der Zellenkerne ablagern. — Colloidkugeln und Kreidekörner gehören zu den Seltenheiten, und kommen nur in beträchtlich dicken Stämmen der Excrescenz vor.

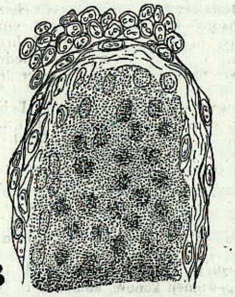

B

Die Spitze einer solchen Excrescenz (Vgr. 360 Plössl).

In der Regel ist der solide Centralcylinder beiderseits von einem hellen Saum (d. i. an seiner Peripherie von einer homogenen hyalinen Substanz) umgeben, und nach aussen von diesem mit einem Epithel von in regelmässigen Abständen aufgetragenen spindelförmigen Zellen (Länge: 0,008—0,012 Lin., Breite: 0,004''') begränzt, worunter zweikernige Zellen nicht selten zu sehen sind. — Der terminale Trieb einer solchen Excrescenz ist zuweilen mit einem kurzen, konisch geformten, oder aber mit einem bandähnlich abgeflachten und selbst fadenförmig auslaufenden Fortsatz ohne Zellenbeleg versehen; meistentheils jedoch ist

die zungenförmig abgeflachte Spitze mit einer dichten Zellenmasse gekrönt, deren Elemente den oben erwähnten noch nicht entschieden differenzirten Bildungszellen entsprechen (*B*).

Während sich sonach die Excrescenz durch diese Zellenproduktion an der Spize vorwaltend in die Länge entwickelt, nimmt sie wohl auch durch einen ähnlichen Vorgang an Dicke zu. Nicht selten verzweigt sie sich auch, und die einzelnen Zweige wachsen sofort wie die einfache Excrescenz aus. Bei Zunahme des Dickendurchmessers verschmelzen zuweilen nicht blos die Zweige, sondern auch ursprünglich einfache isolirte Papillen vom Grunde aus, so dass dann die büschelförmige Excrescenz von einem verhältnissmässig dicken Stiel getragen wird. — Gefässe findet man darin nicht, denn die Klappe selbst ist gefässlos; ein täuschendes Bild gewahrt man nicht selten in solchen Excrescenzen, wo ein doppelter scharf markirter Centralcylinder vorkömmt, eine seltene Erscheinung, die übrigens aus dem über die Entwickelung bemerkten leicht zu erklären ist.

Diese Excrescenzen sind an keine bestimmte Erkrankung des Endocards gebunden. Sie kommen wohl in Combination mit dem atheromatösen Prozess an der Aorta, aber auch unabhängig von demselben, oft an ganz gesunden Herzen, in ihren Anfängen bereits vor dem 30. Lebensjahre vor; Spuren von Endocarditis mögen gleichwohl nur als zufällige Combination gefunden werden. Allerdings betrifft die Mehrzahl der von mir verzeichneten Fälle das Alter von 50—60 J. und darüber, indessen kamen die schönsten, zartesten Villositäten bei dem Alter unter 40 J., namentlich exquisite Exemplare bei weiblichen Leichen von 27 und 30 Jahren vor. — Unter nahezu 1000 Leichen hatte ich seit einem Jahre 20 Fälle von papillären Excrescenzen mikroskopisch untersucht und gezeichnet; ich würde das Vorkommniss überhaupt auf 2% einer Leichensumme schätzen, was mir eher zu wenig scheint als zu viel. Diese Prozentangabe betrifft die exquisiten leicht bemerkbaren Fälle von papillären Excrescenzen; die mit unbewaffnetem Auge nicht wahrnehmbaren Zellenwucherungen — die ersten Anfänge der Excrescenz an der Klappe — sind ungemein häufig, und werden hier nicht gerechnet.

Im ausgewachsenen Zustande verharren die Excrescenzen wie es scheint lange Zeit unverändert, oft indifferent gegen die Vorgänge im Cirkulationsapparat, und eben so ohne erheblichen Einfluss auf die organ. Vorgänge. Indessen gibt es Fälle, wo sie eine beachtenswerthe Rolle spielen. In manchen Fällen fand ich die terminale Zellenwucherung sehr lose haftend; dieselbe löste sich oft in rundlichen Klümpchen dicht zusammen geballter Zellen — deren Anzahl ich an 12—20 schätzen konnte — ab; aber solche freie Zellenmassen lagerten oft, gleichsam in Nestern, zwischen den Zöttchen und in den Spalten der Bifurcationen. — Zu dickeren Büscheln gesammelt werden sie nebstdem manchmal von Fibringerinnungen umhüllt, und geben gleichsam das Gerüst für die lockeren Niederschläge ab, die sich als globuläre, warzige, knotige Massen — die „Vegetationen" der Autoren — reichlich anlagern. In diesen findet man Fibrinschollen und Klumpen einer amorphen, diffus pigmentirten Masse, Blutkörperchen, Detritus und Zerfall der Epithelzellen, freie Kerne und Pigmentmolecüle. Der Zerfall der Excrescenzen ist sonach in der Regel ein secundär durch die eingefilzten amorphen Fibrinschollen in Folge von Usur herbeigeführter Zustand, den man an reinen Excrescenzen nicht beobachtet.

Es liegt nahe, diesen Umstand mit Virchow's Embolie in Verbindung zu bringen, da es eben so a priori leicht begreiflich erscheint, dass die ungeordneten Zellenhaufen als kompakte Klümpchen und fädige Flocken, so wie die morsch gewordenen Fibrinschollen mit der Zeit in grossen Partikeln vom Blutstrom losgewühlt werden, und an entfernten Partien in Capillargefässen in Stockung gerathen, — als es in solchen Fällen thatsächlich verschiedene Entzündungsvorgänge entlegener Organe (Encephalitis, Meningitis, Pneumonie etc.) sind, die zu dieser Idee führen. — So schwer es hält, über die physiologische Bestimmung der Excrescenzen eine Vermuthung auszusprechen, so wenig wird über ihre pathologische, unter Umständen sehr wichtige Bedeutung gezweifelt werden können. Sesshaft im Centrum des Cirkulationsorganes, können sie ungeachtet ihres mikroskopischen Verhaltens zu ungewöhnlichen Vorgängen in den Blutbahnen, zu Störungen im Capillarsystem, zu Ernährungsanomalien an irgend einem Punkte der Peripherie Veran-

*

lassung geben, und so zum Ausgangsheerde grossartiger, selbst lebensgefährlicher Veränderungen im Organismus werden. Der direkte Nachweis des ursächlichen Zusammenhanges dieser Prozesse mit der Klappenexcrescenz gehört gleichwohl zu den grössten Schwierigkeiten der Anatomie, wenn auch nicht zu den Unmöglichkeiten.

Indem wir es uns vorbehalten, unsere Erfahrungen über diesen Gegenstand bei einer anderen Gelegenheit im Detail mitzutheilen, leiten wir vorläufig die Aufmerksamkeit der Anatomen auf diesen Punkt hin; man möge es uns nicht verargen, wenn wir meinen, die Leichenöffnungen und Untersuchungen würden fruchtbringender sein, wenn sie sich die Aufgabe stellen, die verschiedenen Zustände der Organe, d. i. Entwickelungsphasen innerhalb physiologischer Gränzen, zur Evidenz zu bringen, — als wenn man blos secirt, um die Diagnose einer Krankheit zu bestätigen.

Offenes Schreiben an Herrn Professor Schuh.

Ueber die Methode des Prof. Vanzetti, fremde Körper aus dem Ohr zu entfernen.

Von **Prof. Brunetti** in Padua.

Sie dürfen nicht glauben, dass mich die pathologische Anatomie der Chirurgie gänzlich entfremdet; ich benütze im Gegentheil sich mir darbietenden Anlass, was in Folge der Freundlichkeit meines wackern Kollegen, Prof. Vanzetti, oft der Fall ist, um eine Kunst zu üben, für die Sie, mein gütiger Lehrer, mir so viel Liebe einflössten; dass dem so ist, möge Ihnen die Eile beweisen, mit der ich Ihnen nachstehendes Faktum erzähle.

Die Ausziehung fremder, zufällig in den äussern Gehörkanal gerathener oder absichtlich dorthin gebrachter Körper dürfte von vorn herein Manchem als etwas ganz Unerhebliches erscheinen, und doch kommen derartige Fälle vor, die nicht nur den gewöhnlichen, sondern auch einen besonders gewandten Chirurgen in Verlegenheit setzen können.

Ich weiss nicht, ob Prof. Vanzetti's Methode auch schon von Andern in Anwendung gebracht worden ist; ich habe ihre Ausübung nur von ihm gesehen.

Sowohl bei uns als überall, wo die Frucht des Johannisbrotbaumes genossen wird, sind die Kerne dieser namentlich von Kindern gern genossenen Speise diejenigen Körper, deren Entfernung aus dem Ohre der Chirurg sehr häufig vorzunehmen hat. Die Methode, die nun V. hiebei und bei der Entfernung jedes andern fremden Körpers in Anwendung zu bringen pflegt, ist überaus einfach. Ein Wasserstrahl wird mittelst eines guten Spritzchens kräftig gegen eine jener Seiten getrieben, wo der Kern weniger eingekeilt zu sein scheint; sobald nun das Wasser hinter den Kern gelangt, lässt sich dieser mit grösster Leichtigkeit ausziehen. Im letztverflossenen Sommer hatte ich Gelegenheit mich von der Zweckmässigkeit dieser Methode zu überzeugen, und über den glücklichen Ausgang in wahres Erstaunen zu gerathen.

Heute ist auf unsere Klinik ein 45jähriger Bauer gekommen, der seit 8 Tagen im linken Ohr einen Johannisbrotkern stecken hatte; mehrere Chirurgen hatten die Entfernung des fremden Körpers bereits mit allen erdenklichen Mitteln, aber vergebens versucht gehabt. Prof. V. nahm sofort Zuflucht zu seiner gewöhnlichen Methode. Der Kern war quer gelagert und in Berührung mit dem Trommelfell stehend; es hatte den Anschein, dass man leichter zum Ziele gelangen würde, wenn man den Wasserstrahl von unten nach oben richtete. Der Kern wurde in der That ein wenig aus seiner Lage gebracht; da diese jedoch noch immer eine quere blieb, und weil die den Gehörgang auskleidende Membran bedeutend angeschwollen war, blieben die weitern, zu wiederholten Malen vorgenommenen Versuche gänzlich fruchtlos.

V. behauptete, er werde mit seiner gewöhnlichen Methode, die sich ihm schon in weit schwierigeren Fällen bewährt hätte, auch diessmal zum erwünschten Ziele gelangen; auf das fortwährende Bitten des Kranken, es denn doch auch mit einem andern Behelfe probiren zu wollen, entschloss er sich auch dazu. Mit jener chirurgischen Ruhe, die ein geistreicher aber nicht sehr galanter Korrespondent dieses Blattes als „russisch" bezeichnen zu müssen glaubte, brachte er den Kern zuerst mit einem Daviel'schen Löffel ein wenig aus seiner Position, nahm dann einen kleinen Haken zur Hand, wie man sich ihrer zur Fixirung der Bindehaut bei Strabismus-Operationen zu bedienen pflegt, führte ihn parallel zwischen dem Kern und der Wand des Gehörkanals ein, wendete sodann die Spitze des Hakens gegen den Kern, stiess sie in denselben ein und bewerkstelligte sodann die Entfernung desselben mit bewundernswerther Geschicklichkeit und Leichtigkeit, ohne dass der Patient im Mindesten Schmerzen gelitten hätte.

Wenn nun V.'s Methode, fremde Körper aus dem Gehörgange mittelst eines Wasserstrahls zu entfernen, ihrer Einfachheit, Unschädlichkeit und häufig möglichen Anwendbarkeit halber die höchste Anerkennung verdient, so hat doch auch der eben beschriebene Vorgang mit dem Bindehauthäkchen etwas ganz eigenthümlich Geniales, das den wahren Chirurgen charakterisirt und mich an die Raschheit und Originalität unseres alten Wattmann erinnert, mit der er für jede Eventualität das passendste Hilfsmittel zu finden wusste.

Die Vortheile, welche das Häkchen bietet, dessen Curve einen nur zwei Millimeter grossen Durchmesser haben, und dessen Spitze nicht zu sehr umgebogen (¿), sondern in einem Winkel von ungefähr 45 Graden (¿) gegen das Heft geneigt sein muss, sind folgende: Seine Anwendung eignet sich für alle jene fremden Körper, die sich mehr oder weniger anhaken lassen, besonders daher für die Körner der Johannesbrotfrucht; in dieser Hinsicht dürfte er in nur wenigen Fällen nicht zur Anwendung angezeigt erscheinen, und es genügt, wenn seine Spitze auch nur ganz oberflächlich in den fremden Körper eindringt. Die Einführung bietet fast gar keine Schwierigkeiten. Die meisten Vortheile ergeben sich, wenn der fremde Körper sich in einer Querlage befindet; wird dann der Haken in eines seiner äussersten Enden eingepflanzt, so nimmt er in dem Maasse, in welchem die Zugkraft ausgeübt wird, rasch die seiner Entfernung günstigste Wendung an, indem er von selbst eine Kreisbewegung um die Hakenspitze beschreibt.

Nach meinem Dafürhalten ist die Anwendung des Hakens in jenen Fällen, in denen der Wasserstrahl zur Entfernung des fremden Körpers nicht ausreicht, so zweckmässig, dass alle andern zu diesem Behufe erfundenen Instrumente überflüssig erscheinen.

Einige Worte zur Vertheidigung der Vorschrift für die Vornahme der gerichtlichen Todtenbeschau vom 29. Jänner 1855.

Von
Dr. Franz Gatscher,
Professor der gerichtlichen Medizin in Lemberg.

(Fortsetzung.)

Es ist Etwas faul im Bereiche des gerichtsärztlichen Unterrichts, das hat Prof. Engel in seiner klaren Anschauung sogleich erkannt und scharf ausgesprochen. Die Möglichkeit aber, dass Jemand in dieser Darlegung des Prof. Engel einen Vorwurf ersehen wollte, welchen er dem hohen Unterrichtsministerium willkührlich und unwillkührlich habe machen wollen, nöthigt mich zur Mittheilung des Folgenden, da ich näher eingeweiht bin.

Bis vor nicht gar langer Zeit war der fragliche Unterricht an allen Chirurgenschulen dem Lehrer eines andern Gegenstandes gegen Entrichtung einer Remuneration überlassen. Der Grundsatz der Auswahl in dieser Uebertragung ist mir nicht bekannt, wenn überhaupt einer bestand; fast möchte man das läugnen, wenn man bemerkt, dass an einer Anstalt der Lehrer der theoretischen Medizin, an einer andern jener der Anatomie, und wieder an einer dritten jener der Veterinärpolizei der gleichzeitige Vertreter der gerichtlichen Medizin ist.

An den medizin. Fakultäten war wohl von jeher ein eigener Lehrer hiefür bestellt, demselben aber die Vertretung eines zweiten vollkommen heterogenen Gegenstandes, der medizinischen Polizei, nebstbei übertragen. Um den Lehrern Materiale zu praktischen Vorträgen zu verschaffen, erfloss die hohe Bestimmung, dass alle im Orte der Lehranstalt vorkommenden gerichtlichen Obductionen in jenem Locale und in jener Stunde vorgenommen werden sollen, in welchen der Professor der gerichtlichen Medizin seine Vorträge hält, damit er mit seinen Schülern denselben beiwohnen könne (Hofkanzleidekret vom 6. August 1812).

So viel mir bekannt, wurde diese gesetzliche Bestimmung, wodurch der Unterricht allein eine praktische Seite gewinnen könnte, analog den klinischen Vorträgen über spezielle Pathologie, nur an der Universität in Prag und den Chirurgenschulen beachtet und befolgt, an der Wiener Hochschule bis in die jüngste Zeit vollkommen ausser Acht gelassen, indem daselbst die
