# Supplementary material for: Giardia and Vilém Dušan Lambl
Source: PLoS Negl Trop Dis. 2014 May 8;8(5):e2686. doi: 10.1371/journal.pntd.0002686 (PMC4014406; doi:10.1371/journal.pntd.0002686)
Supplement: Text S3 — Article with the first description of detection of bladder cancer using the analysis of cells present in urine. Full text of [14]. (PDF) [file pntd.0002686.s003.pdf]

Vierteljahrschrift

für die

PRAKTISCHE HEILKUNDE,

herausgegeben

von der

medicinischen Facultät in Prag.

Redaction:

Dr. **Josef Halla**, Professor der zweiten medicinischen Klinik.
Dr **J. v. Hasner**, a. o. Prof. d. Augenheilkunde. — Dr. **J. Kraft.**

Dreizehnter Jahrgang 1856.

Erster Band

oder

Neunundvierzigster Band der ganzen Folge.

(Mit 4 lithogr. Tafeln.)

PRAG.

Verlag von Karl André.

Original - Aufsätze.

Ueber Harnblasenkrebs.

Ein Beitrag zur mikroskopischen Diagnostik am Krankenbette.

(Mit 4 lithogr. Tafeln.)

Vom Assistenzarzte Dr. Lambl.

(Im Auszug mitgetheilt und zum Theil demonstrirt in der Versammlung des Prager medicinischen Doctoren-Collegiums am 30. November 1854).

Soweit uns die Literatur zugänglich ist, finden wir die Diagnose des Harnblasenkrebses durch mikroskopische Untersuchung der Harnsedimente nirgends gründlich behandelt, oder vielmehr gar nicht verzeichnet. Hat doch der emsige K. Bock, dem die Sache kaum entgangen wäre, in seinem *Lehrbuch der Diagnostik* (Leipzig 1853), keine Erwähnung davon gethan; denn im allgemein-diagnostischen Theil wird der Krebs nach seinen Formen geschildert, wie man sie als Präparate (exstirpirte Geschwülste) untersuchen kann, und hinterher wird der röhrigen Gebilde ganz kurz Erwähnung gethan, mit Bezugnahme auf einige, nicht näher versinnlichte Termini von Engel, Rokitansky und Bruch; — unter den Harnblasenkrankheiten im speciellen Theile werden zwar (S. 291) die morphotischen Bestandtheile des Harns nach Lehmann aufgezählt, und nebstdem S. 547 alle möglichen Symptome, besonders das Blutharnen ausführlich besprochen, letzteres sogar als Blutung aus der Niere, aus der Blase und aus der Urethra genau unterschieden, — eine Unterscheidung, deren Möglichkeit am Krankenbette uns, aufrichtig gesagt, schwer einleuchten will, — der Krebs der Harnblase wird aber dabei nicht genannt, als

existirte so Etwas gar nicht für den Kliniker, was doch für den Anatomen keine grosse Seltenheit ist. — In Funke's Atlas der physiologischen *Chemie* und in Neubauer's Analyse des Harns 1854 darf man schon die organischen, und selbst hochorganisirten Bestandtheile der Harnsedimente vermissen; allein auch in Höfle's Chemie und Mikroskop am Krankenbette (S. 393 und S. 437, wo von den organischen Formelementen die Rede ist), findet sich keine Angabe über diesen, besonders für den praktischen Arzt hochwichtigen Befund.

Das Mikroskop baut zwar eine Wissenschaft auf, die für den Kliniker nicht in allen ihren Details nothwendig ist; wenn aber irgendwo seine Zuhilfenahme dringend geboten wird, so ist es bei der Harnuntersuchung der Fall, wo erst eine sichere Diagnose die einzuschlagende Therapie bestimmt. Während sich der Stein bald hinter der Mercier'schen Klappe, bald in einem Divertikel, bald unter einem Schleimüberzug dem eingeführten Katheter entzieht, kann man — nach der Aussage unseres Gewährsmannes, des Hrn. Prof. Pitha, dessen freundlicher Liberalität wir zum grössten Theil das Materiale zu den folgenden Untersuchungen verdanken, — neben manchen täuschenden Symptomen besonders bei der Einführung des Katheters in die Harnblase oft auf die Vermuthung eines fremden Körpers — vielleicht selbst auf die Annahme von Steinbildung — geführt werden, wo doch die mikroskopische Analyse eines mit dem Harn abgegangenen, schleimähnlichen Flockens unzweideutigen Aufschluss über die eigentliche Erkrankung der Blase zu geben vermag, eines Pseudoplasmas, welches eine eigene Behandlung erheischt, wenigstens jede andere ausschliesst.

Der *erste Fall*, der die Sache ins Licht stellt, wurde uns vom Hrn. Prof. Pitha mitgetheilt. Ein über 50 Jahre alter Beamte litt seit langer Zeit an Haematurie und zeitweiliger Ischurie, wegen welcher H. Prof. Pitha kurze Zeit vor dem Tode des Patienten geholt wurde. Bei der ersten Einführung des Katheters ergab sich an der sonst starrwandig hypertrophirten und contractions-unfähigen Harnblase, neben Vergrösserung der Prostata — weder ein Hinderniss in der Urethra, noch irgend ein anderer erheblicher Zustand der Harnblase, worauf die Blutung hätte bezogen werden können. Als der Katheter zum zweitenmal bei aufrechter Stellung des Patienten eingeführt wurde, schlug in dem Augenblicke, wo die Spitze des Instrumentes in die Blase gelangte, ein weicher Körper dumpf an dieselbe an, konnte aber später nicht wieder aufgefunden und näher bestimmt werden. Nebst Blut fanden sich im Harn lockere Klümpchen, die wie Exsudat-Fetzen oder morsche Gewebstrümmer aussahen. H. Prof. Pitha schloss den früher von den Aerzten vermutheten Stein aus, und stellte die Diagnose vorläufig auf Harnblasenkrebs mit der

Hoffnung, dass die nähere Analyse des Harns einen bestätigenden Aufschluss geben werde. Der Chemiker, der die entleerte Flüssigkeit prüfte, gab an, in den Sedimenten eine Substanz, ähnlich dem geronnenen Faserstoff oder frischem Exsudate gefunden zu haben. Dadurch wurde die Diagnose — zur grossen Beruhigung des Patienten und seiner Angehörigen — schwankend; ihre Richtigkeit erwies sich jedoch, nach dem kurze Zeit darauf erfolgten Tode des Kranken, da die Autopsie der Harnblase gestattet wurde. Die Richtigkeit der Diagnose hätte müssen auch früher bestätigt werden, wenn man die Untersuchung mehr mikroskopisch als mikrochemisch vorgenommen hätte. — Die Harnblase enthielt blutigen Harn und Flocken, nämlich Bruchstücke von dem sogleich zu beschreibenden Neoplasma, die sich als identisch mit jenen erwiesen, die bei Lebzeiten häufig mit dem Harn abgegangen waren. — Fig. 1 zeigt den Umriss der dick- und starrwandigen Harnblase (a a), vorn durchschnitten und mit den beiden Prostata-Hälften (b b) lospräparirt. Im Trigonum erhebt sich mehr nach der rechten Seite hin, von einem dicken Schleimhautwulste (c) ein wallnussgrosser Complex feinfädiger Vegetationen, deren hintere Partie von einem mit dem Mutterboden (Trigonum) zusammenhängenden, $1^1/_2$—2 Linien breiten, über 2 Zoll langen Schleimhautstrang (d) überragt wird, dessen eine Seite glattrandig, die andere mit zartfädigen Vegetationen besetzt, das obere Ende gleichfalls mit einem Büschel der feinflockigen Neubildung gekrönt erscheint. Das letztere flottirt im Wasser frei, kann sich aber nach abwärts dem Strome der Flüssigkeit folgend, tief in die Pars prostatica einkeilen und deren Lumen verlegen. Die Vegetation erweist sich an den losgewühlten Flocken sowohl, als auch an dem bestehenden Präparate als ein Gebilde von dichotomisch, seltener auch trichotomisch verzweigten Schläuchen (Fig. 2. a) von 0,010—0,015''' Breite, an denen bei einer Vergrösserung von 400 eine structurlose streifig-punctirte Membran mit längsovalen Kernen besetzt (b), und mit polymorphen, grösstentheils einkernigen Zellen (c) belegt, zur Ansicht kommt; einfache Blutgefässe durchziehen das Innere der Schläuche oft bis an deren terminale Verzweigungen, ohne dass jedoch ein zu- und ein abführendes Rohr wahrgenommen werden könnte.

Es kann hier und in den folgenden Fällen, nachdem R o k i t a n s k y's klassische Schilderung des Krebsgerüstes, namentlich der dendritischen Vegetation als bekannt vorausgesetzt wird, die detaillirte Beschreibung eines jeden Präparates als überflüssig betrachtet werden, wo hingegen das Ergebniss der klinischen Untersuchung, namentlich der Hergang der mikroskopischen Prüfung des Harns genau erörtert werden soll. — Ueber den ersten Fall glauben wir folgendes bemerken zu müssen, was ein besonderes Interesse bietet:

1. Der Zottenkrebs ist hier an seinem Ausgangspunkte auf eine kleine Fläche der Schleimhaut begränzt, und daher trotz seiner namhaften Grösse, und besonders auch in Anbe-

tracht der Blasen- und Prostata-Hypertrophie als Geschwulst durch den After nicht durchzufühlen gewesen.

2. Das Gewächs wuchert besonders in den terminalen Trieben und Ramificationen, ungefähr nach dem Paradigma der Chorionzotten, im Hintergrunde der starrwandigen Höhle in der Art, dass es sich auch dem Katheter nicht verräth.

3. Der strangförmige, dem Mutterboden des Pseudoplasmas entwachsene Schleimhautfortsatz und dessen quastenförmige terminale Vegetation, erklären bei ihrer Vorlagerung nicht blos eine paradoxe Ischurie ohne ein nachweisbares Hinderniss in der Urethra, sondern auch die Vortäuschung eines (weichen) Concrementes durch dumpfes Anschlagen an die Spitze des Katheters.

4. Das Pseudoplasma tödtete durch unaufhörliche langwierige Blutung und dadurch bedingte Anämie vielmehr, als durch Zerstörung im Bereiche seines Mutterbodens, oder durch Zerfall in Folge innerer Metamorphosen. — Die Bösartigkeit eines solchen Gewächses beurkundet sich jedoch auf diese und mehrfache andere Art zugleich, wie aus den folgenden Fällen einleuchtet.

Zweiter Fall. Grundfest A., 70 Jahre alt, Gutsbesitzer (chir. Abth. Z. N. 53. Mai 1854). — Zottenkrebs der Harnblase.

Der Patient wurde im Monate Mai 1854 auf der chirurg. Abtheilung des H. Prof. Pitha an Ischurie und Haematurie behandelt. Ein hagerer schlanker, gesund aussehender Mann von 70 Jahren, der in seinem Leben niemals von erheblichen Krankheiten heimgesucht gewesen. Seit anderthalb Jahren bemerkt derselbe ohne bekannte Veranlassung eine blutrothe Färbung seines Urins, die beim Genusse von Wein (der Patient ist in der Weingegend von Melnik zu Hause), immer intensiver, und die Entleerung immer schmerzhafter wurde. Häufig Harnzwang ohne namhafte Entleerung, oder tropfenweiser, schmerzhafter Abgang von blutiger Flüssigkeit; zuweilen plötzliche Verhaltung des anfänglich im vollen Strahle gelassenen Urins, und kurz oder lange darauf, nach Ueberwindung eines Hindernisses, das die Harnröhre „wie ein Stöpsel" verlegte, oder aber bei horizontaler Lage, bald erfolgende Fortsetzung des Strahles; — endlich als Ergebniss der Kathetrisation und der Untersuchung per anum: Hypertrophie der Prostata und der Blasenwandungen, letztere ohne Incrustation und Divertikelbildung, ohne nachweisbares Hinderniss in der Urethra. Mit dem blutigen Harn werden zeitweilig blassgelbliche, in Consistenz verdicktem Schleime ähnliche Flocken entleert. H. Prof. Pitha erklärt, dass diese Harnflocken denen des ersten Falles, wie sie bei Lebzeiten des Patienten abgegangen waren, auffallend ähnlich seien. Die mikroskopische Untersuchung wird zu wiederholten Malen vorgenommen, und liefert jedesmal die unzweideutigsten Resultate. Wir lassen die Bilder der organisirten Bestandtheile des Harnsedimentes der Reihe nach folgen

Am 12. *Mai.* — Der blutig gefärbte Harn wird abgegossen, das Sediment in einem Vogelglas mit Wasser diluirt, die schleimähnlichen Flocken isolirt. Auf den ersten Griff bekommt man bei einer Vergrösserung von 90 (Plössl), theils ein dem vorigen ähnliches dichotomisch verzweigtes (Fig. 3. a), theils ein zierliches blumenkohl-ähnliches Gewächs, das sich als eine Gruppe terminaler Kolben der dendritischen Vegetation erweist. Grösste Breite: 0,025—0,035'''. — *Bei stärkeren Vergrösserungen erkennt man* Fig. 3. *b)* an einem terminalen Triebe den inneren Contour eines Schlauches mit ovalen, in der Längenrichtung aufgelagerten Kernen, und einem mehrfach geschichteten Beleg, der aus grossen, von einer feinpunktirten Masse erfüllten Zellen besteht. *c)* Ein Bruchstück des Stämmchens von *a)*, bestehend aus einem von kleineren einkernigen Zellen gebildeten Röhrchen mit einem centralen, die Achse des Röhrchens darstellenden Strange von fibrillärer Streifung und Auffaserung. *d)* Cylinder, gebildet aus einkernigen, scharf differenzirten Zellen, an den abgerissenen Enden mit deutlichen Umrissen eines Röhrenlumens. *e)* Lose Zellen aus der Belegmasse, polymorph und von excessiver Grösse, mit grossen, oft 2- und mehrfachen Kernen versehen.

Am 13. *Mai. Verschiedene röhrige und membranige Gewebsrudimente*, Fig. 4. *a)* Die Wandung eines structurlosen Rohres mit undeutlicher, die Contouren der Zellenanordnung nachahmender Streifung. *b)* Structurloses Röhrchen von schwarzpunktirten Sedimentmolecülen besäet. *c)* Feingestreiftes, mit ovalen Kernen besetztes Röhrchen, oben aufgerissen, an einer Seite mit einem festhaftenden Reste der Belegmasse versehen, woran die äusseren Zellenschichten durch Fettmetamorphose vom Centrum aus feinpunctirt und verdunkelt, die inneren scharf begränzt und kernhaltig erscheinen. d, e, f, — lose Zellen, wovon: *d)* den Schleimzellen bei Blasenkatarrh, — *e)* den flachen Epitelzellen der Harnblase entsprechen, — *f)* eine grosse Mutterzelle mit Tochterzellen und Kernen darstellt, worin ein Kern zu einem hyalinen Bläschen vergrössert, hydropisch geworden ist.

Am 20. *Mai.* Fig. 5. *Nebst den vorher angeführten Gewebsrudimenten* *a)* Ein ähnliches cylindrisches Röhrchen mit einem centralen Faserstrange, wie in Fig. 3. c. *b)* Dasselbe Rudiment aufgefasert, wobei die Fibrillen den elastischen Fasern gleichen, jedoch feiner sind, und rauher in den Contouren aussehen als letztere. *c)* Strangförmige Fibringerinnsel.

Am 22. *Mai.* Fig. 6. *Eine Blumenkohl-ähnliche Vegetation: a)* Ein Flocken aus dem Harn, ausgebreitet und in natürlicher Grösse gezeichnet; die dunklen Flecke im Inneren bezeichnen den Blutinhalt des Schlauchwerkes. *b)* Ein terminales Bruchstück desselben, Vergr. 180.; die membranige Wandung des blutführenden Röhrchens ist längs-gestreift, hie und da mit oblongen Kernen besetzt, aussen mit lose haftendem Zellenbeleg versehen; am oberen Ende sitzt eine Gruppe von kugelrunden Bläschen (blumenkohlähnliche Endigung des Schlauches) von ähnlicher Structur und gleichfalls blutführend. *c)* Zellen aus der Belegmasse, Vergr. 280., darunter Brutzellen und solche mit hydropisch aufgeblähten Kernen.

Ohne die mikroskopischen Bilder vervielfältigen zu müssen, glauben wir den Medullarkrebs mit dendritischem Typus zur Evidenz eruirt zu haben, und fügen nur noch einige Worte

über das Schicksal des Patienten hinzu. — Eine blande Diät, kalte Sitzbäder, Injectionen von kaltem, später mit einer leichten Alaun-solution versetztem Wasser erleichterten den Zustand des Patienten bedeutend, und gestatteten ihm, der nach seiner Idee zur Steinoperation ins allgemeine Krankenhaus nach Prag gekommen war, binnen wenigen Wochen gebessert nach Hause zu reisen. — Nach einigen Wochen trat wieder Hämaturie ein, und der Patient erlag auf seinem Landsitz den Folgen der hochgradigen Anämie ohne sonstige Zufälle in der Sphäre des Harnapparates.

Dritter Fall. Mühlig Gottfried, 59 Jahre alt, Schneidermeister, starb am 7. Juni 1854 an Urämie (Chir. Abth. Z. N. 132. P. N. 4841). Sectionsbefund: Medullarkrebs der Harnblase, Entzündung der Harnwege und der linken, excentrische Atrophie der rechten Niere. Dickdarmkatarrh. Lobuläre Pneumonie rechts oben. Leichtes Oedem der Unterschenkel.

Patient kam, nachdem er ein ganzes Jahr hindurch beständig an Blutharnen gelitten hatte, in einem ungemein anämischen Zustande ins allgemeine Krankenhaus. Knochenbau und Musculatur kräftig, die allgemeinen Decken ungewöhnlich bleich, die Schleimhäute kaum mehr als blassgraulich gefärbt. Der ohne Hindernisse eingeführte Katheter constatirt die völlige Incrustation der inneren Harnblasenwand; die Harnentleerung geschieht regelmässig unter *Gasentwickelung*, d. h., es gehen mit dem jauchigen, ungemein übelriechenden Brei und den reichlichen Blutgerinnseln zugleich Winde mit einem gurrenden Geräusche durch den Katheter ab. Das Excret setzt ein reichliches, braunerdig-sandiges Sediment ab, das aus allen möglichen Formen von Krystallen und krystallinischen Körpern besteht.

Das Ergebniss der *mikroskopischen Untersuchung* bezüglich des vermuthlichen Aftergebildes ist in diesem Falle ein dürftiges. Als Gewebstheile kommen in den schleimähnlichen Flocken des Harns nur morsche *Fasern* einer *feingestreiften*, oder streifenähnlich feinpunktirten *Substanz* vor, die stellenweise breiter werdend, das Ansehen einer *hyalinen Membran* gewinnt (Fig. 7. a), an den Enden wohl auch das Bild eines aufgerissenen, und in die Fläche ausgebreiteten, *dünnwandigen Röhrchens* bietet (b, c). — Mit dichten, in Zersetzung begriffenen Schleimflocken hat dieses Object eine grosse Aehnlichkeit, und es macht nur der Umstand einen Unterschied geltend, dass die durch fibrilläre Streifung oder Faltung bedingte Zeichnung derselben eine ganz constante Erscheinung bildet, und dass die Membranen und Schleifen trotz der hochgradigen Maceration eine bedeutende Resistenz dem Auswaschen bieten, wodurch man sie von den dichtgehäuften, massenhaften Krystallen zu befreien sucht. — Isolirte *Fasern des elastischen Gewebes* liegen stellenweise frei über den membranigen Schleifen (a). *Grosse eckige und ovale Kerne, und grossblasige Zellen* (d) mit 1—2 beinahe den ganzen Zellenraum ausfüllenden Kernen haften zwar in geringer Zahl an den häutigen Gewebsfetzen, sind aber für uns die sichersten Kennzeichen jenes dicklichen Saftes, den man am medullaren, besonders auch Zottenkrebs als Inhalt und Belegmasse zu finden pflegt. Die Fettumwandlung des Zellinhaltes spricht in Uebereinstimmung mit (e) dem Vorkommen der zahl-

reichen atrophischen Blutzellen, der wolkigen Molecülarmasse, der Fettkörnchen und verschiedener Krystalle für den Zerfall des Aftergebildes.

Die *Leichenöffnung* constatirte die Richtigkeit unserer Vermuthung über den medullaren Charakter des Gewächses. Der *Zustand des Harnapparates an der Leiche* ist folgender: Die rechte Niere klein, dicht, stark erbleicht; die Nierenkelche und Becken, so wie der Harnleiter stark erweitert, mit trüber, dicklicher Flüssigkeit gefüllt. Die linke Niere gross, sehr mürbe, die Cortical-Substanz von zahlreichen kleinen Eiterherden durchsetzt, die Schleimhaut der Nierenbecken dunkelroth injicirt. — In der Harnblase trüber, sehr dicker, flockiger, übelriechender Inhalt, die Blasenhäute, namentlich die Muscularis sehr verdickt, die Schleimhaut missfärbig und mit Harnsedimenten und Exsudatgerinnseln bedeckt, *die Gegend des Blasenhalses in weiter Ausdehnung über das Trigonum nach hinten mit einem weichen, langgestielten, in zarten Zotten zerfallenden, gefässreichen, an der Oberfläche theilweise verjauchten Aftergebilde* besetzt. Die Schleimhaut der Harnröhre gleichfalls missfärbig, stellenweise brandig zerfallen, mit Jauche bedeckt, in der Fossa navicularis von einem unregelmässig ausgezackten, mit mürbem Exsudat und verfilzten Incrustationen bedeckten Geschwüre durchbrochen.

Das faserig membranige Stroma des Pseudoplasmas zeigt mehr oder weniger einen ähnlichen Zerfall, wie di eim Harnsediment beiLebzeiten entdeckten Partikel der dünnhäutigen Schleifen. In diesem Falle ist es mit Zuhilfenahme der Symptomatologie erklärlich, warum bei Lebzeiten kein vollständiges Bild aus den spärlichen Flocken des Harnsedimentes zu gewinnen war. Das Secret, dessen übler Geruch dem Patienten selbst unerträglich wurde, die ungewöhnliche Gasentwickelung beim Harnen, und die Krystallformen des entleerten Breies beurkundeten einen derartigen Zerfall stagnirender und zu Jauche zerweichter Gewebsrudimente, dass an die Erhaltung eines Afterproductes von lockerem zartem Gewebe kaum zu denken ist, da man unter solchen Umständen selbst die Maceration und Morschwerdung der resistentesten physiologischen Gewebe begreiflich finden möchte. In der That waren einige Partien des Aftergebildes an der Leiche bereits breiig geschmolzen, andere sogar vom Mutterboden hinweggespült, der letztere daselbst aufgewühlt, gelockert und von Jauche durchtränkt. Es ist dies wohl der höchste Grad nekrotischer Zerstörung im Harnapparate, der sich mit einer gewissen Fortdauer des Lebens noch verträgt: und dennoch machte es die enorme Destruction dem Mikroskope nicht unmöglich, Gewebsrudimente in dem Jauchebrei zu entdecken, die mittelst Exclusion der physiologisch vorkommenden Gewebstheile, das Vorhandensein eines *membranig-röhrigen Gewächses mit reichlicher Vascularisation und excessiver Zellenwucherung* wenn auch nicht zur Evidenz, doch mit der grössten Wahrscheinlichkeit beweisen.

Uebrigens müssen wir gestehen, dass uns die Beobachtung und gleichzeitige Untersuchung des vorher angeführten Falles nicht wenig darin unterstützte, in diesem, durch weit vorgeschrittene Destruction der Blase getrübten, und der mikroskopischen Behandlung schwierig gewordenen Harnsedimente, die Bilder der losen Partikelchen folgerichtig zu deuten, und in ein Ganzes zusammenzufassen. Wir wollen im

Anhange diese Umstände näher besprechen, den Hergang der mikroskopischen Untersuchung mittheilen, und die dadurch gewonnenen Resultate genauer würdigen.

Vierter Fall: Sinecký Martin, 64 Jahre alt, starb am 26. August 1854 an Urämie (Chir. Abth. Z. N. 68. P. N. 6779). Der Patient kam kurz vor dem Ableben ins allgemeine Krankenhaus.

Die mikroskopische Untersuchung wurde an dem, einige Stunden vor dem Tode mit dem Katheter entleerten Harn vorgenommen. Es war eine schmutzigbraune, trübe, mit Flocken und Blutstriemen untermengte Flüssigkeit, in der sich sandkörnchenähnliche Concremente sedimentirten. Die mikroskopischen Bilder versprachen diesmal nicht viel Ausbeute. Neben reichlichen *Krystallen* des prismatischen Systems (Sargdeckel-, Dach- und ähnliche Formeln), Fig. 8. A., dann *krystallinischen Kugeln* von hellgelber Farbe und Eiterzellen-Grösse, die überall zum Vorschein kamen, gab es nur einen massenhaften Detritus aus *feinkörniger Molecularmasse*, hie und da zerstreute *Körnerhaufen* und kugelige, undeutlich begränzte Massen einer (bei der stärksten Vergrösserung) nur *fein punctirten opaken Substanz* (ähnlich der vorgenannten Matrix im Zungenbeleg, wahrscheinlich im Zerfall begriffene Schleimflocken). *Blutkörper*, theils erhalten, theils atrophirt, freies und körniges *Fett* waren der Flüssigkeit reichlich beigemengt.

In den Flocken zogen nebstdem andere Gebilde unsere Aufmerksamkeit an sich. Es waren da (a) reichliche *Körper mit fein granulirtem Inhalt*, von der Grösse einer Blutzelle und etwas darüber, bald rundlich und oval, bald länglich und spindelförmig, grösstentheils dunkel und scharf contourirt, gegen Reagentien unempfindlich. Hie und da bemerkte man, dass denselben ein Saum einer wolkigen, undeutlich begränzten Substanz anhänge (b). Sowohl diese Körper als die Krystalle erschienen meistens in einem *Flocken lockerer Substanz* eingebettet, die durchscheinend, ohne Formelemente, höchstens mit einer Punktform gezeichneten Streifung versehen war; nur an zwei Präparaten, hergenommen aus einem festeren Flocken, kamen dünne *Schleifen eines feinfibrillären Gewebes* (c) zum Vorschein, die einen gleichmässigen Durchmesser von 0,008‴ — 0,010‴, deutlich begränzte, feine Contouren, und parallel der Längenaxe in beinahe regelmässigen Abständen eingetragene dunklere Streifen, ähnlich den Kernfasern, zeigten. An dem Ende einer dieser Schleifen haften mehrere *Zellen mit grossen Kernen* und deutlichen Kernkörperchen, deren flüssiger Zelleninhalt gleichfalls dunkelpunktirt erscheint. Ihre Kugelform, die scharfen Umrisse, sowie die Grössenverhältnisse (Zelle: 0,006—0,008‴, Kern: 0.003‴—0,004‴) tragen deutlich das Gepräge eines im physiologischen Zustande in der Harnblase nicht zu suchenden Gebildes. Fortgesetztem Nachsuchen gelingt es auf eine *Gruppe* dieser *grosszelligen Gebilde* (d) zu stossen, die durchaus rund, scharf, jedoch zart contourirt, die ähnlichen Verhältnisse des Durchmessers und des Inhalts zeigen, nebstdem aber zu 2—3 *mit einem gemeinschaftlichen Contour* umschlossen werden. — An einem andern, der beschriebenen Schleife ähnlichen Gewebstheile haften ähnliche, mehr zerstreut liegende Zellen mit grossen Kernen, die zuweilen blasig erweitert, auch mit doppelten Kernen versehen erscheinen; neben ihnen kommen zahlreiche, den Zellenkernen vollkommen ähnliche Körper mit scharfem

Umriss und feingranulirtem Inhalt vor, die wie die oben beschriebenen einer Zellenhülle ermangeln.

Auf diesen Befund hin, stellten wir die Vermuthung auf das Vorhandensein eines Neoplasmas auf, und zwar eines *verjauchenden Krebses;* wenn auch das Ergebniss der peniblen Untersuchung ein sehr lückenhaftes war, konnten wir, nach Analogie der früheren Fälle zu schliessen, einen ähnlichen Zustand vermuthen. Die Symptome des Krankheitsverlaufs treten in dem mikroskopischen Bilde unzweideutig hervor: Stagnation des Harns mit krystallisirendem Sediment, reichliche Blutung, Zersetzung des Secretes, Zerfall eines in rascher Entwickelung begriffenen Afterproductes.

Die Untersuchung der nach einigen Stunden neuerdings abgenommenen Flüssigkeit hatte den früheren Befund bezüglich der Zellenelemente nicht blos bestätigt, sondern noch einen neuen Beleg geliefert: *a)* eine Zelle von 0,012''', mit dünnwandiger Hülle, mit einem hyalinen Kernbläschen von 0,008''' und einem wolkig trüben colloiden Fluidum als Zellinhalt. *b)* Eine ähnliche Zelle ohne Kernbildung, einerseits eingerissen und den wolkig-flockigen Inhalt entleerend, andererseits durch Zusammensinken der Wandung eine concentrische Streifung annehmend. *c)* Eine *Blase* von 0,016''' mit *geschichteten*, auf hellem Grunde dunkler gestreiften *Wandungen;* den Inhalt bildet eine gelbliche, trübe, einerseits dunklere Schlagschatten werfende Substanz, die das Ansehen gewinnt, als wäre sie aus ungleich grossen Kugeln dichterer Colloidsubstanz zusammengeballt. Die ganze Blase erscheint rings um die grössere Hälfte in eine *Belegmasse* eingehüllt, deren Formelemente die oben beschriebenen Zellen mit grossen Kernen und Kernkörperchen bilden; diese decken einander zum Theil dachziegelförmig, sind, von oben betrachtet, einer Epitelschicht ähnlich. bei tieferer Einstellung des Rohres geben sie seitlich tiefe Schlagschatten. Die äussersten Lagen lösen sich leicht ab, und zeigen den früher geschilderten, durch feine Punktirung verdunkelten Inhalt.

Obwohl es eine gewagte Sache ist, aus einzelnen Zellen die Diagnose auf irgend ein Neoplasma sicher zu stellen, über dessen gröberen Bau man nach dem Befund kaum eine Vermuthung haben kann, hielten wir die wenigen, jedoch durch ungewöhnliche Grösse ausgezeichneten Zellenformen, auf Grundlage der früheren Fälle für Bruchstücke eines möglicherweise selbst im dendritischen Typus gebauten (Zotten-) Krebses. Die fibrillär gestreiften Schleifen deuteten wir für morsche Bruchstücke von membranigen und röhrigen Gebilden; die Kernzellen für deren Belegmasse, die Zellen mit blasig vergrösserten Kernen für endogene Bildungen, die grosse Blase mit einem dichten Zellenbeleg für ein terminales, von der dendritischen Vegetation abgelöstes Glied. — Die Präparate wurden dem Hrn. Secundärarzt Dr. Matějowský, Hrn.

Dr. Rott aus Tübingen und einigen Herren Aerzten des allgem. Krankenhauses demonstrirt.

Die am andern Tage (27. August 1854) vorgenommene Section hat die Richtigkeit unserer Diagnose neuerdings bestätigt.

Sectionsbefund: Zottenkrebs mit Incrustation und Steinbildung in der Harnblase. Pneumonie beiderseits mit pleuritischen Adhäsionen. Hydrops.

Die Harnblase ist bis etwa zur Grösse einer starken Faust zusammengezogen, und sehr hart anzufühlen. Die Harnröhre vollkommen durchgängig, die Schleimhaut in den tieferen Partien nächst dem Blasenhalse graulich missfärbig; in dem letzteren dickliche Jauche angesammelt. Die Wandungen der Blase am Durchschnitt allenthalben bis auf 5''' verdickt und stark verdichtet. — Zu beiden Seiten des Trigonum Lieutaudii in der Gegend der Mündungen der Harnleiter und darüber hinauf sitzen hasel- bis *wallnussgrosse, über die schmale Basis überhängende Geschwülste* einer dicht verfilzten, an der Oberfläche breiigweichen und schmutzig durchbräunten, beim Entfalten *zottigen Gewebsmasse*, in deren Lücken zahlreiche sandigerdige Concremente eingebettet liegen. An einem ähnlichen, von der hinteren Blasenwand ausgehenden zottigfaserigen sehr morschen Gebilde hängt eine etwa bohnengrosse, rundliche, *steinharte Concretion* in die Höhle der Blase herab, mit dem kugeligen Ende nach abwärts gerichtet, mit dem spitzeren Stiele in die Zotten des Aftergebildes festverfilzt. Ein haselnussgrosser rundlicher *Harnstein* liegt frei in der Blase zwischen den knapp anliegenden Wandungen. Die Schleimhaut daselbst geschwürig aufgerissen, das submucöse Stratum stark aufgelockert und zerweicht. — Bei näherer Betrachtung der Schleimhaut findet man in der Tiefe der bezeichneten Vegetationen *perlgraue Erhabenheiten* (ähnlich der serösen Schwellung der Darmschleimhaut), von deren Oberfläche sich zarte, als kleine Punkte eben wahrnehmbare Knötchen erheben. — Die Durchschnittsfläche des Neoplasmas entleert eine reichliche, rahm-ähnlich dickliche, mit Blut stark untermengte Flüssigkeit, nach deren Entfernung mittelst eines Wasserstrahls eine reichliche radiäre Vascularisation des Gewebes zum Vorschein kömmt. Beide *Harnleiter* sind stark ausgedehnt, dünnwandig, mit wässerigem Harn bis in die Nierenkelche und Becken erfüllt; die Substanz der Nieren hart und fest, blassbraun, in der Corticalis einige hanfkorngrosse colloide Cystchen. — Die *Samenbläschen* in ihren Wandungen stark verdickt, der umhüllende Zellstoff verdichtet, das Gewebe der *Prostata* blassblutig durchtränkt (Leichenmaceration).

Die *mikroskopische Untersuchung* erwies die *dendritische Vegetation mit reichlicher Vascularisation* und massenhafter *endo- und exogener Zellenbildung*, worüber wir uns ausführlich zu verbreitern für entbehrlich halten, da der Fall mit den vorhergehenden (1. und 2.) beinahe gleichlautend ist, und das Wichtigste aus dem bereits Angeführten erhellt. Nur insofern sei es uns erlaubt, das Neoplasma durchzumustern, um nachzuweisen,

inwiefern die Deutung der, unserer Diagnose zu Grunde liegenden Bruchstücke desselben eine richtige war. — Die morschen Zotten in der Harnblase, welche begreiflicherweise vermöge ihres losen Zusammenhanges mit dem Mutterboden, beim Katheterismus am leichtesten abgingen, entsprachen vollkommen den membran-ähnlichen Schleifen (Fig. 8. B.) mit den Längsstreifen und den reichlichen dicht aufsitzenden Krystallen. — Die grossen kernhaltigen Zellen (Fig. 8. B. d), bildeten in der That die luxurirende Belegmasse des röhrig ausgewachsenen Stroma, und dass sie nicht in grösserer Menge im Harnsedimente erschienen, ist aus dem Umstande erklärlich, dass sie nur in der Tiefe der Vegetation wohlerhalten, je weiter gegen die Oberfläche, je rascher zerfallend, und daher beim Wegspülen nur unter günstigen Verhältnissen unversehrt zu Tage gefördert werden konnten. Polymorphe Zellen findet man vorwaltend in dichten Partien des mit Krebssaft reichlich versehenen Gewächses. — Die grossblasigen Zellen (Fig 9) erwiesen sich endlich als endogene terminale Glieder der kolbigen Auswüchse, wie sie am Zottenkrebs allenthalben vorzukommen pflegen. In Bezug auf diese letztgenannten Gewebstheile des Zottenkrebses müssen wir erwähnen, dass die schönsten Formen an den als perlgraue Erhabenheiten der Schleimhaut beschriebenen Wulstungen der Blasenwand zu finden, und die Entwickelung der Vegetation daran von Glied zu Glied zu verfolgen ist. — An dem mit einer Scheere abgelössten Schleimhautwulst (Fig. 10) sieht man (bei einer Vergrösserung von 90, Plössl) rundliche gestielte Bläschen, theils isolirt, theils zu 2—3 gruppirt, mit scharfem einfachem Umriss und einem scharf contourirten kugeligen Centralkörper sich erheben; daneben bemerkt man kürzere und längere Kolben, die längergestielten tragen seitliche Bläschen, die zu einer cactusähnlichen Verzweigung führen, an der man wieder hie und da das seitlich aufsitzende Bläschen findet. — In den dickeren Kolben verlaufen bluterfüllte Gefässe mit einfachen oder etwas gekrümmten Endschlingen; in der Tiefe zeigt das Präparat eine dunkle Längsstreifung von den dicht neben einander verlaufenden Gefässen. Das ganze Bild erinnert an die Zotten der Darmschleimhaut, wenn man sie im injicirten Zustand bei schwacher Vergrösserung betrachtet.

Fünfter Fall. In einem (neuerdings von der II. Int. Abtheilung des H. Prof. Halla mitgetheilten) Harn wurden in ähnlicher Weise die Producte einer excessiven Zellenwucherung

nachgewiesen, wobei besonders endogene Bildungen von Zellen und Kernen, sowie reichliche Gruppen polymorpher Elemente die Diagnose eines Medullarkrebses begründeten, ohne dass man Bruchstücke eines Stromas entdeckt hätte. (Fig. 11.) A. die vorherrschenden Zellenformen: *a)* einkernige kugelige Zellen, — *b)* verschiedene Formen mehrkerniger und in Fettumwandlung begriffener Zellen; — *c)* und *d)* colossale Schachtelzellen; — B. ein mikroskopisches Bild der vorigen Elemente mit den prismatischen Krystallen. Als wir hörten, der Harn sei von einem Weibe, wünschten wir eine Sicherstellung vor der Vermengung des Excretes mit anderweitigen. allenfalls aus der Vagina oder aus dem Uterus abgegangenen Gewebstheilen. Der Harn wurde mit dem Katheter abgenommen und enthielt dieselben Formelemente, wie vorher. Das Carcinom wurde sonach mikroskopisch entdeckt, die Harnblase als Sitz desselben bezeichnet, später jedoch das Pseudoplasma auch im Uterus durch manuelle Untersuchung constatirt. — Der kurz darauf erfolgte Tod machte die Autopsie möglich. Der Sectionsbefund war (J. A. 63 J. alt N. P. 8376, am 10. November 1854): *Medullarkrebs des Uterus, des Fornix vaginae uud der Harnblase;* — beiderseitiges Lungenödem mit obsoleten pleuritischen Adhäsionen, Darmcatarrh, Anämie, Marasmus.

Wenn man sich längere Zeit in der histologischen Untersuchung nekrotischer Gewebe übt, und zugleich die durch ähnliche Bedingungen wie im Organismus, z. B. durch Maceration veränderten Organtheile studirt, so gelangt man bald zu einer Fertigkeit in der Darstellung und zu einer Sicherheit in der Deutung der verschiedenen im Zerfall begriffenen Vorkommnisse. Der Ungeübte sieht darin nichts mehr als einen stinkend-faulen Brei, und aus diesem etwas Lebend-dagewesenes restauriren zu wollen, dünkt ihm ein blosses Spiel der Imagination. Indessen kann sich Jedermann überzeugen, dass das thierische Gewebe — d. i. neben den Knochen die meisten Weichtheile — eine wunderbare Resistenz zeigen, und wenn Czermak bei seinen histologischen Untersuchungen der ägyptischen Mumien über die tausend und tausendjährige Erhaltung der Gewebselemente in Staunen gerieth, so wird man von der Widerstandsfähigkeit animaler Gewebe in ätzenden Jauchen nicht weniger überrascht sein.

Das Mikroskop reicht nicht blos zur Entdeckung der organischen und der *organisirten Bestandtheile eines Harnsedimentes* hin, sondern es ist gegenwärtig der alleinige competente Schieds-

richter in zweifelhaften Fällen der Diagnose. Als Vorkommnisse werden bisher allgemein angegeben: *Blut, Schleim und Eiter, Epitelien, Spermatozoiden und endlich die sogenannten Harncylinder* (Exsudat- und Fibringerinnsel). Den Unterschied der einzelnen Gewebstheilchen vermag die Chemie nur höchst dürftig oder auch gar nicht anzugeben, indem sie hiebei nur die Gegenwart von Albumin-hältigen Substanzen nachzuweisen im Stande ist. Hingegen machen es die *Formeigenthümlichkeiten* der einzelnen organischen Elemente dem Mikroskope möglich, oft auf den ersten Griff die Diagnose festzustellen. Dennoch ist der Nachweis der angeführten Gewebstheile oft von untergeordnetem Werthe.

Schleim- und *Epitelialzellen* sind regelmässige Vorkommnisse beim physiologischen Hergang der Harnsecretion. — *Eiterkörper* sind in spärlichen Massen von Schleimzellen nicht zu unterscheiden, und es wäre ihr Nachweis vielleicht erst dann von Belang, wenn sie ein schon mit blossem Auge wahrnehmbares, gewöhnlich blass- oder grünlichgelbes Sediment im Harn absetzen. Sie haben unter Umständen, namentlich bei geringer Alkalescenz, und im Kühlen, eine bedeutende Widerstandsfähigkeit gegen die Fäulniss, gegen Hitze und gegen Reagentien. In einem eiterhältigen Harn, den wir mit Salpetersäure leicht versetzt hatten, konnten wir die hellgelb gefärbten Eiterkörperchen noch am neunten Tage genau erkennen. Bei acuten Entzündungen der Harnblase will man beobachtet haben, dass das *croupöse und diphtheritische Product* sich abgestossen habe und Heilung erfolgt sei; ob man dieses in dem abgegangenen Harn gefunden und als solches erkannt habe, ist nicht angegeben. Uns fehlt darüber die Erfahrung. — *Blutkugeln* werden wohl in den kleinsten Quantitäten, wo sie keine Röthung des Harns vermuthen liessen, leicht entdeckt; dann haben sie aber für sich allein eben keine erhebliche Bedeutung, und sind wieder anderentheils — wenn in grösseren Massen — bei den verschiedensten Erkrankungen der Harnorgane von den Nieren angefangen bis zum Orific. urethrae ext., ja selbst bei Rückenmarksleiden (Paralyse), oft in grosser Quantität, vorhanden. Ihre Entdeckung in einem durch forcirte Einführung des Katheters entleerten Harn, oder bei Frauen in der Menstruationsperiode, ist ganz ohne Belang. — Nur die schrumpfenden, zu sternförmigen Körperchen atrophirten Blutzellen verdienen mehr Beachtung, insofern als sie im frisch gelassenen Harn

eine längere, durch irgend ein Hinderniss der Entleerung bedingte Stagnation vermuthen lassen.

Epitelialzellen, *Spermatozoiden* und kleine unregelmässig rundliche, *gelbe Körperchen* (nach ihrer Indifferenz zu schliessen, *Colloidkörnchen*, vielleicht normgemäss aus den Zellen und Zellenkernen der Littre'schen Drüsen, und aus dem Secret der Prostata) scheinen uns ebenso unwichtige, theils normal, theils zufällig, jedenfalls ohne diagnostishe Bedeutung vorkommende Bestandtheile des Harnsedimentes auszumachen. Die Einführung des Katheters mag bezüglich der Spermatozoiden in vielen Fällen bei der am Lebenden vorgenommenen Entleerung des Harns dieselbe Rolle spielen, wie das Ausschöpfen desselben mittelst eines Löffels aus der an der Leiche eröffneten Harnblase. Man drückt nämlich die Ductus ejaculatorii, die Prostata, vielleicht auch mit dem gleichzeitig per anum eingeführten Finger die Samenblasen selbst, — und dann wäre es wahrlich ein Wunder, wenn die Flüssigkeiten aus diesen Partien *nicht* entweichen, und im Harn *nicht* erscheinen sollten. — Die *Colloidkörper* erscheinen im Harn zuweilen in grösseren Massen, sie sind aber an keine Stelle des Harnapparates ausschliesslich gebunden, da bekanntlich die colloide Umwandlung des Zellinhalts besonders an den Epitelien des gesammten Tractus, von dem Malpighischen Glomerulus in der Niere bis zur Littre'schen Drüse in der Urethra beobachtet worden ist.

Was die *cylindrischen und schlauchförmigen Körper* anbelangt, so verdienen sie vorzugsweise unsere Beachtung als die ungewöhnlichsten Erscheinungen in krankhaften Affectionen des Harnsystems. Sie sind seit lange Gegenstand der Beobachtung gewesen, und von Frerichs (die Brightische Nierenkrankheit 1851) und Lehmann genau beschrieben worden. Man unterscheidet da folgende Formen:

a) Cylinder aus Epitelialzellen der Bellinischen Röhrchen bei entzündlichen Zuständen der Nieren; die rundlichen, kernhaltigen Zellen sind mit feinkörniger Molecülarmasse gefüllt; keulenförmige und bipolare, gleichfalls kernhaltige Epitelialzellen aus den Ureteren und Nierenkelchen kommen mit denselben in losem Zustande vor.

b) Exsudat-Gerinnsel, entweder amorph oder cylindrisch und röhrig geformt, gleichsam Abgüsse der Bellinischen Röhrchen darstellend; sie bilden theils unregelmässig begränzte, mehr oder weniger durchscheinende, die makroskopischen Ge-

rinnungen nachahmende, undeutlich-zackig contourirte Fetzen, theils aber zusammenhängende Streifen von gleichmässigem Caliber und zuweilen kolbig abgerundetem Ende, schliessen häufig Blut- und Eiterzellen ein, und erscheinen nebstdem durch dichte feine Punktirung mehr oder weniger verdunkelt. Lose Blut- und Eiterzellen, so wie doppeltgeschwänzte Epitelien begleiten ihr Vorkommen in der Brightischen Nierenkrankheit.

Hierher gehören wohl auch die sogenannten schlauchförmigen Körper, d. h. homogene, *hyaline Cylinder*, häufig zusammengefallen und gefaltet, oder um ihre Axe gewunden, nur bei starker Vergrösserung erkennbar, nach Lehmann (II. 391) in der chron. Form der Bright'schen Krankheit vereinzelt zu finden. Der von Neubauer T. 1. Fig. 5. gezeichnete Fall von einem mit acuter Milztuberculose Behafteten, den man schon in Funke's Atlas der physiologischen Chemie (Leipzig 1853. S. 34. Taf. 14. Fig. 2) wörtlich findet, in welchem die Cylinder durch Anfüllung mit kleinen Körnchen von harnsaurem Natron (?) stellenweise deutlicher hervortreten, liesse sich ebensowohl als ein unter *b)* beschriebenes helleres Schleim- oder Fibrin-Gerinnsel deuten; denn dass diese Cylinder *hohl* seien (Neubauer S. 64), ersieht man weder aus der Beschreibung, noch aus der Abbildung, und es gibt auch keinen Umstand, auf den hohle Gebilde zu beziehen, und wie sie zu erklären wären. Denn Bellinische Röhrchen der Nieren, d. i. ihre Membrana propria, die das einzige physiologisch praeexistirende Schlauchgebilde abgeben, das man im Harn vermuthen könnte, gehen nach unserer Ueberzeugung niemals ab, und könnten höchstens bei Nekrose der Nierensubstanz in Folge chronischer Nephritis, niemals aber bei der Brightischen Nierenkrankheit im Harn gefunden werden. — Dass aber Schleim- und Fibrin-Gerinnsel die Cylinderform häufig annehmen, hat nicht bloss Frerichs in der Bright'schen Krankheit (Fig. 32—41) nachgewiesen, sondern man kann sich auch an frischen Exsudaten der *serösen* Säcke sehr leicht davon überzeugen, wie deren Cylinder die Röhrchenform oft täuschend nachahmen, also selbst da, wo sie nicht durch präformirte physiologische Röhrengebilde dazu gezwungen werden, wie dies in der Niere der Fall zu sein scheint.

Wenn schon diese Möglichkeit einer Verwechslung der Exsudat-Gerinnsel mit organisirten Schläuchen zur grössten Genauigkeit bei der mikroskopischen Untersuchung auffordert, so

wird diese noch dringender geboten durch den Umstand, dass Harnsedimente bei hochgradiger Destruction der Blasenschleimhaut kleine Fetzen aus dem *submucösen Zellstoff* enthalten können, deren Erkenntniss mit noch grösseren Schwierigkeiten verbunden ist, wie aus folgenden zwei Fällen erhellet.

Sechster Fall. Burian Josef, 60 Jahre alter Taglöhner, starb am 2. August 1854 an *Scirrhus vesicae urinariae.* (II. Int. Abthg. Z. N. 41. P. N. 4693).

Sectionsbefund: Hypertrophie, Incrustation und Divertikelbildung der Harnblase in Folge von chronischem Katarrh. Rigidität des gesammten Arteriensystems. Allgemeiner Marasmus und Anämie; obsolete pleuritische Adhäsionen beiderseits, mit frischer Pleuritis rechts. Linksseitige Hydrocele. Trichocephalus und Ascaris im Blinddarm.

Wir entnehmen dem Protokoll die näheren Angaben über den Zustand der Harnblase: Dieselbe ist in den Wandungen stark verdickt, etwas zusammengezogen, mit dem kuglig vorspringenden, hartanzufühlenden Vertex nach rechts geneigt. Die Schleimhaut an zahlreichen Stellen dunkel geröthet, mit kalkbreiähnlichen Sedimenten dicht bedeckt, im Fundus mit rundlichen Divertikeln versehen, welche kleinere und grössere Concretionen umschliessen, im corpus trigonum und im Blasenhalse aufgerissen und mit Incrustaten fest verfilzt. Der Inhalt der Harnblase, sowie der stark erweiterten Ureteren eine trübe, blutig gefärbte Flüssigkeit. Die in der Nähe der aufgerissenen Stelle der Schleimhaut am Blasenhalse stark vorspringenden, theils durch seröse Schwellung, theils durch Injection gelockerten Partien boten dem blossen Auge eine täuschende Aehnlichkeit mit einem Neoplasma, und die nähere Untersuchung war um so mehr nothwendig, als die aufgefaserte Oberfläche des benachbarten Substanzverlustes für den Rest eines weggespülten Theiles der Neubildung angesehen werden konnte. Man findet darin neben morschen, kaum mehr eine sichere Deutung zulassenden Partikelchen organischer und unorganischer Substanzen aufgefasertes Bindegewebe in unregelmässig eingerissenen Plaques, stellenweise in membran-ähnlichen Ausbreitungen, und hie und da Bruchstücke elastischer Fasern in einer unvollständigen Netzform, etwa wie oben in Fig. 5. b. — Alle diese Gewebstheile sowie die organischen Muskelfasern sind von Krystallen, Sedimentmolecülen und einer trüben formlosen Flüssigkeit mit Fettkörnchen und atrophirten (sternförmig-kerbigen) Blutkörperchen dicht durchsetzt. Ueberall jedoch vermisst man die gleich- und regelmässigen Contouren der Bindegewebs-Membranen, die Röhrchenform und deren Begleiter bei Neoplasmen: die Belegmasse von kugelig-geformten oder polymorphen, grosskernigen Zellen.

Eine ähnliche Beobachtung machten wir kurz darauf in folgendem *siebenten Falle*:

Krögler Anna, 51 J. alt, starb am 31. August 1854 an *Tuberculose* (II. Int. Ab. Z. N. 32. P. N. 5893).

Sectionsbefund. Bronchialblennorrhöe und obsolete Pleuritis. Sphacelöse Caverne im rechten oberen Lungenlappen. Dysenterie des Dickdarms. Geschwüre und Blennorrhöe der Vagina. Chronischer Katarrh mit Hypertrophie und Incrustation der Harnblase.

Der mikroskopische Befund der in ähnlicher Weise wie im vorigen Falle aufgewühlten Schleimhaut ist zum Theil in Fig. 12. dargestellt. Die Schleifen des Bindegewebssubstrates (a) sind unregelmässig zackig gefranst und aufgerissen, die Fläche fibrillär gestreift und mit dichtgedrängten dunklen spindelförmigen Körpern besetzt, hie und da mit elastischen Fasern verfilzt, die Ränder des Präparates mit krystallinischen Kugeln (b) behaftet, welche bei einem Durchmesser von 0,005‴—0,009‴ gelbbraun, mattdurchscheinend und rauhen Colloidkugeln ähnlich sind, und neben anderen kugeligen Massen (c) von amorpher erdiger Substanz und einem Durchmesser von 0,050‴—0,090‴, der morschen Faser allenthalben einen weisslichen kalkmörtelähnlichen Beschlag geben. — Neben dieser Incrustation der organischen Substanz findet man massenhafte grosse Krystalle (d), theils glatt und scharfkantig, theils rauh und mit schadhaften Flächen (bei d*), endlich atrophische Blutzellen, Detritus, Fett, Monaden und Vibrionen.

Die pathologisch neugebildete *Zelle* bot an den unserer Untersuchung zu Grunde liegenden Aftermassen keine charakteristischen Formeigenthümlichkeiten, wodurch man prima vista ihre bösartige Natur erkannt hätte. Die Polymorphie kommt der Krebszelle ebensowohl wie der physiologischen Epitelial-Zelle zu, ja es ist sogar die ungewöhnliche Grösse und die Mehrzahl von Kernen in einer und derselben Zelle, sowie das grosse hellglänzende Kernkörperchen nichts eigenthümliches, der neogenen Zelle ausschliesslich zukommendes, wodurch sie unter allen Umständen herauszufinden wäre. Man wird bei einem frischen Blasenkatarrh, wo das Epitel reichlicher abgestosen wird, gewiss auch einzelne Zellen finden, die man sonst für Krebszellen anzusehen geneigt wäre, da ihre Polymorphie, Grösse, Mehrzahl von Kernen und alle ungewöhnlichen Merkmale dafür sprechen, und die Täuschung kann dadurch noch leichter zu Stande kommen, da man es mit einer exessiv grossen Menge von Zellen zu thun hat, worunter jene grossen Formen eben auch zahlreich vorkommen werden.

Dagegen kommen uns zur Diagnose der neogenen Zelle folgende Umstände zu Hilfe: Die *endogene* Bildung, welche die vorherrschende ist, — die *Mehrzahl* und die excessive Grösse

der Kerne, — die Kugelform bei einer Grösse, welche selbst durch Imbibition aufgeblähte Schleimzellen (0,004—0,005‴) übertrifft; — zuweilen ihre *Fettmetamorphose*, zuweilen wieder ihre Umwandlung theils zu kugeligen *hyalinen Blasen*, theils zu gelben, opaken *Colloidkugeln*, — der Zusammenhang und die Gruppirung vieler Zellen zu kugelig zusammengeballten Haufen, — dann eine gewisse Fülle, Prallheit und Wölbung der Zelle, die entsprechend dem Charakter des Neoplasma, die rapide Wucherung beurkundet, und dadurch oft einen leichter am Präparat zu ersehenden als zu beschreibenden Typus erhält. Dieser spricht sich in einer grossen Summe von Zellen häufig darin aus, dass die Krebszelle durch den dunkler punktirten moleculären Inhalt etwas opaker erscheint als das normale Pflasterepitel, das gleich einem Hornplättchen flach, nur mattpunktirt und daher mehr durchsichtig ist.

Ebensoviel, wo nicht noch mehr Vorsicht, erheischt die Untersuchung des *Stroma*, dessen Bruchstücke als röhrige oder membranige Schleifen aufzufinden wären. Die bedeutende Länge, Breite und Resistenz des entdeckten Rudimentes, die Form im gesammten Umriss und die fibrilläre Streifung sind die wichtigsten Unterscheidungsmerkmale dieses Gewebes von anderen röhrenähnlichen Gebilden. Lose Fragmente der *elastischen Faser* dürften zuweilen bei Substanzverlusten der Schleimhaut aus dem submucösen Zellstoff mit erscheinen, allein man müsste sich dessen versichern, dass sie mit dem in Frage stehenden Gewebsfragment nicht in organischem Zusammenhange stehen. Indessen könnte es vorkommen, dass Partikel aus der Tiefe des Stroma durch vollendete Bindegewebselemente verstärkt, auch im organischen Zusammenhange mit den sonstigen Gewebstheilen im Harnsedimente zu finden wären. — Nicht selten ahmen die parallel laufenden äusseren Contouren röhriger Gebilde die Krümmung der elastischen Faser nach, und man kann sich an frisch präparirten vom Epitel befreiten Harnröhrchen (d. i. der membrana propria der tub. Bellini) überzeugen, dass man die doppelt gezogenen Umrisse des Cylinders viel früher erblickt, als die Röhrchenmembran selbst. — Je früher man an die Untersuchung geht, desto deutlichere, sichere Resultate darf man erwarten; denn da die Blasenschleimhaut im normalen Zustand keine Zotten wie die Darmschleimhaut enthält, wird man in einem ähnlichen wie in unserem 3. Falle vielleicht auf den ersten Griff die Diagnose unumstösslich feststellen können. Ist

hingegen das Gewebe bereits morsch, so werden alle anderen Symptome zusammengenommen mehr Aufschluss geben, als das Mikroskop allein. Es ist sogar denkbar, dass sich das Aftergebilde völlig abstösst, so, dass nur der aufgerissene Mutterboden übrig bleibt, — und es wäre zu wünschen, dass dies lieber zum Vortheil eines Menschenlebens als einer mikroskopischen Diagnose Statt fände, — allein nach unseren bisherigen Erfahrungen erfolgt es im natürlichen Verlaufe leider erst zu einer Zeit, wo der Organismus an Anämie zu Grunde geht.

Es könnte die Frage gestellt werden, ob das Medullar-Carcinom anderer Eingeweide, namentlich der Zottenkrebs des Magens, am Krankenbette nicht auch vermittels des Mikroskops entdeckt werden könnte. Im Vorhinein ist es nicht undenkbar, allein überall werden sich der Untersuchung schwierigere Hindernisse in den Weg stellen, als bei der Harnuntersuchung. — Was zunächst den Zottenkrebs des Magens anbelangt — ein Gebilde, das wir im Verlaufe des Jahres 1854 viermal zu sehen Gelegenheit fanden — so liegt die grösste Schwierigkeit der Untersuchung schon darin, dass abgestossene Theile der Neubildung in der Regel ihren Weg durch den langen Darmcanal finden werden, und daher im halbverdauten Zustand unter den Excrementen kaum, oder auch gar nicht mehr zu erkennen wären. Den kürzeren Weg durch den Oesophagus dürften solche Gewebsmassen selten nehmen, wenigstens ist dies in den von uns verzeichneten Fällen niemals der Fall gewesen.

In einem derselben hatte der Patient nicht die geringsten Magenbeschwerden, litt niemals an Erbrechen und starb, ohne dass man ein Magenleiden je vermuthet hätte, an tuberculöser Lungenphthise (Brustabtheilung December 1853). Sein Appetit war immer sehr stark — er hätte immer mehr, als die ganze Portion verzehrt; — und so enthielt denn der Magen bei der Section auch ein ganzes unverdautes Mittagmal. Die Zottengeschwulst (Demonstr. in der Plenarversammlung) sass in diesem Falle an der Cardia von Seite des Fundus gegen die kleine Curvatur wuchernd, und die Mündung des Oesophagus einigermassen locker verlegend.

In einem andern Falle war es ein kräftiger Mann, der lange Zeit hindurch an den verschiedensten Magenbeschwerden in der Behandlung des H. Prof. P i t h a stand. Er ging später aufs Land und lebte unter den sonderbarsten Wechselfällen

zwischen gesundem Aussehen und krankhafter Abmagerung drei volle Jahre. Sein Befinden hielt durch die ganze Zeit gleichen Schritt mit den Magenzuständen, deren abnorme Symptome in zeitweiliger Appetitlosigkeit, Magenschmerzen und Krämpfen bestanden. In der letzten Zeit hatte er wieder guten Appetit, und ass regelmässig bis zum Tode. Die Section erwies diesmal eine ausgiebige materielle Grundlage des Uebels: es war dies eine bis ans Unglaubliche gränzende Zerstörung der Magenschleimhaut durch theils abgestossene, theils zottig wuchernde Vegetationen, deren erhaltene Reste in der Pylorushälfte an der grossen Curvatur sassen. Das Präparat wurde von dem practischen Landarzt dem H. Prof. Pitha zugeschickt und der pathol.-anatomischen Sammlung geschenkt.

Ein dritter Fall wurde als Scirrhus ventriculi (Pospíšil Johann, 59 J. alt. II. Int. Abthg., P. N. 7126; starb am 1. Septbr. 1854) durch kurze Zeit im allg. Krankenhause beobachtet. Appetitlosigkeit, Magendrücken, Verdauungsschwäche waren die betreffenden Erscheinungen; an Erbrechen hatte der Patient nie gelitten. Der Magen war an der kleinen Curvatur mit dem linken Leberlappen und dem Diaphragma innig verwachsen, an der Schleimhautfläche mit theils knotigen, theils zottigen Neoplasmen bedeckt, so dass kaum $1/3$ der gesammten Fläche (am Fundus) frei und glatt erschien. Die Cardia war auch diesmal durch einen wallnussgrossen Pfropf einer zottigen Neubildung verlegt, und zugleich durch die äussere An wachsung derart geknickt, dass man vom Oesophagus aus nur durch eine schmale S-förmig gekrümmte, nach links gerichtete Lichtung in die Magenhöhle vordringen konnte. Nebst unverdautem Speisebrei lag in der hinteren Magenhöhlenwand ein beinahe hühnerei-grosser Klumpen einer morschen, einerseits zottig flottirenden, andererseits compact verfilzten Gewebsmasse, ein Rudiment des Gewächses, das sich von der vorderen Wand der Pylorushälfte des Magens losgelöst hatte.

Das Mikroskop lieferte uns in diesem letzten Falle auffallend übereinstimmende Resultate mit denen bei der Untersuchung des Harnblasenkrebses. — Es unterliegt übrigens keinem Zweifel, dass die Contenta des Darmschlauches, namentlich erbrochener und durch den Stuhlgang entleerter Inhalt noch lange nicht mit gehöriger Sorgfalt untersucht, und die mikroskopische Ausbeute für Diagnostik noch nicht verwerthet ist. Dasselbe gilt von anderen Organhöhlen ebenfalls, und nebenbei finde hier die Erwähnung einen Platz, dass unter den Sputis

auch Echinococci gefunden werden, die sich aus — selbstverständlich nicht tuberculösen — Cavernen in die ersten Luftwege und nach aussen den Weg bahnen.

Um auf den Harnapparat zurückzukommen, wollen wir eine Beobachtung einschieben, betreffend ein dendritisch-papilläres Pseudoplasma von nicht carcinomatöser Natur.

Achter Fall. Papilläres Pseudoplasma aus der Harnröhre eines Mädchens. Total excidirt von H. Prof. Seyfert. Am 14. Juli 1855.

Dieses schön organisirte in seiner Grösse und Form höchst seltene Gewächs ist merkwürdig durch seinen Standort an der Urethralschleimhaut, wo demselben keine physiologischen Papillen zu Grunde liegen. Es bestand durch 14 Jahre ohne zu bluten und ohne andere dringende Zufälle als die eines Hindernisses beim Uriniren zu verursachen. — Fig. 13. A. versinnlicht das exstirpirte Gewächs in seiner natürlichen Grösse, woran (a) die Schnittfläche mit weitklaffenden Gefässröhren präsentirt, — (b, b) die dendritisch papilläre Verzweigung der röhrigen Auswüchse darstellt. Fig. B. versinnlicht den Bau der terminalen Papillen bei 400maliger Vergrösserung. Man findet in jeder Papille ein zierliches Gefässnetz innerhalb einer weichen von zarten Sepimenten durchzogenen Substanz, die nach aussen von grossen polygonalen, vorwaltend einkernigen, mehrfach geschichteten Pflasterzellen bekleidet erscheint; die letzteren bilden an den Contouren die Form eines breiten Saumes, der anscheinend aus spindelförmigen Kernzellen in mehrfachen Lagen besteht.

Das locale Verhalten, der Zustand einer hochgradigen dichten Organisation, die sich langsam heranbildet, aller Zellenwucherung im Sinne der bösartigen (medullaren) Geschwülste ermangelt, namentlich weder eine endo- noch eine exogene Krebsmasse bietet, steht mit dem ruhigen und langjährigen Bestande ebenso sehr in Einklang, als mit den vorher angeführten Fällen im grellen Contraste.

Endlich seien zwei später zur Untersuchung gekommene Fälle hier angeführt, da die dabei gewonnenen Bilder ein neues Interesse bieten.

Neunter Fall. Harnblasen-Krebs bei einer Frau.

E. A., 43 J. alt, kräftiger Constitution, Mutter mehrerer Kinder. Seit dritthalb Jahren von verschiedenen Aerzten behandelt, die ihr Leiden entweder für Hämorrhoiden, oder für einen weichen Stein erklären zu müssen glaubten. Die Behandlung mit Hanfmilch, Leinsamenabsud und öhligschleimigem Getränk blieb ohne Erfolg, der Gebrauch von Mineralwässern brachte keine Aenderung, warme Bäder und Umschläge auf die Kreuzgegend immer nur Verschlimmerung. Die wichtigsten Symptome waren bisher, nach der Aussage der Patientin, neben unaufhörlichen brennenden *Kreuzschmerzen*, und zeitweilig schiessenden Stichen in den unteren Extremitäten, das *Bluthamen* und der Abgang einer breiigen, nach „Phosphor" stark übelriechenden Flüssigkeit mit *Schleimflocken und Klumpen*, die zuweilen „wie Blutegel dick," zusammenhängend und weich erschienen. Träger

Stuhlgang — mit dem Ausdruck „wie verkeilt“ bezeichnet; — Zunahme der Blässe, Schwäche, Empfindlichkeit und Schmerzäusserung.

Die manuelle Untersuchung ergibt folgenden Befund. Der eingeführte Katheter erweist nicht das Vorhandensein eines Steines; im Mastdarm Coprostase, keine Hämorrhoiden; das Vaginalrohr unter dem Fornix beengt, u. z. durch einen rundlichen Tumor, der die vordere Vaginalwand vorwölbt und dem untersuchenden Finger ein Hinderniss entgegensetzt, wie wenn der Uterus horizontal gelagert, mit dem Muttermunde gegen das Rectum, mit dem Fundus nach vorn unten gerichtet wäre. Der abnorme Körper erweist sich als eine festweiche Geschwulst von mehr als halber Hühnerei-Grösse, eingelagert in die hintere Wand der Harnblase nächst dem Blasenhalse, die vordere Vaginalwand vorwölbend, ohne deren Continuität zu unterbrechen.

Der trübe mit Blut, reichlichem Sediment und schleimähnlichen Flocken untermengte Harn wird durch etliche Tage mikroskopisch untersucht.

Die *Flocken* (Fig. 14. a und b in natürl. Grösse, c bei sechsmaliger Loupenvergrösserung) sind theils *dünnfädig* (a), theils in Form gerissener Brocken, theils in *zusammenhängenden* und Stücken bis zur Dicke einer Kleinfinger-Phalanx vorhanden, grösstentheils weich, locker, blassgelb, den Gerinnungen sogenannter plastischer Exsudate ähnlich; einige erscheinen faserig-brüchig und zerreisslich, blutig gestriemt und durchtränkt; endlich findet man zarte vielfach gefaltete und gerissene Membranchen mit *zapfenförmigen blutstrotzenden Bläschen* (c), die theils isolirt an dem membranigen Gerüst hängen, theils in Gruppen zu einer Art Traube vereinigt beisammen stehen.

1. Die *fädigen und membranigen Partikelchen* erweisen sich unter dem Mikroskop als *einfache Häutchen*, theils in Form von Schleifen (mit einem Breitendurchmesser von $^1/_{10}$—$^1/_{20}$ Lin., dann von 0,020—0,060‴), theils als Bruchstücke grösserer Ausbreitungen. Das Bild derselben hat fortlaufende Contouren, deren Schärfe nur hie und da durch feine Punktirung (in Folge von Maceration) rauh und wie angenagt erscheint; die Fläche ist von matten, feinpunctirten Streifen in der Längenrichtung gezeichnet, und mit rundlichen, in ziemlich regelmässigen Abständen zerstreuten Körpern versehen, die im Ganzen mit einem *Kern- oder Zellenbeleg* röhrig-membraniger Gebilde (Fig. 15. A und B) eine unverkennbare Aehnlichkeit haben, obwohl eine Detailuntersuchung an ihrer Natur nichts weiter als einen dichten Haufen sehr zarter dunkler Molecularkörnchen eruirt. — Jedes Präparat muss mit Mühe von dem reichlichen *Fett* befreit werden, das theils in Form von doppelt und dunkelcontourirten Tropfen, in Form feiner Körnchen und Bläschen das Gesichtsfeld verdunkelt, theils auch über dem Wasser in blassgelben unregelmässigen bläschen- und inselförmigen Figuren (ohne Formveränderung) herumschwimmt; eine schillernde, aus erstarrtem Fett gebildete Oberhaut überzieht nebstdem in kurzer Zeit die der Ruhe überlassene Flüssigkeit, sowohl im Uringlase, als such am mikroskopischen Präparate. Nebst diesen Zersetzungsproducten haftet an zahlreichen Stellen der Schleifen und der Membranen, am häufigsten an deren Rändern ein schwarzkörniger *Detritus* und ein erdiges *Sediment*, dessen Vertrocknung einzelne spitzeckig quadratische *Krystalltäfelchen* zum Vorschein kommen lässt. Fig. 15. B. Ein dichtes Netz- und Strickwerk langfädiger ungemein zartcontourirter *Pilze*

haftet sehr innig an den Rändern der Membranen und Schleifen (Fig. 15 A*).

2. Die *zarten Flocken* von weisslicher oder gelblichweisser Farbe und lockerer Consistenz bestehen aus einer *dichten Zellenmasse*, an der nur hie und da ein kurzer Streifen einer faserähnlichen Zwischensubstanz haftet. Die Zellen erscheinen als compacte rundliche und oblonge Körper mit dunklen nicht scharf markirten Contouren und einem opaken feingranulirten Inhalt, der von der Hülle nicht scharf differenzirt sein kann, da weder die Isolirung der Zellen noch der Zusatz von Reagentien erhebliche Unterschiede zwischen Zellenmembran und Inhalt ersichtlich macht.

An vielen Partikeln bildet die Zellenmasse eine Art *plumper Faserzüge*, und dieses Bild erscheint um so weniger als blose Zufälligkeit, als besonders häufig keulenförmige und *papillenähnliche Endigungen* daran beobachtet werden. Fig. 16. Diese cohärenten, theils verzweigten, theils seicht gelappten Zellenvegetationen bieten allenthalben das *Bild von freigewordenen Pfröpfen*, wie man sie an der ausgetretenen Inhaltsmasse areolarer Maschengerüste zu sehen Gelegenheit hat. Sie hängen bald an einem zartgestreiften membranigen Gebilde (Bruchstück der höher organisirten Lagermasse, des Bindegewebs-Stroma), oder sie präsentiren sich ohne einen Stiel als terminale Vegetationen von einfacher Kolben- bis zur mikroskopischen Blumenkohlform, die von ihrem Mutterboden losgerissen, als nakte (jüngste) Zellenwucherung gedeutet werden können.

Zu dieser Annahme berechtiget einmal die constant und immer wiederkehrende *Anordnung* derselben in den angeführten Formen; — ferner die festelastische *Cohärenz* derselben, bedingt durch eine ungewöhnliche Dichtigkeit der ungemein spärlichen Intercellularsubstanz; — endlich der *optische Ausdruck* der Elemente selbst, der auf kein physiologisches Gebilde zu beziehen ist.

Denn abgesehen davon, dass die einzelne Zelle ein gewöhnliches Schleimkörperchen, mit dem es die nächste Aehnlichkeit haben könnte, an *Grösse* übertrifft (0,004'''—0,006'''), und sich von demselben durch eine mehr oblonge (mit dem längeren Durchmesser der Anordnungs-Richtung entsprechende) Form unterscheidet, so stellt sich bei genauer comparativer Prüfung eine unverkennbare Eigenthümlichkeit in dem *Lichtbrechungsvermögen* des ganzen Zellkörpers heraus, welche Erscheinung selbst den Elementen des dicksten blennorrhoischen Secrets, den Schleim- und Eiterzellen, unter ähnlichen Verhältnissen der Maceration nicht zukommt. Während diese bei längerer Stagnation aus ihrem Zusammenhange gelöst, gelockert, farblos, isolirt und arrodirt, ihr Inhalt zugleich in

Fettkörnchen desaggregirt erscheint, finden wir an den Zellen des vorliegenden Excretes einen ungewöhnlich innigen, selbst durch Reagentien nicht leicht lösbaren Zusammenhang, einen dunklen von der Peripherie gegen das Centrum allmälig heller werdenden Contour, eine hellgelbliche, mattglänzende Färbung des feinpunctirten Inhaltes, in welchem nach Zusatz von Essigsäure regelmässig ein centrales helles Kernkörperchen zum Vorschein kommt.

Noch deutlicher erscheint dieser Unterschied an jenen membranigen Lamellen, denen einzelne solche Zellen anhaften, die man entweder als die letzte *Spur eines Belegs oder einer Inhaltsmasse* des Lagergewebes ansehen kann, oder, wie dies an einzelnen Stellen, wo die streifige Schleife in eine dichtere Zellenmasse übergeht, den Anschein hat, als die *Grundlage der membranigen Gebilde* selbst zu deuten versucht wird. Dieses letztere beobachtet man bei medullaren Neoplasmen, besonders wenn sie in freie Hohlräume hinein wuchern, häufig genug; die nakte Zellenmasse nämlich, welche den häutigen Balken und fibrösen Strängen des Stromas aufsitzt, besteht theils aus persistenten, d. i. nicht weiter verwendbaren Elementen, theils aus solchen, die durch Schwund der Zellhülle und Identificirung des Zellinhaltes zu einer homogenen Membran sich umstalten. Die Letzteren und die persistenten Zellen lassen sich oft in der ersten Anlage gar nicht unterscheiden.

Bei einem abnormen Excret eines der unmittelbaren Ansicht nicht offen stehenden Organes ist man mehr als irgendwo versucht, dem *kleinsten Elemente eine specifische Natur* ansehen, und dessen Anordnung in der Art determiniren zu wollen, um die gewonnene Ansicht mit den übrigen Symptomen in Einklang zu bringen, namentlich das Ergebniss der manuellen Untersuchung damit bis zur klarsten Evidenz zu vereinbaren. Wir gestehen offen, dass es bei mikroskopischen Untersuchungen zuweilen schwer hält, sich nicht von solchen Rücksichten leiten und verleiten zu lassen, allein wir sind stets bemüht, von den mikroskopischen Bildern nur soviel abzulesen, als daran geschrieben steht. Die detaillirte Begründung der Ansicht über die Zellennatur eines im Excrete aufgefundenen Gebildes wird freilich zu weitläufig bei einem concreten Falle, aber diese Weitläufigkeit führt — wie jede Scala von Möglichkeiten bei ähnlichen Detailuntersuchungen — einzig und allein zum sicheren Ziele. Wenn man sich vergegenwärtigt, in

welchen Consistenzgraden, Farben- und Formverschiedenheiten nur z. B. *Schleim- und Eitermassen* vorzukommen pflegen, wird man die Möglichkeit eines Irrthums in einem Falle, wie dieser ist, nicht abweisen können, vielmehr einsehen, dass nur eine allseitige Prüfung und das controllirende *Experiment* (Untersuchungen an ähnlich macerirten Schleim- und Eitermassen) vor Verwechslungen *sichert.*

Die Schleim- und Eitersecretion ist bei Steinbildung in der Harnblase nicht blos ein mikroskopischer, viele Eigenthümlichkeiten bietender Befund, sondern es gibt Fälle, wo diese Secretion zum eminenten Symptom wird und selbst den *Stein zu maskiren* im Stande ist, wie von H. Prof. Pitha mitgetheilte Fälle beweisen, wo nicht blos Dupplicaturen der hypertrophirten Schleimhaut den Stein zu decken, sondern selbst dicke Schleimschichten denselben so einzuhüllen vermochten, dass anfänglich die Sicherstellung der Diagnose mittelst des Katheters unmöglich war. Katheter und Mikroskop könnten in solchen Fällen ein Pseudoplasma vortäuschen, wo die genauere Umsicht einen Stein mit einer Schleimhülle entdeckt. Andererseits kommt es in der That auch vor, dass sich Pseudoplasmen der Harnblase namentlich der dendritisch organisirte oder zottig aufgerissene Medullarkrebs, mit Sedimenten incrustiren, und dem eingeführten Katheter einen Befund bieten, wobei die Annahme eines sogenannten Steines von weicher Consistenz, wenn nicht gerechtfertigt, doch erklärlich erscheint. Der Katheter wird besonders in solchen Fällen vom Mikroskop unterstützt werden, so wie seine Controlle in den vorhergenannten Fällen von Nutzen ist. Auch in dem gegenwärtigen Falle war früher die Diagnose der sonst geschickten Chirurgen auf „einen Stein von weicher Consistenz“ gestellt worden, und es ist möglich, dass sie auf einem solchen allenfalls vorhandengewesenen Incrustat des Pseudoplasmas beruhte.

Bei der am letzten Tage vor der Abreise der Patientin vorgenommenen Untersuchung des Excretes, fand man neben den bereits beschriebenen Partikeln noch eigene merkwürdige Gebilde; es sind dies die drei bereits oben erwähnten zapfenförmigen *blutstrotzenden Bläschen* von Mohnkorn- bis Hirsekorngrösse (siehe oben Fig. 14 c.). — Sie sitzen, wie gesagt, an einzelnen Stücken mit fadendünnen Stielchen von $^1/_2$—1 Lin. Länge in Traubenform dicht beisammen; an anderen isolirten haften zackig eingerissene gefaltete Membranchen. Mit blutig imbibirten Gerinnseln oder mit blutführenden Fibrin- oder

Exsudat-Coagulis haben sie wohl eine äusserliche Aehnlichkeit. Der Unterschied stellt sich jedoch leicht heraus, wenn man einzelne dieser Bläschen näher betrachtet. Hie und da löst sich von deren Oberfläche ein *zartfaseriges Netz* los, (Fig. 17) bestehend aus einer fibrillären Bindesubstanz mit oblongen Kernen. Nach Entfernung dieser lockeren Hülle findet man ein *dünnhäutiges prallgespanntes Bläschen*, dessen blutiger Inhalt sich nur beim Einreissen der Membran entleert. Die letztere besteht wie die oben beschriebenen Schleifen aus einer streifigen durchsichtigen Grundsubstanz mit einer eingetragenen wohlerhaltenen Kernlage. Das Blut verhält sich ganz normal; in der den Stiel bildenden Partie gewahrt man in einer membranigen Grundlage mehrfache fortlaufende Contouren, anscheinend von parallel dahinziehenden Röhrchen. Wie dem auch sei, so ist der Nachweis der *bläschenartigen Blutbehälter* ein sehr wichtiges Moment zur Diagnose des Gebildes, das sich auf keine physiologische Thatsache eines ähnlichen Vorkommnisses zurückführen lässt.

Wir haben bei der Durchsicht der membranigen Flocken eine besondere Aufmerksamkeit darauf gerichtet, ob nicht ähnliche *mikroskopische* Blutcysten anfzufinden wären. Es gelang uns nicht, eine Spur davon zu entdecken, und der Umstand, dass kleine Partikel eines blutreichen Gewebes, vom Mutterboden losgerissen, und durch längere Zeit in einer faulenden Flüssigkeit suspendirt, ihren Blutinhalt gröstentheils abgeben müssen, erklärt es wohl, dass wir weder in den zarten collabirten Membranchen Bluträume, noch in den allenfalls als zuführende Gefässe anzusprechenden Röhrchen einen Blutinhalt nachweisen konnten.

Wir kennen bereits die *Haematurie* als ein vorstechendes Symptom des Harnblasenkrebses, und bezeichnen als Quelle derselben die zahlreichen *Endschlingen* des hochgradig vascularisirten *Zottenkrebses* (dendritischer Typus), oder das lockere durch Maceration ungemein leicht einreissende *Maschengerüst* des wenn auch weniger reichlich vascularisirten Medullarkrebses (areolarer Typus). In diesem Falle nun, der in Bezug auf den begränzten Standort des Pseudoplasmas zugleich der erste und einzige ist, der ein weibliches Individuum betrifft, finden wir *areolare und bläschenartige Bluträume* von dem membranigen Gerüste auswachsend, als die, wenn nicht alleinige, doch ausgiebigste Quelle der Blutung.

Dergleichen Blutbehälter kommen unter verschiedenen Formen bei vielen Neoplasmen, besonders bei den in einen hohlen Raum hineinwachsenden und rasch wuchernden Medullar-Carcinomen vor, sie sind daselbst von Apoplexien wohl zu unterscheiden, obwohl der Nachweis des Unterschiedes zwischen apoplektischen Herden und primitiv als solchen bestehenden Bluträumen nicht immer leicht zu geben ist. Nachgewiesener Massen stellen sie bald einfache *Cystchen* dar, deren *neogener Blutinhalt* ohne Gefässcommunication mit dem Mutterboden gleichsam die Krebsmasse ersetzt, welche benachbarte cystenartige Räume (Areoli) erfüllt; — bald sind sie ein Derivat von den *Gefässzweigen* des Gerüstes, deren terminale Schlingen regelmässig ein stärkeres Caliber besitzen als das zuführende Gefässrohr; — bald entwickeln sie sich als *Varicositäten*, sei es, dass durch einen local vorkommenden stärkeren Druck der Blutsäule, oder durch geringere Resistenz der benachbarten Gewebspartien eine Ausbuchtung der an sich nicht erkrankten Gefässwand ermöglicht wird, wie man dies auch bei der Bildung der cavernösen Blutgeschwulst beobachtet; — da wo sich ein Theil eines lockeren Pseudoplasmas durch Nekrose oder wie immer von dem Ganzen loslöst, können die demselben entsprechenden später obliterirenden Gefässe eine ähnliche cystenartige Erweiterung zeigen, die sich vom Mutterboden aus über das Niveau des Substanzverlustes erhebt (an einem Magencarcinom beobachtet); — in vielen Fällen endlich muss es unentschieden bleiben, ob die Blutsäckchen eines länger bestehenden Neugebildes dem einen oder dem andern Umstand ausschliesslich, oder mehreren zugleich ihre Entstehung verdanken.

Schliesslich sei noch bemerkt, dass an einem der zuletzt zur Untersuchung gekommenen Objecte — einem fingerdicken Klumpen von faserigem Riss und Bruch, und von der Consistenz eines lockeren Fibrin-Coagulums — eine durch Maceration viel weniger als in der anfänglichen Beobachtung entstellte Zellenmasse zur Ansicht kam, die sich durch ihre *excessive Grösse*, durch *Polymorphie*, durch *Spaltung* der grossen *Kerne* und durch einen in der parallelen Anordnung der langgestreckten Formen begründeten *Zusammenhang in faserähnlichen Zügen* auszeichnete (Fig. 18). Diese Eigenthümlichkeiten, die dem Carcinom par excellence zukommen, bekräftigten die bereits gestellte Diagnose um so mehr, als uns bald darauf ein ähnliches Object von der II. Int. Abth. des Hrn. Prof. Halla

zur Untersuchung zugekommen, durch eine frappante Aehnlichkeit mit diesem Falle die völlige Gewissheit über die richtige Deutung verschaffte. Wir lassen diesen Fall in Kürze folgen.

Zehnter Fall. Medullarkrebs der Harnblase bei einer Frau. (Z. N. 58. P. N. 5084 Mai, 1855, gestorben am 26. Juli 1855).

E. J. 38 J. alt, lang, hager, anämisch. Seit dem 30. Lebensjahre verheirathet, abortirte binnen 4 Jahren zweimal, das erstemal im 3., das zweitemal im 4. Monate der Schwangerschaft, ist gegenwärtig Mutter eines 4jährigen gesunden Mädchens, leidet seit zwei Jahren an Hämaturie und zuweilen an Strangurie, hat seit einem Jahre Unordnung und Ausbleiben der Menstruation bemerkt, und beim Coitus einen sonst ungewöhnlichen Schmerz in der Blase empfunden. Nebst Blut gingen auch ganze Stücke einer weichen, übelriechenden Substanz ab, die den Harnabgang zuweilen so erschweren, dass er mit dem Katheter entleert werden muss. In der letzten Zeit treten auch Rücken-, besonders Kreuzbeinschmerzen ein. Die Behandlung mit kalten Sitzbädern hatte der Blutung auf einige Zeit Einhalt gethan, doch bleibt der Harn immer trübe, flockig schleimig, penetrant übelriechend. *Manuelle Untersuchung:* Bauchdecken gespannt, orif. urethrae weit, an den Rändern geschwollen, sehr empfindlich, die Urethra erweitert, in der Tiefe dem Katheter ein leichtes Hinderniss bietend, über welches jedoch das Instrument leicht weggleitet. Die vordere Vaginalwand in der ganzen Breite leicht nach hinten gewölbt, ohne knollige Protuberanz, ohne Substanzverlust. Fornix vaginae geräumig, Vaginalportion des Uterus stark entwickelt, Orif. ut. ext. klaffend, an den Rändern gekerbt. Bei dem Versuch, die hintere Symphysenfläche von der Vagina aus zu befühlen, findet man einen weichen, zusammendrückbaren Körper eingelagert, der nur der Harnblase, einem Theile der hintern untern Blasenwand und dem Anfangstheil der Urethra zu gehören scheint; Berührung daselbst sehr schmerzhaft.

1. *Mikroskopische Untersuchung* 15. *Mai.* Fetzig gerissene Klumpen, ähnlich den Fibringerinnseln im rechten Herzen. Faseriger Bruch und Riss, blassgelbe Farbe mit dunkelrothen Blutpunkten und Streifen im Innern. Elemente: Polymorphe Zellen von colossalen Dimensionen, mit sehr grossen ein- und mehrfachen Kernen; die in die Länge gezogenen Formen gleichen den grossen Epitelialzellen der Zungenschleimhaut (namentlich deren Papillen). Anordnung zu faserförmigen Strängen, zwischen denen die rundlichen Formen der Elemente und reichliche lose Kerne dicht gedrängt lagern. — Die blutigen Streifen und Punkte sind *spindelförmig erweiterte Capillargefässe* von namhaftem Caliber, mit einfachen Wandungen und unverändertem Blutinhalt. Nebstdem kommen vor: Schleimzellen, lose und in membran-ähnlich zusammenhängenden Flocken, Trippelphosphate, prismatische Krystalle mit vollkommen glatten Flächen und scharfen Kanten; erdiges Sediment und Fett.

2. *Mikroskopische Untersuchung* 17. *Mai.* Ein schleimähnlicher Flocken mittelst eines feinen Pinsels entwirrt, stellt einen dichten Haufen papillarer, stellenweise dendritisch auswachsender Gebilde dar, deren Caliber von der Basis bis zur Spitze gleichmässig 0,025—0,030‴ beträgt. Die

Contouren zart und blass, in der Fläche feinpunktirte Streifung, die im Centrum dunkler, gegen den Rand heller erscheint. Hie und da undeutliche Umrisse rundlicher Kerne, die zwischen den Vegetationen lagern. In einer solchen von stärkerem Caliber eine dicht gedrängte Masse grosser, rundlicher Kerne mit deutlichen Nucleolis, das ganze mit einem breiten, helleren, deutlich gestreiften Saume umgeben. Das Präparat nebstdem von reichlichen, länglichrunden Kernen umspült, die sich durch ihre Form, scharfe Contouren und einfache Nucleoli von den gleichgrossen Schleimzellen unterscheiden. — Krystalle von Phosphaten, prismatische, Dach- und Sargdeckel - Formen, wie früher.

3. *Mikroskopische Untersuchung* 18. *Mai.* Ein ähnlicher Flocken wie in der vorherg. Angabe, zeigt (bei einer Vergrösserung von 180. Plössl) einen zarten Pennicill ähnlicher papillärer Auswüchse, welche einseitig auf einem gemeinschaftlichen Stiele aufsitzend, die Fächerform der Fühler mancher Insecten nachahmen. An den abgerissenen Stücken bemerkt man die bei verschiedener Einstellung des Rohres abwechselnd wahrnehmbaren, übereinander laufenden Contouren (Zacken und fibrillären Fransen) eines Röhrchens. — Der Inhalt der unverletzten Schläuche undeutlich streifig, die äussere Kernmasse und dazwischen liegende Krystalle wie oben.

4. *Mikroskopische Untersuchung.* Fingerdicke hyaline Schleimklumpen, gallertähnlich zitternd und zusammenhängend. Man findet darin areolare Räume (Fig. 19), umgeben von einer dichteren, von erdigen Sedimenten durchsetzten und dunkelgranulirten Bindemasse, erfüllt von theils formlosem, theils morschen Schleimkörpern ähnliche Zellen enthaltendem sulzigem Stoff. — Erdiges Sediment, prismatische Krystalle in Menge. An einem andern dichten, zackig rissigen Klümpchen von Weizenkorngrösse, gelblicher Farbe, ist ein faseriger Bruch wahrnehmbar, bedingt in der secundären Anordnung der Formbestandtheile zu Fibrillen. Diese bestehen theils aus polymorphen kernhaltigen Zellen, theils aus blossen, gegen Essigsäure resistenten Kernen (von der Grösse normaler Schleimkugeln), die um einen centralen Strang feiner, scharf contourirter Fäserchen dicht gelagert, und stellenweise so innig mit demselben verwebt erscheinen, dass eine strenge Sonderung der länglich ausgezogenen Zellen- und Kerngebilde von den kurzen Fasern gar nicht möglich ist. — An den freien Enden ist die Auffaserung sowohl der zelligen als der fibrillären Elemente ersichtlich, und die Uebergangsformen zwischen beiden deutlich wahrnehmbar.

Das Zusammenfassen dieser Membra disjecta zu einer histologischen Gesammtdiagnose, ist nach in den früheren Fällen entwickeltem Vorgange leicht zu treffen. — Die Besitzerin dieses letzten Vorkommnisses ging nach 4monatlicher, nicht zu stillender Blutung an Anämie zu Grunde. Die Autopsie wurde aus religiösen Rücksichten der israelitischen Angehörigen nicht gestatet.

Wir wollen zum Schlusse die in der Harnblase überhaupt vorkommenden Neubildungen, welche die Aufmerksamkeit des praktischen Arztes in Anspruch nehmen, aufzählen, und einige Bemerkungen daran knüpfen, die sich aus der Betrachtung

der uns zur Kenntniss und Ansicht gekommenen Präparate ergeben.

Mit Ausschluss der allgemeinen Hypertrophie der Schleimhaut, wie sie nach chronischer Entzündung, bei Steinbildung oder Hindernissen in der Urethra vorkommt, ist es vor Allem die *villöse* und *lamellöse Hypertrophie* — in Form von zarten, bis 6 Linien langen Zotten und Falten, ähnlich denen an der Darmschleimhaut — und dann der *Schleimhautpolyp*, in Form von gestielten Geschwülsten. Das interessanteste Präparat dieser letzteren Kathegorie, was uns zur Kenntniss gekommen, ist das im Stockholmer Museum befindliche und von C. Santesson als Calculus vesicae pedunculatus beschriebene (Mus. anat. Holmiense. Fasc. I.). Ein fibröser Polyp von Hühnereigrösse mittelst eines rundlichen etwas gewundenen Stieles von der hintern obern Wand der hypertrophirten Blase herabhängend, gibt den Kern ab, um den sich das Concrement bis mehr als zu Ganseigrösse anlagert, und so in der starrwandigen Höhle suspendirt erscheint. Ueber die Lebens- und Krankheitsgeschichte des alten Weibes von der Insel Hisingen, in deren Leiche man diesen gestielten Stein gefunden, existirt weiter keine Notiz.

Das *Papillom*, die papillare Geschwulst, bildet einerseits einen ebenso natürlichen Uebergang in die villöse Hypertrophie der Blasenschleimhaut, als sie andererseits vom *Zottenkrebs* in manchen Fällen schwer zu trennen ist. Rokitansky und Schuh (Pseudoplasmen, Wien 1854, wo S. 430 die mikroskopische Diagnose am Krankenbette angedeutet wird) fassen unter dem Zottenkrebs beides zusammen; Virchow und Förster (Spec. Pathol. Anat. S. 389, und Allg. P. Anat. S. 208, sammt Quellenangabe) unterscheiden beide Formen genau von einander. Wir theilen die Ueberzeugung der Letzteren, dass es in der Blase Papillome gibt, die mit dem Krebs nichts gemein haben, als die äussere Form (S. oben S. 21); sie kommen an Schleimhäuten sowohl als an serösen Flächen vor, und in kleinem Massstab haben wir sie an dem Endocardium, als büschel- oder pinselförmiges Papillom der Aortenklappe an den einzelnen Zipfeln unterhalb der Arantischen Knötchen, einigemal untersucht und als Präparate aufgehoben. — Die klinische Angabe über den Verlauf solcher Neubildungen kann für den Anatomen kein Eintheilungsprincip abgeben, denn in dieser Beziehung ist vieles *bösartig* ohne in die Kathegorie des Carcinoms zu gehören; die Schwierigkeit besteht vielmehr darin, dass die wichtigsten anatomischen Merkmale des Carcinoms: die Nichtzugehörigkeit des Gewebstypus zum Mutterboden, und die massenhafte Production von persistenten, d. h. einer höheren Gewebs-

fähigkeit ermangelnden Zellen, dem Papillom in einem gewissen Grade ebenso zukommen können, wie sie ein unzweideutiger Zottenkrebs in exquisiter Weise bietet. Es sind weder die Villositäten, noch die Wucherung eines rasch zerfallenden Beleges von polymorphen Zellen dem physiologischen Zustand der Blasenschleimhaut zugehörig. — Der praktische Arzt mag übrigens über diese histologischen Schwierigkeiten ruhig hinweggehen, und seine Aufgabe nicht aus dem Auge verlieren, nämlich, die materiellen Bedingungen der Urämie und besonders die Quelle der Haematurie nachzuweisen, die das bösartigste Symptom aller dieser Neubildungen ist. — Dem *Medullarkrebse* kommt vorzugsweise eine reichliche Prolification und Dissemination, sowie das topographische Vordringen durch die verschiedenen Schichten der Beckenorgane (Rectum, Uterus, Vagina, Harnblase) zu. Die Behandlung desselben ist immer nur symptomatisch; denn wenn es je möglich wäre, ein dünngestieltes Pseudoplasma als solches zu erkennen, und durch die Ligatur oder wie immer zu entfernen, so wäre das noch kein radikales Mittel gegen den Krebs. Und die gebenedeiten Mittel von Landolfi helfen da auch nichts; denn man müsste — abgesehen von der Unmöglichkeit der localen Application — ebensowenig wie Landolfi den Krebs kennen, und nicht wissen, dass der Letztere wol *geäzt* aber nicht *geheilt*, in seinen Recidiven nicht bezwungen werden kann. — Der *Tuberkel* erscheint in Form zerstreuter Knötchen mit baldiger Geschwürsbildung. — Hyaline, perlgraue, discrete *Bläschen* kommen zuweilen im Trigonum, mit und ohne Katarrh vor; *seröse Cysten* von nahmhafter Grösse sah ich nie, wohl aber *Colloidcystchen* von Erbsen- bis Bohnengrösse mit einem honiggelben, dicklichen Inhalt; bei einem Weibe ging ein solches vom unteren Umfang des Orific. int. aus und hatte das Lumen des Rohres bedeutend beeinträchtigt.

Die *Hypertrophie der Prostata*, gleichfalls ein Hinderniss der Harnsecretion, habe ich durch fünf Jahre an der hiesigen Anstalt in folgenden Formen und Verhalten gesehen: *a)* einfache *gleichmässige Hypertrophie* der ganzen Drüse mit bedeutender Verengung der pars prostatica. — *b) die einseitige Hypertrophie* mit seitlicher Prominenz in den Canal und Krümmung und Verengung desselben. — *c) die knollige Hypertrophie* mit Prominenz in das Collum vesicae. In einem Falle, wo der Knollen breit aufsass, gab er kein Hinderniss ab; in einem andern Falle, wo er, dünngestielt und beweglich, tief in die Blase hineinragte, wurde das Ostium inter. durch denselben verlegt. Urocystitis, Nephritis und Uraemie waren die Folgen. — *d)* die knollig-*lappige* Hypertrophie mit Prominenz

in die Blase. Ein Fall von *dreilappiger* (am Durchschnitt kleeblattförmiger) Hypertrophie war ein zufälliger Befund, d. h. die breit ausgehende, starre Geschwulst gab — im Widerspruche zu den Angaben der Autoren — bei Lebzeiten kein Hinderniss ab, — *e)* In einer der jüngsten Beobachtungen war es weniger die gleichmässige Massenzunahme der Drüse, als vielmehr ein *ringförmiger Wulst* von 2 Linien Dicke, der von der Prostata ausgehend das trichterförmige Ostium int. bis zum Durchgang einer Sonde beengte, und an dem aufgeschnittenen Rohre die Form einer Klappe nachahmte.

So mannigfach der Befund erscheint, der sich bei einer erkrankten Blase der genauen physikalischen Untersuchungsmethode ergibt, so unerklärlich bleibt doch ein Zustand, über den die langjährige Erfahrung der routinirtesten Chirurgen berichtet; nämlich, die Symptome von Blasenstein ohne Vorhandensein eines fremden Körpers in der Blase. Vor einigen Jahren wurde auf der hierortigen Klinik ein junger Bursche durch lange Zeit von H. Prof. Pitha und dem damaligen Assistenten, gegenw. Prof. Morawek in Würzburg, beobachtet und als Steinkranker befunden. Als man zur Operation schreiten wollte, fand man den Stein nicht; man stand vom Steinschnitt ab, und die Symptome der Krankheit kamen nie wieder. — Nach einer Mittheilung des H. Dr. Magni aus Florenz hatte der berühmte Prof. Regnoli an einem 8jährigen Knaben die Steinoperation verrichtet, ohne den Stein gefunden zu haben; der Knabe genas, und sein Leiden wich für immer. — In Paris hörten wir in einer Vorlesung von H. Civiale (Ende August 1855) eine Erwähnung von der Möglichkeit einer solchen Täuschung, — und H. Prof. Sangalli aus Pavia erinnerte sich bei dem Besuche unseres Museums eines ähnlichen Falles an der dortigen Hochschule wie der von Florenz berichtete lautet. Bei aller Vorsicht, mit der man beim Katheterismus jede Selbsttäuschung vermeidet, mit der man selbst den Ring vom Finger abnimmt, — stosst man doch auf ein Räthsel, das seine Lösung von der Zukunft erwartet.

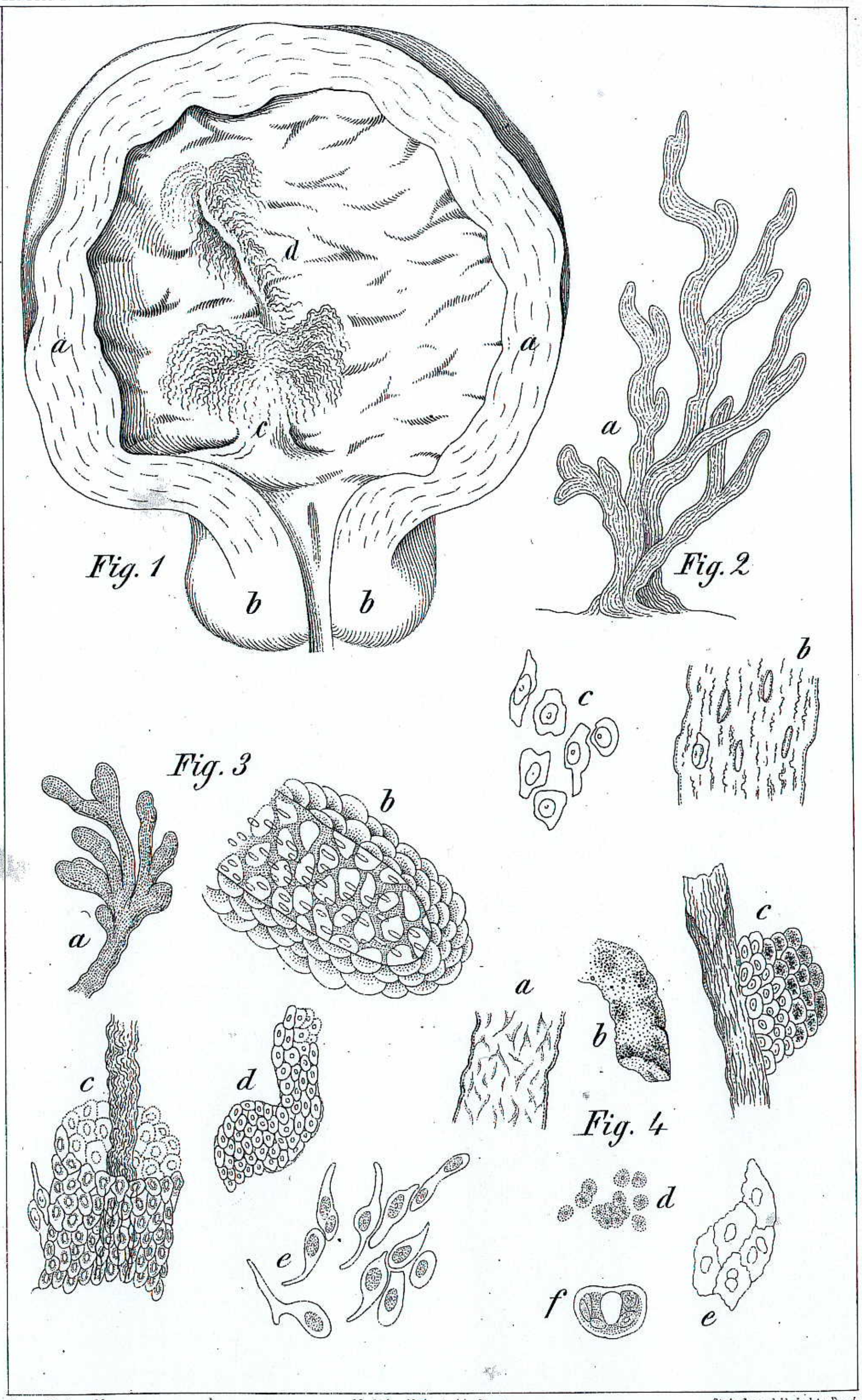

Gez. v. Dr. Lambl. Verlag v. K. André in Prag. Steindr. v. J. Habel in Prag

Fig 1 u. 2 Seite 3. – Fig 3 u. 4 Seite 5.

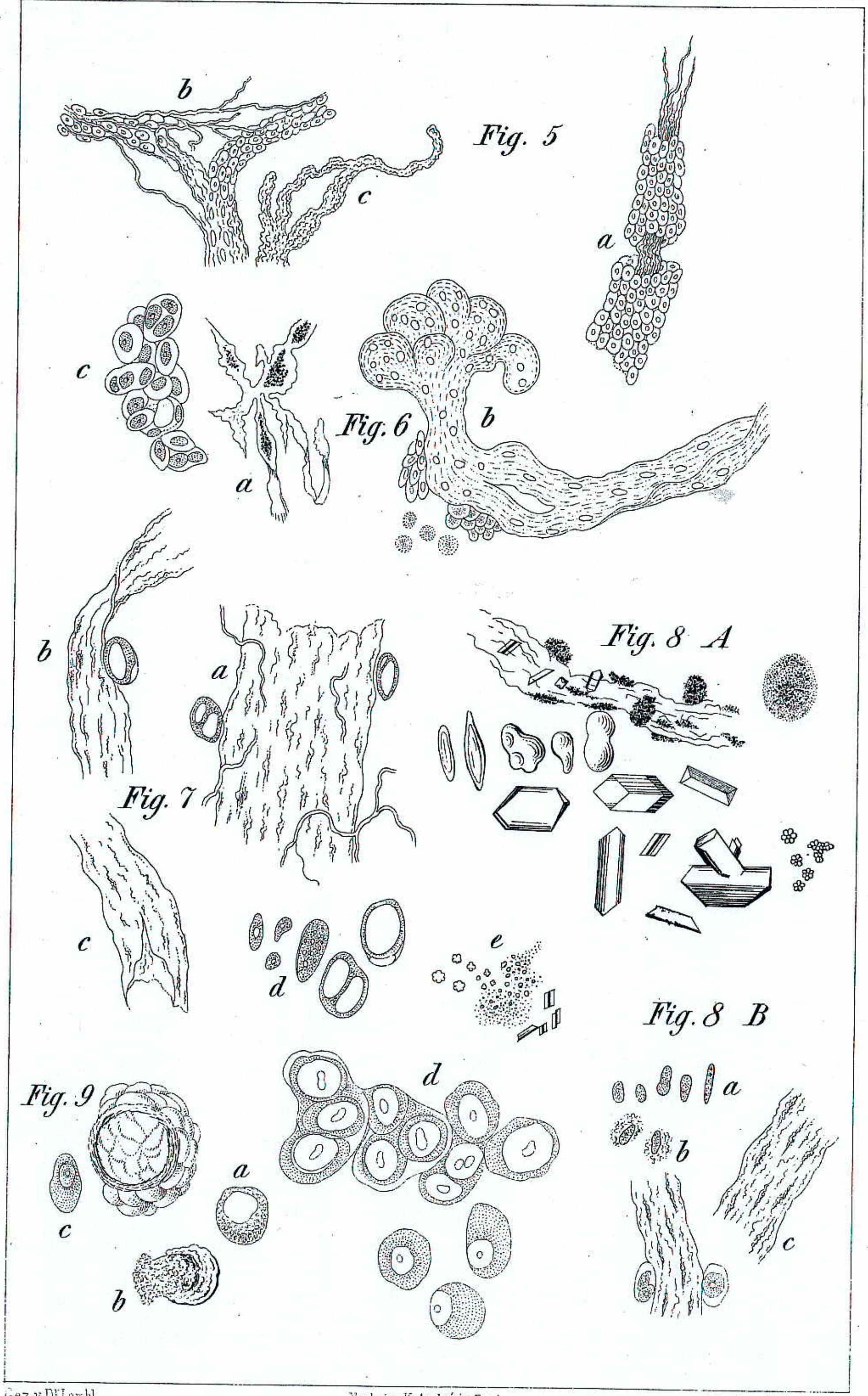

Gez. v. Dr. Lambl. Verlag v. K. André in Prag. Steindr. v. J. Habel in Prag.

Fig. 5 u. 6, Seite 5.- Fig. 7 Seite 6.- Fig 8 A.B. Seite 8.- Fig 9 Seite 9.

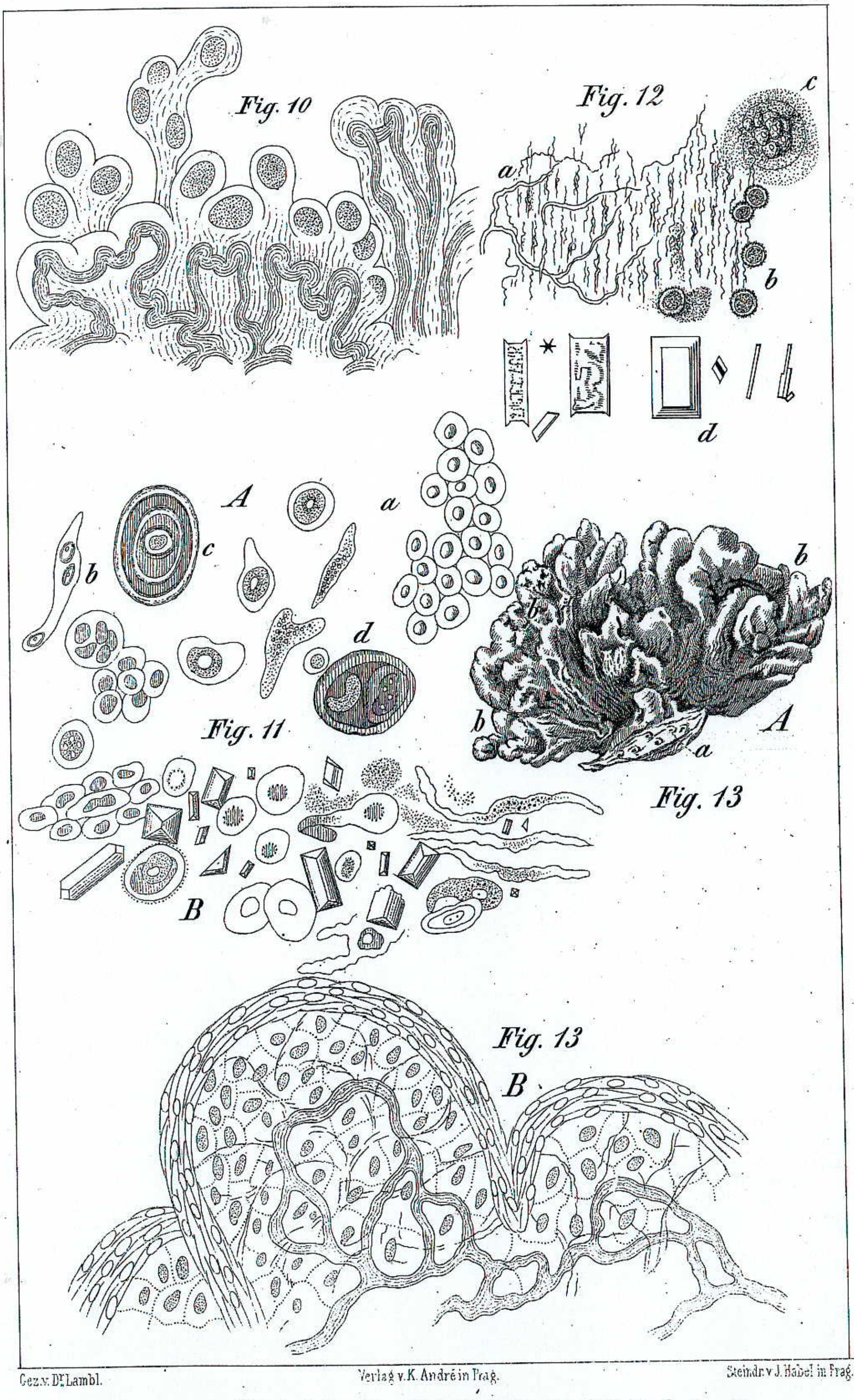

Gez. v. Dr. Lambl. Verlag v. K. André in Prag. Steindr. v. J. Habel in Prag.

Fig. 10, Seite 11.– Fig. 11, Seite 12.– Fig. 12, Seite 17.– Fig. 13, Seite 21.–

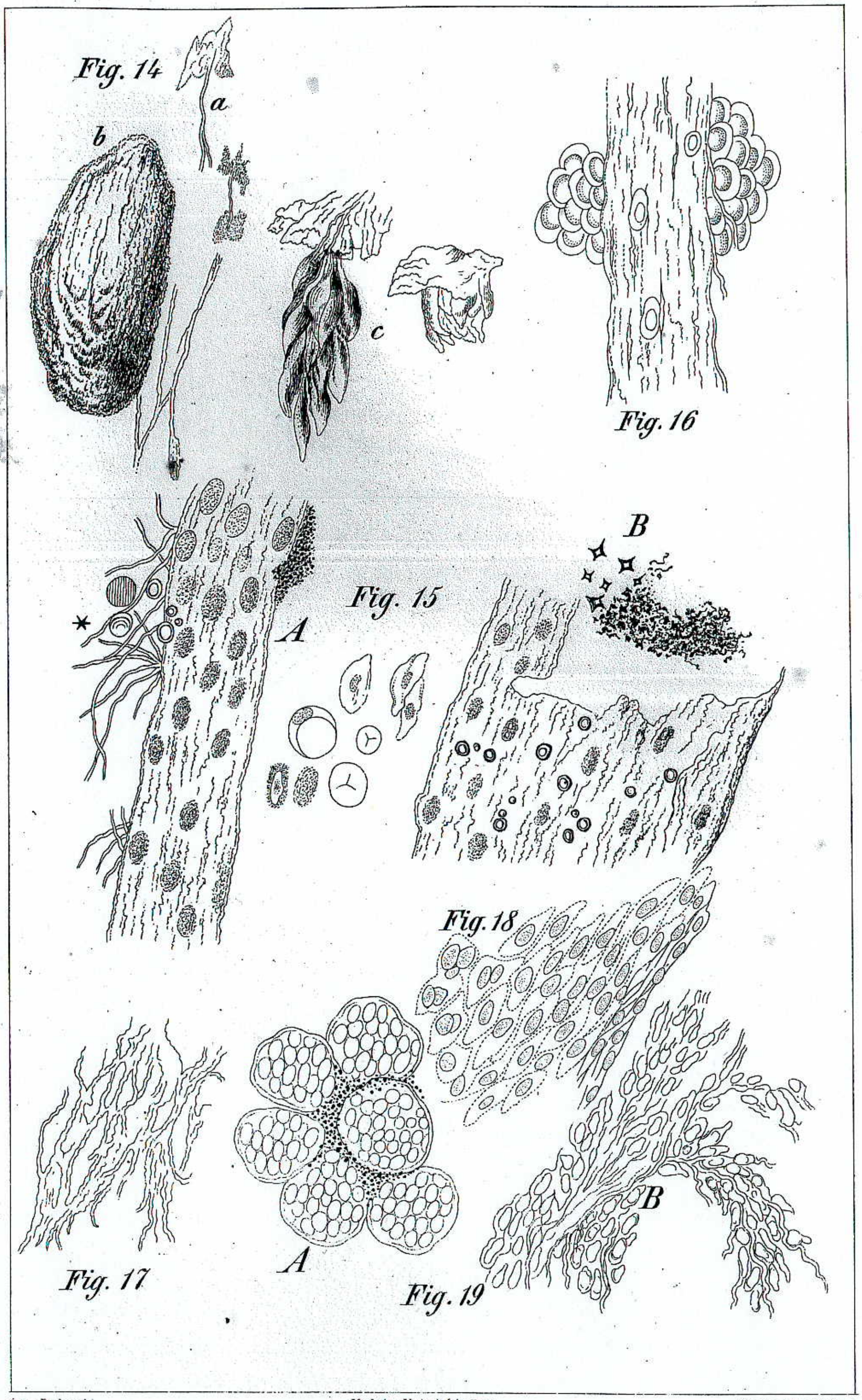

Gez. v. Dr. Lambl. Verlag v. K André in Prag. Steindr. v J. Habel in Prag.

Fig. 14 u. 15, Seite 22 u. 25.- Fig. 16, Ste. 24 - Fig. 17, Seit. 26 - Fig. 18, Seit. 27 - Fig. 19, Seite 29. —
